# Staphylococcal phages as agents of evolution and innovation: From gene flow to next-generation therapeutics

**DOI:** 10.1590/1678-4685-GMB-2025-0131

**Published:** 2026-02-09

**Authors:** Ciro César Rossi, Felipe Castro Oliveira de Brito Teixeira, Giarlã Cunha da Silva, Monalessa Fábia Pereira, Marcia Giambiagi-deMarval

**Affiliations:** 1 Universidade Federal Fluminense, Departamento de Biologia Celular e Molecular, Niterói, RJ, Brazil.; 2 Universidade Federal de Viçosa, Departamento de Bioquímica e Biologia Molecular, Viçosa, MG, Brazil.; 3 Grisea Biotecnologia, Rio de Janeiro, RJ, Brazil.; 4 Universidade Federal de Viçosa, Departamento de Microbiologia, Viçosa, MG, Brazil.; 5 Universidade do Estado de Minas Gerais, Departamento de Biologia, Belo Horizonte, MG, Brazil.; 6 Universidade Federal do Rio de Janeiro, Instituto de Biodiversidade e Sustentabilidade (NUPEM), Macaé, RJ, Brazil.; 7 Universidade Federal do Rio de Janeiro, Instituto de Microbiologia Paulo de Góes, Rio de Janeiro, RJ, Brazil.

**Keywords:** Antimicrobial resistance, bacteriophage therapy, phage genomics, Staphylococcus, translational microbiology, business

## Abstract

*Staphylococcus* species include both well-known pathogens and overlooked reservoirs of antimicrobial resistance. With rising resistance rates and limited treatment options, especially for methicillin-resistant strains, interest in alternative therapies has resurged. Among them, bacteriophages (phages) are promising biological agents due to their high specificity, low toxicity, ability to disrupt biofilms, and co-evolution with bacterial hosts. This review explores the biology, pan-genomics, diversity, and therapeutic relevance of staphylococcal phages. We revisit their historical discovery and re-emergence as tools against multidrug-resistant infections, highlighting morphological features, replication strategies, and recent taxonomic updates. Genomic analyses reveal distinct clusters of genome sizes, rare presence of resistance genes, and implications of transduction, bacterial defense systems, and phage-encoded anti-defense mechanisms. Preclinical studies show broad host range and synergistic activity with diverse antimicrobial agents, while engineered phage enzymes expand therapeutic possibilities. Clinical evidence, though limited, supports safety and efficacy in compassionate-use cases and early trials targeting *Staphylococcus*. Finally, we examine business models translating phage innovation into applied therapies, emphasizing regulatory, logistical, and financial challenges. In this broader context, phage technologies are not just alternatives to antibiotics-they represent an opportunity for innovation in global health. Their full potential depends on coordinated actions across science, industry, and policy.

## Introduction

Antimicrobial resistance (AMR) has become one of the most pressing challenges in modern medicine, threatening the effectiveness of conventional antibiotics and demanding the exploration of alternative therapeutic strategies. Within this scenario, *Staphylococcus* species stand out not only for their pathogenic potential but also for their remarkable capacity to acquire and disseminate resistance determinants across clinical, animal, and environmental settings (Rossi et al., 2020; Goulart, 2023). The global rise of multidrug-resistant strains, including methicillin-resistant *Staphylococcus aureus* (MRSA) and resistant coagulase-negative staphylococci, underscores the urgency for innovative solutions (Michels et al., 2021; WHO, 2024).

Bacteriophages - viruses that specifically infect bacteria - have resurfaced as viable candidates for controlling staphylococcal infections. Beyond their therapeutic potential, phages also serve as powerful tools to understand microbial evolution, gene transfer mechanisms, and the arms race between host defenses and viral countermeasures (Haaber et al., 2017; Bernheim and Sorek, 2020; Pinilla-Redondo et al., 2020; Silva and Rossi, 2025). Their unique biology, host specificity, and genetic diversity have opened new avenues for applications in human and veterinary medicine, agriculture, and biotechnology.

This review provides an integrative overview of staphylococcal phages, from their discovery and life cycles to their genomic architecture and clinical relevance. We explore recent advances in preclinical research, translational strategies, and the diversification of business models supporting phage-based innovation. Together, these aspects highlight the growing role of phages not only as therapeutic agents but as strategic assets in the broader response to antimicrobial resistance.

## Staphylococcal infections and the narrowing of therapeutic options

Bacteria from the genus *Staphylococcus* are commonly part of the normal microbiota of humans and animals, inhabiting the skin and mucosal surfaces (Ondusko and Nolt 2018). However, under certain conditions, several species can shift from harmless colonizers to opportunistic pathogens capable of causing a variety of infections, ranging from skin abscesses to severe systemic conditions such as bacteremia and pneumonia (Pickens and Wunderink, 2022; Lam and Stokes, 2023; Linz et al., 2023). Around 40% of all known *Staphylococcus* species are established human pathogens, although most are associated with sporadic infections (Bartlett et al., 2022). Additionally, species previously classified within the genus *Staphylococcus* but now recognized as members of the genus *Mammaliicoccus*, also within the family *Staphylococcaceae*, include phylogenetically related and pathogenic species, particularly *Mammaliicoccus sciuri* (Madhaiyan et al., 2020).

*Staphylococcus aureus* is distinguished from many other species in its genus by its ability to produce coagulases, polypeptides that bind to and activate prothrombin, thereby converting fibrinogen to fibrin and promoting the clotting of plasma or blood. This creates a protective barrier around the bacterium that can help evade host immune responses (McAdow et al. 2012). Another coagulase-positive species of growing clinical relevance is *Staphylococcus pseudintermedius*, which primarily colonizes the skin and mucous membranes of domestic dogs (Lynch and Helbig, 2021). Traditionally considered an opportunistic pathogen in veterinary medicine, *S. pseudintermedius* has been increasingly reported in human infections, particularly among individuals with close contact with companion animals (Carroll et al., 2021; Moses et al., 2023). These zoonotic infections may manifest as wound and skin infections, otitis, and occasionally more invasive diseases, and are often caused by multidrug-resistant strains such as methicillin-resistant *S. pseudintermedius* (MRSP) (Lynch and Helbig, 2021).

In contrast, most *Staphylococcus* species are coagulase-negative (CoNS), a group that was historically considered clinically insignificant but is now known to include several important opportunistic pathogens. These species are frequent colonizers of human skin and mucous membranes and have adapted remarkably well to the hospital environment (Becker et al., 2020). *Staphylococcus epidermidis*, for example, is one of the leading causes of infections associated with indwelling medical devices due to its ability to form biofilms and resist host defenses (Severn and Horswill, 2023). *Staphylococcus haemolyticus* has been associated with bloodstream infections and is notable for its high resistance rates to multiple antibiotics (Rossi et al., 2024). *Staphylococcus capitis* is emerging in neonatal intensive care units and has shown concerning levels of resistance to antiseptics and antibiotics (Heath et al., 2023). *Staphylococcus saprophyticus*, although less commonly implicated in hospital settings, is a well-known agent of urinary tract infections, particularly in young women (Djawadi et al., 2023). Together, these CoNS represent an increasingly important component of the staphylococcal threat to public health.

Although the most severe infections are typically caused by a limited number of *Staphylococcus* species, growing evidence indicates that members of the family *Staphylococcaceae* share a wide array of mobile genetic elements (MGEs) through horizontal gene transfer (Rossi et al., 2020). This dynamic turns even less-studied species into important reservoirs of antimicrobial resistance genes, regardless of their host or environmental origin. Homologous MGEs have already been reported in isolates from animals, humans, and environmental sources, revealing a complex network of genetic exchange within this bacterial family (Carvalho et al., 2024). These findings highlight the relevance of a One Health approach to understanding staphylococcal ecology and evolution. The role of these organisms in storing and disseminating resistance determinants contributes significantly to the alarming spread of antimicrobial resistance, a central concern in global public health. 

The increasing prevalence of antimicrobial resistance among *Staphylococcus* species has led to serious concerns in clinical settings. Among the most critical threats are methicillin-resistant *S. aureus* (MRSA) and vancomycin-resistant *S. aureus* (VRSA), which are both classified by the World Health Organization as high-priority pathogens for research and development of new treatment alternatives (WHO, 2024). This is especially concerning because the emergence of resistance has not been met with a corresponding development of novel antibiotics. In Brazil, surveillance data indicate that over 50% of clinical *S. aureus* isolates are methicillin-resistant, further limiting therapeutic options (Lee et al., 2018).

Resistance, however, is not restricted to *S. aureus*. Coagulase-negative staphylococci (CoNS), such as *S. epidermidis* and *S. haemolyticus*, are increasingly associated with multidrug-resistant infections, particularly in healthcare settings. These species are among the staphylococci with the highest number of documented resistance mechanisms and are frequently implicated in infections involving prosthetic devices and catheters. Their ability to form biofilms and to acquire and disseminate resistance determinants makes them challenging nosocomial pathogens, often as difficult to treat as their coagulase-positive counterparts (Michels et al., 2021; Goulart, 2023).

As conventional antibiotics lose efficacy, treatment options are becoming increasingly limited, especially in the face of biofilm-associated infections and strains with extensive resistance profiles. This scenario has prompted a renewed interest in alternative approaches, including antimicrobial peptides (Ganesan et al., 2023), phage-derived enzymes (Golban et al., 2025), and, most notably, bacteriophage therapy (Atshan et al., 2023), which may offer promising routes for the control of multidrug-resistant *Staphylococcus* spp.

## The discovery, decline and renaissance of bacteriophages in antimicrobial therapy

Bacteriophages (or simply phages) - viruses that specifically infect bacteria - were discovered over a century ago, almost simultaneously by two scientists working in different contexts. In 1915, British bacteriologist Frederick Twort, while studying cultures of vaccinia virus, noticed that some colonies of micrococci became transparent and stopped growing. He found that this effect could be transferred to other bacterial cultures using a filtrate that passed through porcelain filters, suggesting the presence of a filterable, thermosensitive, and host-dependent agent (Twort, 1915). Although he could not determine whether it was a virus, a bacterial stage, or an autolytic enzyme, his work provided the first documented evidence of phages and introduced the idea of ultramicroscopic infectious entities.

In 1917, French-Canadian microbiologist Félix d’Hérelle made a decisive breakthrough. Investigating dysentery outbreaks at the Institut Pasteur in Paris, he isolated a “*microbe invisible*” from the feces and urine of recovering patients, which displayed specific lytic activity against the Shiga bacillus (i.e. *Shigella dysenteriae* type 1). He demonstrated that the agent passed through fine filters, required bacterial hosts to replicate, and formed clear plaques on solid media - conclusive proof of its viral nature. d’Hérelle also observed that it could be serially propagated, that host specificity could shift, and that lysates had immunogenic properties. He coined the term “bacteriophage” (bacteria-eater) and was the first to propose its therapeutic use (d’Herelle, 2007). 

Together, these discoveries laid the groundwork for the early development of phage therapy, which gained momentum in the pre-antibiotic era. Encouraged by d’Hérelle’s observations, early clinical trials of phage therapy began in the 1920s and 1930s, targeting infections such as dysentery, cholera, and staphylococcal skin conditions (Summers, 2001; Abedon et al., 2011). Reports of success, though sometimes anecdotal or lacking modern clinical rigor, drove worldwide interest in therapeutic applications.

Despite their early use for therapeutic purposes, the structure of phages was only visually confirmed in 1940, when H. Ruska used transmission electron microscopy to observe these viruses adsorbing to bacterial surfaces and inducing lysis (Ruska, 1940). These images showed spherical particles 40-80 nm in size and captured the disruption of host cells, providing the first direct visual evidence of bacteriophage activity and supporting their classification as autonomous viral entities.

However, amidst the rise of phage therapy, the discovery of penicillin by Alexander Fleming in 1928 (Flemming, 1929), and its widespread use starting in the 1940s, shifted the medical landscape dramatically. Antibiotics offered a broad-spectrum, standardized, and industrially scalable solution to bacterial infections, overshadowing the more complex and variable practice of phage therapy (Hutchings et al., 2019).

In much of the Western world, interest in bacteriophages declined rapidly. Despite this downturn, phage therapy remained active in several Eastern European countries, most notably in the Soviet Union and later in Poland and Georgia, where dedicated institutes like the Eliava Institute in Tbilisi continued to produce and research therapeutic phages throughout the 20th century (Kutateladze and Adamia 2008). These regions preserved valuable expertise and clinical experience that would later support the resurgence of interest in phages amid rising antibiotic resistance (Chanishvili, 2012).

In recent decades, the global rise of antimicrobial resistance (AMR) has emerged as a critical public health threat, undermining the efficacy of antibiotics and complicating the treatment of common infections. The World Health Organization (WHO) has identified several priority pathogens for which new treatments are urgently needed. Among the highest priorities are multidrug-resistant strains of *Acinetobacter baumannii*, *Pseudomonas aeruginosa*, and *Enterobacteriaceae* (including *Klebsiella pneumoniae* and *Escherichia coli*), as well as MRSA and vancomycin-resistant *Enterococcus* (VRE) (WHO, 2024).

This growing crisis has reignited interest in alternative antimicrobial strategies, including the therapeutic use of bacteriophages (Kortright et al., 2019). Phages offer a highly specific mode of action, the potential to co-evolve with bacterial targets, and effectiveness against biofilms and antibiotic-resistant strains. As conventional antibiotics continue to lose effectiveness, phage therapy is once again being explored with scientific rigor, now supported by advances in genomics, synthetic biology, and personalized medicine approaches (Cisek et al., 2017).

## Morphology, life cycles, bioproducts and classification of staphylococcal phages

The classification of bacteriophages has undergone substantial revision in recent years, particularly with the increasing availability of complete genome sequences. The International Committee on Taxonomy of Viruses (ICTV) has progressively adopted genome-based criteria, replacing the traditional morphology-centered approach. Current taxonomic frameworks consider parameters such as genome type and structure, phylogenetic relationships inferred from conserved marker genes or whole-genome comparisons, gene content similarity, and, to a lesser extent, virion morphology and host specificity (Turner et al., 2021). As of mid-2025, the ICTV classification included over 16,000 viral species distributed across nearly 400 families, available at https://ictv.global/taxonomy.

**Figure 1 -:**
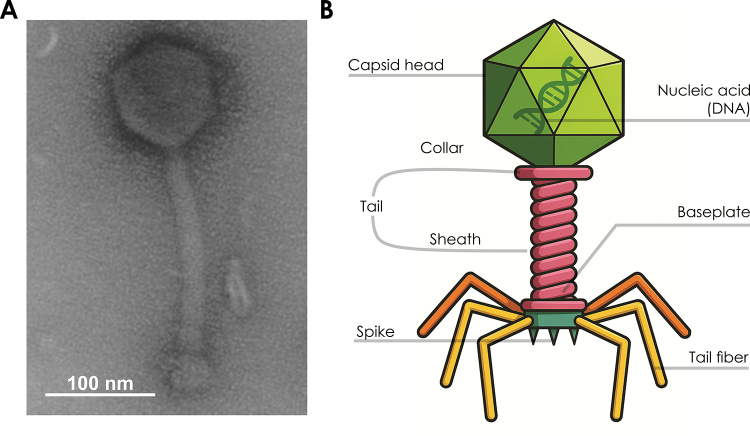
General structure of bacteriophages infecting *Staphylococcus*. (A) Transmission electron microscopy image of a bacteriophage isolated by our research group. (B) Schematic representation of the main structural components of *Staphylococcus*-infecting bacteriophages.

Although many *Staphylococcus*-infecting phages remain taxonomically unclassified, those with an assigned classification belong to a wide range of genera, including *Andhravirus*, *Baoshanvirus*, *Beceayunavirus*, *Biseptimavirus*, *Coventryvirus*, *Dubowvirus*, *Fibralongavirus*, *Kayvirus*, *Peeveelvirus*, *Phietavirus*, *Rockefellervirus*, *Rosenblumvirus*, *Sciurinavirus*, *Sepunavirus*, *Sextaecvirus*, *Silviavirus*, *Triavirus*, *Twortvirus* and *Zhangqianvirus* (Table S1). Most of these phages belong to the class *Caudoviricetes*, and those with an assigned family are predominantly members of *Herelleviridae*. This family, recognized by the International Committee on Taxonomy of Viruses (ICTV), comprises phages that typically infect *Firmicutes* and was established based on phylogenetic analyses of conserved core genes. Species within the *Herelleviridae* family typically share more than 95% nucleotide identity across their genomes. These phages have large, linear double-stranded DNA genomes (typically 125-170 kbp) and exhibit tailed virions with icosahedral heads (85-100 nm) and contractile tails (130-185 nm) (Figure 1A). They encode conserved structural and functional proteins such as the major capsid protein, tail sheath protein, baseplate J-like protein, tail tube protein, portal protein, prohead protease, and the terminase large subunit (Figure 1B) (Barylski et al., 2020).

Although less common, phages from other families have also been reported infecting *Staphylococcus*. One example is the lytic phage vB_SauP_ASUmrsa123, isolated from raw sewage in Egypt and classified within the family *Rountreeviridae* (El-Tawab et al., 2024). This phage differs from *Herelleviridae* representatives by possessing a significantly shorter tail (~14 nm) and a more compact genome of only 17,155 bp. In addition, its genome encodes a distinct capsid “Arstotzka” protein not commonly found in *Herelleviridae*.

**Figure 2 -:**
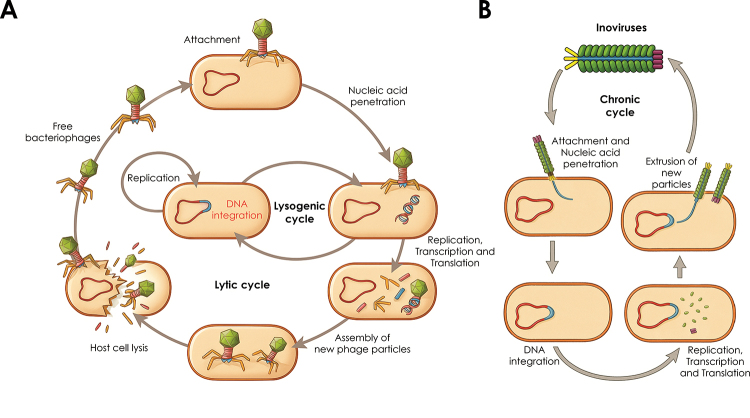
Main life cycles of bacteriophages. (A) Lytic and lysogenic cycles. (B) Chronic cycle, characteristic of inoviruses.

The phages from both *Herelleviridae* and *Rountreeviridae* typically replicate via one of two distinct reproductive strategies: the lytic or the lysogenic cycle (Figure 2A). In the lytic cycle, the phage adsorbs to the bacterial surface, injects its genetic material, and redirects the host’s machinery to produce new phage particles. This culminates in host cell lysis and the release of progeny virions. In contrast, during the lysogenic cycle, the phage genome integrates into the host chromosome and remains dormant as a prophage. This latent form replicates passively with the host until certain stimuli induce the switch to a lytic cycle, resuming active replication and eventual lysis. In addition to these two main strategies, a third, less understood state known as pseudolysogeny can occur, in which the phage genome persists in the host without integration or immediate replication, often under nutrient-limited or stressful conditions (Venturini et al., 2022). It represents a transient carrier state rather than a stable lysogen.

It is also worth noting that members of the family *Inoviridae* follow a chronic replication cycle (Figure 2B), where phages are continuously assembled and extruded from the host cell without lysis. Structurally, inoviruses are characterized by long, flexible, filamentous virions composed of a helical capsid surrounding a single-stranded DNA genome (Burckhardt and Tropini, 2023). However, such phages are not known to infect *Staphylococcus* species. They primarily infect Gram-negative bacteria, particularly members of the families *Enterobacteriaceae*, *Vibrionaceae*, *Pseudomonadaceae*, and *Xanthomonadaceae* (Knezevic and Adriaenssens, 2021).

**Figure 3 -:**
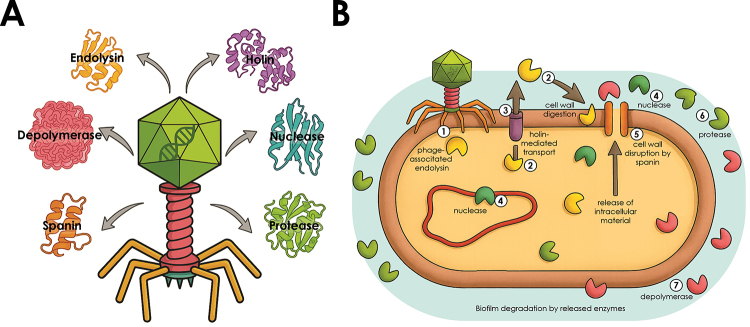
Bioproducts derived from the bacteriophage replication cycle. (A) Key enzymes and proteins produced during different stages of the phage replication cycle. (B) Sites of action of these bioproducts in the bacterial host and their effects on biofilm structures. Endolysins may be structurally associated with the phage particle (1) or secreted during the late stages of phage assembly (2). These enzymes are transported to the bacterial cell exterior by holins (3), which facilitate cell wall degradation. Nucleases digest bacterial DNA (4), which may also be released extracellularly through the action of endolysins or spanins (5), leading to cell envelope rupture. Additional enzymes, such as proteases (6) and depolymerases (7), can also be released during this process, contributing to the disruption of bacterial biofilms.

During the replication and lysis stages of the lytic cycle, bacteriophages synthesize and coordinate the activity of various enzymes and structural proteins that facilitate host subversion, lysis, and dissemination of viral progeny (Figure 3A) (Guliy and Evstigneeva, 2025). A subset of endolysins - peptidoglycan-degrading enzymes - are associated with phage tail fibers or baseplates and act early during infection by degrading localized regions of the bacterial cell wall, thereby facilitating genome injection (Abdelrahman et al., 2021). In contrast, canonical endolysins accumulate in the host cytoplasm and act at the terminal stage of the lytic cycle, in coordination with holins. These small membrane proteins form timed pores in the cytoplasmic membrane, enabling the cytoplasmic endolysins to access and degrade the peptidoglycan, leading to cell lysis. In some phages, spanins further assist in membrane disruption, particularly in Gram-negative bacteria (Samir, 2024). Nucleases and proteases produced during replication contribute to the degradation of host nucleic acids and proteins, respectively, favoring phage propagation. Depolymerases, often associated with the virion structure, degrade extracellular polysaccharides and facilitate access to bacterial surfaces, especially in the context of biofilm-forming strains (Figure 3B) (Guo et al., 2023).

Notably, some of these enzymes - including endolysins, nucleases, proteases, and depolymerases - can be released into the extracellular environment, where they may contribute to the disruption of *Staphylococcus* biofilms (Figure 3B) (Ferriol-González and Domingo-Calap, 2020). These biofilms exhibit considerable compositional heterogeneity among species and strains, comprising polysaccharides, structural proteins, and extracellular nucleic acids such as DNA and, occasionally, RNA (Rossi et al., 2024).

## Genomic architecture of *Staphylococcus* phages: Evolutionary and therapeutic implications

**Figure 4 -:**
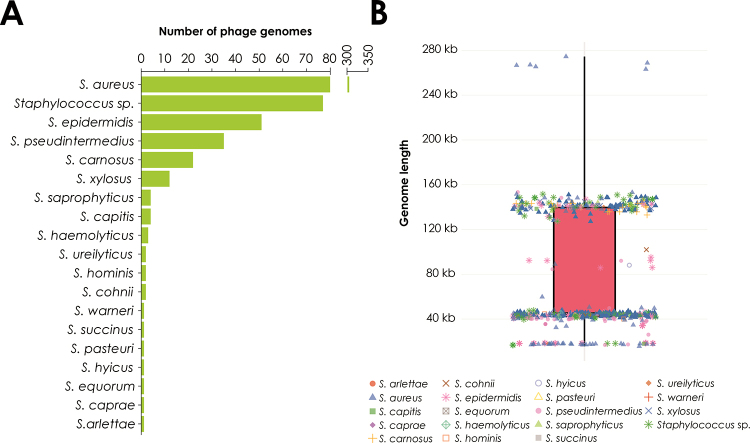
General genomic features of bacteriophages isolated from *Staphylococcus* hosts. (A) Main bacterial hosts from which *Staphylococcus* phages have been isolated, based on sequences publicly available in GenBank. (B) Average genome size of these bacteriophages. The genomes analyzed (detailed in Table S1) were retrieved in May 2025 and comprise a total of 524 sequences.

Most of the staphylococcal phages genome sequences available to date are derived from phages infecting *Staphylococcus aureus* or *Staphylococcus* strains identified only at the genus level, followed by phages targeting other clinically relevant species, such as *S. epidermidis*, *S. pseudintermedius*, among others (Figure 4A).

A comprehensive analysis of all complete genomes of phages infecting *Staphylococcus* spp., conducted in this study, reveals that these viruses have an average genome size of 77.2 ± 52.0 kb, being primarily grouped into two major categories: a predominant group averaging 141.4 ± 4.8 kb, and a smaller-genome group averaging 43.3 ± 2.6 kb (Figure 4B). Phages in the former group are typically lytic (Jiang et al., 2021; Zhou et al., 2023), while those in the latter are generally temperate (Barasuol et al., 2022; Viana et al., 2025). While these two size classes dominate the current dataset, additional groups were identified: one comprising phages with particularly small genomes averaging 17.9 ± 1.4 kb, another with intermediate genomes around 90.9 ± 4.8 kb, and a set of rare phages reaching up to 267.6 ± 3.5 kb (Figure 4B). The latter represents jumbo phages, a term used to describe phages with genomes typically larger than 200 kb, often encoding a wide array of accessory genes and complex replication machinery. 

Jumbo phages constitute a particularly intriguing subset due to their atypical genetic architecture and potential for biotechnological applications. These phages often encode their own multi-subunit RNA polymerases, reverse transcriptases, and other complex replication-related proteins, granting them a degree of independence from host machinery and enhancing their potential to overcome bacterial defense systems (Yuan and Gao 2017). In the case of *Staphylococcus* spp., only a few jumbo phages have been fully characterized to date, including PALS2, Madawaska, MarsHill, and DC4 (Lee et al., 2021; Korn et al., 2021). These viruses show a conserved repertoire of genes associated with lytic activity, such as endolysins, depolymerases, and tail-associated enzymes, and are often resistant to extreme pH conditions, although they display limited thermal stability. Notably, the genome of the phage DC4 (263,185 bp) is among the largest described for *Staphylococcus*, and despite its size, it encodes only a single tRNA gene, suggesting distinct selective pressures shaping its evolution (Silva et al. 2023). The phylogenomic clustering of these jumbo phages suggests a close relationship with *Bacillus*-infecting viruses, pointing to potential host-range plasticity and gene flow between phages infecting different genera.

**Figure 5 -:**
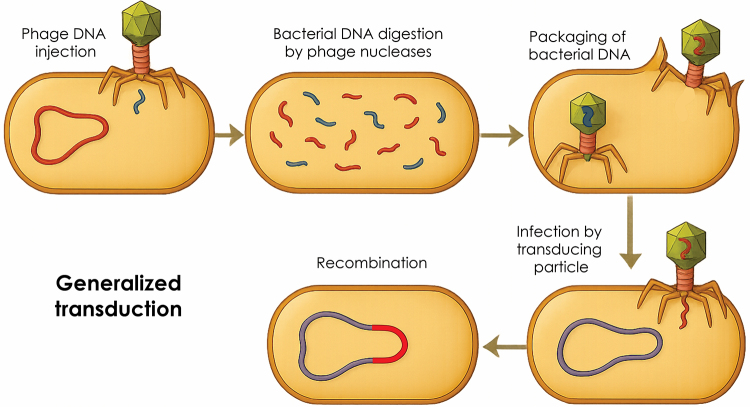
Horizontal gene transfer mediated by bacteriophages through generalized transduction. Illustration of how bacterial DNA fragments are mistakenly packaged into phage capsids and transferred to new host cells.

Despite the promising applicability of phages as antimicrobial agents, one of the potential consequences of their infection and life cycle is transduction. Transduction is a form of horizontal gene transfer mediated by bacteriophages, whereby bacterial DNA is inadvertently packaged into phage capsids and delivered to a new host during subsequent infections (Figure 5). This process can be generalized, when random fragments of host DNA are transferred, or specialized, when only specific regions adjacent to the prophage integration site are mobilized (Schneider, 2021). In *Staphylococcus*, transduction is a particularly relevant concern, as it contributes to the dissemination of antibiotic resistance genes, virulence factors, and mobile genetic elements, especially among closely related strains. It is widely perceived as the main mechanism of horizontal gene transfer in this genus (Haaber et al., 2017).

**Figure 6 -:**
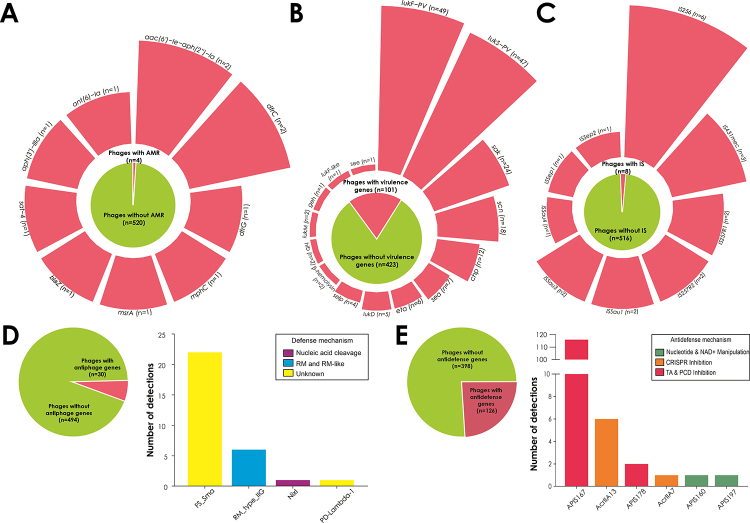
Gene composition of *Staphylococcus* bacteriophages, based on an in-depth analysis of 524 genomes available in GenBank (Table S1). This figure presents a quantitative analysis of the presence of (A) antimicrobial resistance (AMR) genes, identified using ResFinder (Bortolaia et al., 2020) and the CARD (McArthur et al., 2013) databases; (B) virulence genes, identified using VFDB (Liu et al., 2022); (C) insertion sequences (IS), identified using TnCentral (Ross et al., 2021); (D) bacterial defense systems, identified with DefenseFinder and PADLOC (Tesson et al., 2022; Payne et al., 2022); and (E) anti-defense systems that counteract host immune responses, identified using dbAPIS (Yan et al., 2024).

Given this context, one of the major concerns surrounding the therapeutic use of bacteriophages is the potential for viral infection to coincide with the transfer of resistance genes. However, intriguingly, most *Staphylococcus* phages do not carry resistance determinants. In fact, only approximately 0.8% of all sequenced staphylococcal phage genomes contain any identifiable resistance gene (Figure 6A). This finding challenges earlier - and above mentioned - assumptions that phages would serve as major drivers of antimicrobial resistance spread among staphylococci (Haaber et al., 2017).

The scenario is somewhat more expressive when it comes to virulence genes. Approximately 19.3% of *Staphylococcus* phage genomes harbor at least one known virulence factor (Figure 6B), particularly the *lukS*-PV and *lukF*-PV pair of genes, found in close to 9% of the phages, which encode the prototoxins of Panton-Valentine leukocidin (PVL). PVL is a bicomponent pore-forming cytotoxin associated with leukocyte destruction and increased tissue necrosis, especially in community-acquired *S. aureus* infections (Okolie et al., 2013). Another recurrent virulence gene is *sak* (found in 4.6% of the phages), encoding staphylokinase, a phage-associated enzyme that activates plasminogen into plasmin, facilitating immune evasion and tissue dissemination by degrading fibrin clots (Wang et al., 2022). One alternative to ensure the safety of such phages application in therapy is the use of genetic engineering strategies (Eghbalpoor et al., 2024). Additionally, *Staphylococcus* phages carry relatively few insertion sequences (IS), which could otherwise facilitate integration into the bacterial genome or other mobile genetic elements (Figure 6C).

The dynamics of viral infection and associated gene transfer are largely influenced by the presence of bacterial defense systems against foreign genetic material, as well as phage-encoded counter-defense mechanisms. The primary bacterial defense systems include CRISPR-Cas, restriction-modification (RM) systems, and abortive infection (Abi) systems, which together form the so-called bacterial defensome or pan-immune system (Bernheim and Sorek, 2020; Silva and Rossi, 2025). CRISPR-Cas systems provide adaptive immunity by incorporating short sequences from invading DNA into CRISPR arrays and using them to guide nuclease activity during subsequent infections (Rossi *et al.,* 2017). RM systems rely on restriction enzymes to cleave unmodified foreign DNA while protecting host DNA through specific methylation patterns (Enikeeva et al., 2010). Abi systems function as a form of altruistic self-sacrifice, triggering cell death upon detection of phage infection to prevent viral propagation (Lopatina et al., 2020).

In response, bacteriophages may encode their own anti-defense strategies to ensure successful infection. Among these, anti-CRISPR proteins inhibit components of the CRISPR-Cas machinery, blocking the cleavage of phage DNA (Pawluk et al., 2018). Additionally, some phages express proteins that interfere with programmed cell death pathways, preserving the host long enough to complete their replication cycle (Pinilla-Redondo et al., 2020).

Approximately 5.7% of *Staphylococcus* phages carry their own anti-phage defense systems, enabling them to outcompete other co-infecting phages targeting the same host. Among the most common are RM systems, which cleave the DNA of competitor phages (Figure 6D). Furthermore, around 24% of these phages harbor anti-defense systems, particularly toxin-antitoxin modules, programmed cell death inhibitors, and anti-CRISPR elements, enhancing their infectivity and survival in hostile bacterial environments (Figure 6E).

Together, these findings reveal the remarkable genomic diversity and functional plasticity of *Staphylococcus* phages. Their genomes not only reflect evolutionary adaptations to complex host defense systems but also offer valuable insights into their therapeutic potential and safety. A comprehensive understanding of these genomic traits is essential for harnessing phages as reliable tools against antibiotic-resistant staphylococcal infections.

## State-of-the-art of multi-branched preclinical studies with staphylococcal phages

Studies involving *Staphylococcus* phages date back to the very discovery of bacteriophages. It is now believed that the lytic agents observed by Frederick Twort in 1915 were in fact bacteriophages infecting *Staphylococcus* species that contaminated a vaccinia virus culture (Aswani and Shukla, 2021).

With the recent resurgence of interest in phage therapy due to the escalating threat of antimicrobial resistance, there has been a growing number of preclinical studies characterizing phages infecting *Staphylococcus* spp. These investigations aim to evaluate the therapeutic potential of phages through *in vitro* and *in vivo* models, shedding light on their host specificity, stability, lytic activity, and synergistic effects with conventional antimicrobials. Although much of this research remains within academic and research institutions, a growing number of biotechnology companies have emerged, aiming to translate these findings into practical clinical applications and viable business models that will be further explored in the final section of this review.

Among the most promising lines of investigation are those centered on broad-spectrum lytic phages. In a pivotal study, Göller et al. (2021) isolated 94 novel phages from wastewater and tested them against 117 strains belonging to 29 *Staphylococcus* species. Most of these phages displayed a broad host range, often crossing the boundaries between coagulase-positive and coagulase-negative staphylococci. Remarkably, only four phages were restricted to a single species, while the others infected a diverse array of hosts, including antibiotic-resistant and multi-drug-resistant strains. Many of these phages could lyse strains from distinct ecological origins (human, animal, and environmental), highlighting their potential for applications in One Health contexts.

In another study, Moller et al. (2021) investigated the genetic basis of bacteriophage susceptibility in *Staphylococcus aureus* on a species-wide scale. The authors developed a high-throughput assay to assess the host range of eight phages of different species across 259 diverse *S. aureus* strains. Their results showed substantial variation in phage sensitivity among strains, with host range strongly associated with clonal complex but not with methicillin resistance status. Using genome-wide association studies (GWAS), the authors identified several known and novel genes associated with phage resistance. Among the newly implicated genes were *trpA*, *phoR*, *isdB*, *sodM*, *fmtC* (also known as *mprF*), and *relA*. These findings were experimentally validated through transposon mutagenesis and complementation assays, confirming their role in influencing susceptibility to phage infection.

Another study demonstrating the broad host range of *Staphylococcus* phages, described by Kolenda et al. (2022), reported the isolation and characterization of three novel *Silviavirus* phages capable of lysing 82% of a diverse collection of 150 *S. aureus* clinical strains, including both methicillin-resistant and -susceptible lineages. Beyond evaluating the therapeutic potential of these phages, the authors proposed a quality-by-design strategy to optimize phage amplification for clinical use. This included the careful selection of bacterial production hosts that were free of major virulence genes and prophages, thereby minimizing the risk of introducing undesired genetic elements into phage preparations. To further enhance yield and scalability, they tested different culture media and multiplicities of infection (MOIs), ultimately identifying Superior Broth™ (AthenaES, USA) as the most effective condition, enabling rapid amplification and achieving phage titers of up to 10¹¹ PFU/mL within just 3-4 hours.

While lytic phages are typically favored for therapeutic purposes due to their immediate bactericidal activity and lower risk of horizontal gene transfer, recent research has also highlighted the promising potential of temperate phages. Although genome integration is a hallmark of temperate phages, it does not necessarily exclude them from consideration as biotechnological assets. In fact, this feature can be addressed or even exploited depending on the application - for instance, by utilizing phage-derived enzymes or cell-free lysates.

For instance, Zhang et al. (2022) characterized the temperate phage vB_SauS_JS02, isolated from livestock wastewater, which showed strong lytic activity against multidrug-resistant *S. aureus*. Despite encoding lysogeny-related genes, JS02 exhibited broad host range, high burst size, and significant inhibition of planktonic growth (80-90%) and biofilm formation (up to 70%), even outperforming ceftazidime in several assays.

Viana et al. (2025) isolated the temperate pGLS phage, which, despite its narrow lytic activity against *Mammaliicoccus sciuri*, showed broad antibiofilm effects against various multidrug-resistant *Staphylococcaceae*. This was mainly attributed to enzymes encoded by the phage, such as endolysins and nucleases, highlighting its potential as a biotechnological tool for biofilm control.

Feng et al. (2021) described JD419, another temperate phage with a distinct elongated capsid, which lysed 44% of 138 tested *S. aureus* clinical isolates, including multidrug-resistant strains. JD419 showed rapid adsorption, a burst size of ~33 PFU/cell, and environmental resilience within pH 6-8 and up to 40 °C. Despite possessing integrase and repressor genes, its genome lacked virulence and antibiotic resistance determinants, suggesting it could serve as a scaffold for engineering strictly lytic therapeutic variants.

Another promising direction in the study of *Staphylococcus* phages is the use of phage-derived bioproducts, particularly endolysins. These enzymes exhibit high catalytic specificity and potent bactericidal activity, due to the accessibility of their thick peptidoglycan layer (Gutiérrez et al., 2018). Endolysins typically possess modular architectures with distinct enzymatic activity domains (CHAP, amidase, glucosaminidase, etc.) and cell wall-binding domains (e.g., SH3b), allowing both targeted bacterial killing and opportunities for bioengineering (Golban et al., 2025). Studies have shown that engineered or chimeric endolysins - obtained by domain shuffling or fusion with cell-penetrating peptides - can enhance stability, broaden host range, and even reach intracellular *S. aureus* reservoirs (Schmelcher et al., 2012; São-José, 2018).

Although endolysins are the most widely explored phage-derived products, other viral components have also gained attention for their therapeutic potential. One study by Olsen et al. (2018) investigated the synergistic action of a phage endolysin (LysK) and a polysaccharide depolymerase (DA7) against *Staphylococcus aureus* biofilms. While LysK targets and degrades the bacterial peptidoglycan, DA7 disrupts the biofilm matrix by degrading poly-N-acetylglucosamine (PNAG), a major polysaccharide component. Their combined use enhanced biofilm eradication under both static and dynamic conditions, showing that multi-enzyme strategies targeting both bacterial cells and the biofilm matrix can be more effective than individual enzymes alone.

In another recent study, Zheng et al. (2025) characterized Hol-4086, a holin protein encoded by a *Staphylococcus* phage, and demonstrated its strong bacteriolytic activity. This holin was shown to possess direct extracellular antibacterial activity, significantly reducing *S. aureus* biofilm formation, disrupting mature biofilms, and decreasing bacterial loads in infected mice.

Another promising and expanding field in phage-based antimicrobial research involves combining bacteriophages or their derivatives with other antimicrobial compounds to enhance therapeutic efficacy and mitigate the emergence of resistance. These synergistic approaches offer the advantage of reducing the required dose of each agent, decreasing cytotoxicity, and limiting bacterial escape mechanisms. Several studies have evaluated combinations of phages with conventional antibiotics (Jo et al., 2016). One example is the use of phage Sb-1 with oxacillin against MRSA, which significantly enhanced bacterial killing, even though oxacillin alone was ineffective against resistant strains (Simon et al., 2021). The synergistic effect was evident across different MRSA isolates and phage concentrations, with antagonism being a rare exception.

Beyond antibiotics, synergy has also been demonstrated between phages and surfactants. A study combining phage pSa-3 with the non-ionic surfactant Tween 20 showed enhanced biofilm degradation and bacterial clearance on the skin in a murine model (Kim et al., 2020). The surfactant increased phage adsorption and disaggregated *Staphylococcus aureus* clusters, improving the overall efficacy of the phage treatment without inducing inflammation.

In another line of research, phages have been combined with bacteriocins - antimicrobial peptides produced by bacteria. For instance, the combination of SAP84 phage and crude bacteriocin from *Lactococcus lactis* CJNU 3001 resulted in enhanced inhibition of *S. aureus* growth compared to either agent alone, suggesting a viable strategy for food safety and clinical applications (Kim et al., 2019).

Further, phage-derived enzymes such as endolysins have shown synergy with other compounds. Endolysin LysH5 combined with nisin was able to eradicate viable *S. aureus* in milk (García et al., 2010), and engineered hybrid lysins like SAL200 or ClyS have shown enhanced action when used alongside antibiotics like vancomycin and oxacillin, even reducing infection severity in animal models (Kim et al., 2018).

Lastly, essential oils - complex mixtures of volatile antimicrobial compounds - have been explored in combination with phages. Their multifaceted mode of action, targeting different bacterial structures simultaneously, makes them particularly promising partners (Ghosh et al., 2016). This complexity helps reduce the likelihood of resistance development and enhances the disruption of biofilms when used in conjunction with lytic phages.

Increasing attention is being given to studies focusing on developing phage cocktails. These combinations bring together different phages with complementary host specificities to overcome the narrow spectrum of individual phages. Traditionally, cocktails have been assembled based on available isolates in local collections, but recent efforts have aimed to make this process more systematic. One approach is to use association rule mining, a data analysis method originally used in marketing, to identify patterns of phage co-occurrence or exclusivity across bacterial strains (Lood et al., 2022). This helps select phages that target different groups of bacteria or use different receptors, increasing coverage and reducing the risk of resistance. As a result, phage cocktails are seen not only as a practical solution, but also as a strategic way to improve the effectiveness and sustainability of phage therapy.

Altogether, these studies underscore the potential of multi-pronged antimicrobial approaches combining phages or their bioproducts with diverse compounds, aiming not only for higher efficacy but also for more sustainable resistance control. 

## Clinical evidence for *Staphylococcus* phage therapy

Although phage therapy was largely discontinued in many parts of the world with the advent of antibiotics, it continued to be explored and applied in Eastern Europe and parts of Asia. In these regions, bacteriophage-based treatments were used successfully in clinical settings, particularly for infections that were unresponsive to available antibiotics. Unfortunately, a large portion of this early clinical experience remains underreported or difficult to access, largely due to language barriers, as many of the studies were published in Russian or French.

A detailed review by Abedon et al. (2011) recovered several of these historical cases, documenting phage applications against *Staphylococcus* clinical infections as early as 1921. These reports span a wide array of clinical conditions, including skin and soft tissue infections, surgical wound infections, osteomyelitis, and systemic diseases like bacteremia and pericarditis. The routes of administration varied depending on the infection site and severity, encompassing topical, oral, intravenous, and subcutaneous delivery. Despite the diversity in protocols, many of these early interventions resulted in symptom resolution or significant clinical improvement, reinforcing the therapeutic potential of phages when appropriately applied.

A broader synthesis of modern clinical trials is provided by Uchechukwu and Shonekan (2024), who reviewed both ongoing and completed studies targeting multidrug-resistant pathogens. Although many of these trials have focused on *Pseudomonas aeruginosa* and *Escherichia coli*, several included *Staphylococcus aureus*, particularly in complex infections such as diabetic foot ulcers and prosthetic joint infections. The review highlighted diverse routes of administration - including intravenous, topical, and intraoperative - and reported no serious adverse events. In most *S. aureus*-related cases, phage therapy yielded clinical improvement, even in patients unresponsive to antibiotics.

Another important milestone in this field is the study by Fabijan *et al.* (2020), which assessed the safety of a GMP-grade phage cocktail (AB-SA01) in 13 critically ill patients with severe *S. aureus* infections, including infective endocarditis and septic shock. Administered intravenously twice daily for 14 days, the therapy was well tolerated and resulted in clinical improvement in 62% of patients by day 14. No phage-related adverse effects or *in vivo* emergence of phage resistance were observed, making this one of the first systematic evaluations of phage safety in life-threatening *S. aureus* infections.

Expanding on this, Aslam et al. (2020) reported on the first 10 cases of intravenous phage therapy at the Center for Innovative Phage Applications and Therapeutics (IPATH) in the U.S. Among these, one involved a persistent *S. aureus* infection refractory to multiple antibiotic regimens. Administered under FDA-approved single-use IND protocols and combined with antibiotics, phage therapy led to clinical improvement in most patients and no phage-related adverse events.

A retrospective analysis by Green et al. (2023) described 12 cases of customized phage therapy for difficult-to-treat infections, including three involving *S. aureus*. Infections included sternal wound infections and left ventricular assist device (LVAD)-associated bacteremia. Phages were administered intravenously, with some patients also receiving intraoperative or topical doses. Two of the three *S. aureus* cases responded favorably, with no adverse events and observed phage-antibiotic synergy in most instances.

In another notable case, Van Nieuwenhuyse et al. (2021) reported the use of *in situ* phage therapy in a 13-year-old girl with a chronic polymicrobial bone allograft infection involving methicillin-sensitive *S. aureus*. After failing conventional antibiotic therapies, a targeted phage (ISP) was delivered via drainage catheter, leading to short-term microbiological clearance and marked clinical improvement. Although the infection later recurred - likely due to suboptimal antibiotic adherence and residual polymicrobial infection - the case illustrated both the potential and limitations of phage therapy in complex clinical scenarios.

Although clinical experience in humans is growing, animal models have also provided important clinical insights. Liu et al. (2021) conducted a systematic review of 20 animal studies involving phage therapy, including against *S. aureus*. Routes of administration ranged from intranasal to intraperitoneal and topical, with no major adverse events reported. Phages significantly reduced bacterial loads in models of sinusitis, bacteremia, and pneumonia. Minor immune responses such as cytokine elevation and anti-phage antibody formation were observed but did not correlate with toxicity. These findings support the safety of phage therapy in translational models.

Fortaleza et al. (2024) highlight the urgent need for more rigorous and standardized clinical trials involving phage therapy, particularly targeting MRSA. Despite promising results from *in vitro*, animal, and compassionate-use human studies, logistical and regulatory barriers continue to limit its broader implementation. Still, cumulative evidence supports phage therapy as a promising and safe adjunct to traditional antibiotics.

Taken together, the growing body of clinical case reports, compassionate-use treatments, and early-phase trials demonstrate that phage therapy has moved well beyond theoretical application. While standardized protocols, regulatory clarity, and large-scale clinical trials remain necessary, the progress achieved so far indicates strong potential for phages to re-emerge as practical tools in modern infectious disease management.

## Business models driving the translation of phage innovation

Once mostly confined to academic research, phage therapy is now advancing industrially through innovations in synthetic biology, genome sequencing, and artificial intelligence, which enable targeted phage-host matching and therapeutic optimization (Kortright et al., 2019; Segundo-Arizmendi et al., 2025). Multiple companies, at different stages of development, are now entering the phage therapy market, each adopting distinct business models tailored to specific challenges and sectors (examples are presented in Chart S1).

Supportive regulatory frameworks, such as adaptive clinical trials (which allow protocol adjustments as results emerge) and named-patient programs (which permit the use of experimental treatments for individual patients in need), endorsed by agencies like the U.S. Food and Drug Administration (FDA) and European Medicines Agency (EMA), have contributed to progress in translating phage therapies into clinical settings (Verbeken et al., 2014; Pirnay et al., 2015). In parallel, early investments in startups reveal growing institutional interest (Cui et al., 2024). Beyond human medicine, phage-based products are being applied to agriculture, veterinary care, and industry (Park et al., 2020; Liang et al., 2023; Choi et al., 2024), including uses such as antibiotic alternatives in livestock (Wernicki et al., 2017), prevention of damage to oil and gas pipelines caused by microbial activity (Zhang et al., 2025), and biofilm disruption in wastewater treatment (Jassim and Limoges, 2014; Mayorga-Ramos et al., 2024). These developments position phages as versatile biotechnological tools across multiple sectors (Abedon et al., 2011; Fernández et al., 2018). According to the Verified Marketing Reports, available at https://www.verifiedmarketreports.com/ (report ID 821468), the global market for phage therapy alone is projected to reach USD 5.2 billion by 2033, representing a fourfold increase from 2024, with steady annual growth expected throughout the decade. However, such projections vary substantially across sources, particularly depending on whether non-clinical applications such as industrial, veterinary, and food safety uses are included in the analysis.

This diversification is also shaping the commercial landscape. Companies tailor their development strategies to sector-specific needs, balancing innovation, regulation, and technical and regulatory feasibility. A common path involves isolating phages from environmental samples, evaluating their host range, sequencing genomes to ensure safety, and screening for undesirable genes. These steps are increasingly supported by high-throughput technologies and AI tools (Górski et al., 2020; Wang et al., 2023). Promising phages then undergo formulation, stability testing, and scale-up under Good Manufacturing Practice (GMP) standards, which remain a key bottleneck due to challenges in purification and endotoxin removal (Bretaudeau et al. 2020). Meanwhile, companies navigate regulatory approval and build manufacturing and delivery systems. To clarify the current business landscape, phage companies can be broadly grouped into four business models (Chart 1). While these categories provide a useful framework, several companies exhibit hybrid features or evolve across models over time:

**Chart 1 -:** Overview of business models supporting the translation of phage-based therapeutics.

Business Model	Core strategy	Advantages	Limitations
End-to-End Developers	Control all stages from discovery to GMP manufacturing and clinical trials.	High quality control, IP protection, regulatory alignment	High operational cost, complex regulatory demands, long timelines
Application-Focused Distributors	Develop ready-to-use products for sectors like agriculture and food safety.	Faster market entry, lower regulatory burden, sector-specific deployment	Regulatory heterogeneity, need for field efficacy data and long-term stability
Scalable and Regulated Access Models	Combine innovation and access through public-private partnerships, consortia and expanded access pathways.	Regulatory flexibility, clinical agility, responsiveness to urgent needs	Reliance on public funding, complexity in transitioning from access to approval
Magistral and On-Demand Therapy Models	Provide patient-specific phage therapies via local compounding in hospital or clinics.	High personalization, rapid bedside deployment, bypasses centralized drug approval	Low scalability, quality control variability, restricted to favorable legal frameworks

End-to-End Developers maintain full control over the product lifecycle - from discovery to GMP manufacturing and clinical trials. Some companies within this model have integrated CRISPR-based engineering or AI-driven phage-host matching tools into their platforms, while others rely primarily on empirical selection and conventional microbiological screening. These firms typically build proprietary platforms that combine phage engineering, their own regulatory management, and manufacturing infrastructures that meet pharmaceutical standards. Their approach gives them greater control over intellectual property, quality assurance, and data generation required for regulatory approval. Such integration is especially advantageous for addressing complex or resistant infections in clinical settings, where consistent manufacturing and rigorous safety standards are essential. Several developers have incorporated *Staphylococcus aureus*, including methicillin-resistant strains (MRSA), into their therapeutic pipelines, targeting indications such as bacteremia, diabetic foot infections, and surgical site complications, reflecting both clinical urgency and commercial opportunity in hospital-acquired infections. Many also engage in strategic partnerships or government-funded programs to support their clinical pipelines and reduce dependency on private capital, an approach that helps mitigate some of the financial risks associated with long development timelines. This is the case of companies such as *Armata Pharmaceuticals*; *BiomX | Phage therapy (which acquired Adaptive Phage Therapeutics in March 2024)*; *Intralytix, Inc.* and *Locus Biosciences* (Chart S1). The March 2024 merger folded Adaptive Phage Therapeutics’ PhageBank™ platform and rapid-access network into BiomX’s vertically integrated operations, further consolidating its end-to-end capabilities.

However, end-to-end models face substantial operational and financial challenges. The capital intensity of maintaining in-house capabilities across all stages, from discovery to multicenter clinical trials, is considerable. The need to comply with pharmaceutical-grade standards (such as GMP) increases both complexity and cost, particularly in ensuring phage purity, potency, and batch-to-batch consistency despite the biological variability inherent to phages. Moreover, the lack of dedicated regulatory frameworks for phage therapeutics in most jurisdictions complicates classification, trial design, and approval timelines, especially for genetically modified or adaptive formulations (Pirnay et al., 2011; Verbeken and Pirnay, 2022). Public perception and biosafety concerns regarding engineered phages add additional layers of complexity (Rohde et al., 2018). Some end-to-end developers have extended their platforms to adjacent sectors, such as food safety or chronic inflammatory conditions, using modular development strategies to broaden application while leveraging existing infrastructure (Ilyina et al., 2019). Although such diversification can enhance resilience, it may also dilute clinical focus and complicate regulatory alignment. In summary, while this model offers strong advantages in control and scalability, it demands sustained investment, deep technical expertise, and long-term strategic planning.

Application-Focused Distributors prioritize the formulation, regulatory registration, and distribution of phage-based products targeted to specific industrial sectors such as agriculture, aquaculture, veterinary medicine, and food safety. Operating predominantly under a business-to-business (B2B) model, these firms emphasize practical deployment, aligning their technologies with end-user needs and regional regulations. Their platforms are typically geared toward high-volume production and field-ready formulations, with development pipelines optimized for ease of integration into existing agricultural or food processing systems. These characteristics enable faster market entry compared to therapeutic applications in human medicine, making this model particularly attractive for companies aiming to commercialize phages under less burdensome regulatory frameworks. This is the case of companies such as *Microbiotec*; *Phagelux AgriHealth* and *Proteon Pharmaceuticals* (Table S1).

Many of these distributors leverage proprietary discovery platforms and in-house production capabilities, yet focus less on clinical development and more on applied efficacy in non-human settings. Several have introduced phage-based formulations aimed at controlling pathogens relevant to livestock, aquaculture, and post-harvest contamination, sometimes including *Staphylococcus* species as part of their broader antimicrobial spectrum. Examples include products for mastitis control in dairy cattle, phage-based hygiene solutions for skin infections in animals, or environmental biocontrol agents that reduce zoonotic bacterial transmission. While this targeted operational model facilitates earlier commercialization, it is not without challenges. Regulatory heterogeneity across jurisdictions, particularly regarding environmental release and biosafety, complicates product registration and market scalability. Moreover, companies must demonstrate not only field-level efficacy and long-term stability but also maintain cost-effectiveness to remain competitive in price-sensitive sectors (Jones et al., 2012; Buttimer et al., 2017). As these markets evolve, future success will likely depend on securing robust distribution networks, advancing automation in production, and maintaining agility to adjust to shifting local and international regulations.

Scalable and Regulated Access Models operate between the experimental and the commercial, aiming to deliver therapeutic phages through flexible yet compliant pathways. Unlike fully integrated pharmaceutical pipelines or industrial-scale distributors, these models focus on enabling real-world patient access without waiting for full drug approvals. This is often achieved through public-private partnerships, expanded access programs, or clinical consortia that align with national or institutional health needs. This model is particularly valuable when time-sensitive access to treatment is needed, such as in the case of multidrug-resistant infections, yet traditional pharmaceutical development would take years. Rather than manufacturing patient-specific formulations, these companies typically develop scalable phage libraries or selection platforms and integrate with diagnostic tools to guide treatment with robust regulatory engagement and funding from governmental or institutional sources. This is the case of companies such as *Eligo | In vivo gene editing of the microbiome*; *Micreos* and *Phaxiam* (Chart S1). Although traditionally classified under regulated access models, Micreos has increasingly focused on phage-derived enzymes, such as endolysins, rather than whole-phage therapeutics. Its commercial pipeline, including the topical product Gladskin, targets inflammatory skin conditions and exemplifies how non-viable phage components can be integrated into consumer-oriented biomedical platforms.

Several of these companies focus on infections with high unmet clinical demand, such as chronic osteomyelitis, prosthetic joint infections, and multidrug-resistant *Staphylococcus aureus*. While some platforms are oriented toward hospital-based deployment under compassionate use or individualized therapy frameworks, others support precision-targeted phage selection through diagnostic-guided matching. These hybrid strategies enable faster access for critical indications while continuing to build long-term value through clinical trials and scalable manufacturing. Their flexibility, however, comes with trade-offs: navigating multiple regulatory pathways simultaneously can strain operational coherence, and reliance on public-sector funding raises concerns about financial sustainability. Furthermore, the clinical rigor required to convert expanded access into formal approval remains a major bottleneck (Pirnay et al., 2011; Ilyina et al., 2019). Nevertheless, the combination of scientific adaptability and institutional alignment positions these ventures to pioneer innovative phage deployment models that bridge the gap between lab discovery and patient care.

Magistral and On-Demand Therapy Models are built around personalized care and diagnostic-driven phage matching. Unlike companies pursuing regulatory approval and large-scale production, these organizations compound phage preparations directly for individual patients, often within hospital pharmacies or specialized clinics. The term ‘magistral’ refers to legal frameworks that allow clinicians or pharmacists to prepare custom treatments without formal drug registration. These models are particularly relevant in contexts where conventional pharmaceutical pathways are impractical, whether due to economic constraints, niche indications, or the urgency of addressing resistant infections. Rather than pursuing standardized commercialization and global regulatory filings, these institutions emphasize agility, diagnostic-driven personalization, and integration with local clinical infrastructure. Their operations typically revolve around patient-specific diagnosis and rapid adaptation of phage preparations, leveraging permissive regulatory frameworks to enable timely bedside deployment. This approach is common in countries like Georgia, where institutions such as the historical *Eliava Institute*, active in phage therapy since 1923, prepare tailored phage cocktails based on a patient’s specific bacterial isolate. Beyond its historical role, the institute currently operates as a hybrid public-private center encompassing phage production, clinical services, and magistral compounding. It collaborates with international partners to provide personalized phage therapy, particularly under permissive national regulations or compassionate use schemes, despite the absence of global GMP accreditation (Kutateladze and Adamia, 2008)*.*

One advantage of this model is its ability to translate scientific capacity into clinical care without the delay of centralized drug approvals. In some cases, this includes tailored phage cocktails for infections caused by *Staphylococcus aureus*, including multidrug-resistant strains. Diagnostic services and phage susceptibility testing are core components, allowing for rapid formulation of treatments based on each patient’s unique bacterial profile. However, the flexibility of these frameworks comes with operational and regulatory trade-offs. Ensuring product consistency, quality control, and reproducibility across individualized preparations remains a major challenge. In addition, such models often depend on specific national contexts that permit magistral use, making international scalability difficult. Despite these limitations, these institutions demonstrate the critical role of alternative delivery models in sustaining access to phage therapy, especially where conventional pharmaceutical development is too slow or financially unviable (Pirnay et al., 2011; Ilyina et al., 2019).

Altogether, the emergence of diverse business models in phage therapy reflects an evolving therapeutic landscape still in search of alignment between technological potential, clinical validation, and regulatory feasibility. These divergent paths represent not a fragmentation, but a strategic diversification of the phage innovation ecosystem, where each model responds to the specific constraints and opportunities of its use case. Rather than being mutually exclusive, these approaches function as complementary building blocks that together define a complex and dynamic field. Their coexistence suggests that the future of phage therapy will depend not only on bold scientific advances, but also on the development of adaptive business models and enabling infrastructures capable of delivering safe, effective, and equitable solutions. Shared challenges such as GMP-compliant manufacturing, regulatory harmonization, the limited predictability of phage-host interactions, and intellectual property protections must be addressed collaboratively across disciplines and sectors (Abedon et al., 2011; Buttimer et al., 2017; Yang et al., 2023).

## Conclusions

The resurgence of interest in staphylococcal phages is no longer a peripheral scientific curiosity - it has become a central theme in the broader effort to confront the antimicrobial resistance crisis. From foundational discoveries to current preclinical and clinical developments, the growing body of evidence supports bacteriophages not only as therapeutic agents but also as powerful tools to understand bacterial evolution, defense systems, and gene transfer. Their versatility extends across medical, veterinary, agricultural, and industrial applications, while advances in synthetic biology and regulatory innovation continue to reshape their translational potential.

In this broader context, bacteriophage innovation serves not merely as a technological frontier, but as a test case for the responsiveness and adaptability of global health and biotechnology systems. As conventional antimicrobials approach the limits of their efficacy, phages offer a biologically precise, evolutionarily robust, and ecologically aligned alternative. Realizing this potential will require strategic alignment among policymakers, regulators, investors, and scientists-not only to validate phage-based interventions, but to ensure they are translated into viable, scalable, and socially impactful applications. Bacteriophage-based technologies, long overlooked, may soon prove indispensable, not just as therapeutic agents, but as a cornerstone of the next generation of antimicrobial solutions.

## Supplementary material

The following online material is available for this article:

Table S1 - Genomes of bacteriophages infecting *Staphylococcus* available from Genbank, used in this work.

Chart S1 - Examples of companies working on the development of commercial phage therapy.

## Data Availability

All data are included in the manuscript.
