# Supplementary material for: Staphylococcal phages as agents of evolution and innovation: From gene flow to next-generation therapeutics
Source: Genet Mol Biol. 2026 Feb 9;49(Suppl 1):e20250131. doi: 10.1590/1678-4685-GMB-2025-0131 (PMC12895238; doi:10.1590/1678-4685-GMB-2025-0131)
Supplement: Table S1 - [file 1415-4757-GMB-49-s1-e20250131-s1.pdf]

Genetics and Molecular Biology
DOI: https://doi.org/10.1590/1678-4685-GMB-2025-0131

Supplementary Material to "Staphylococcal phages as agents of evolution and innovation: From gene flow to next-generation therapeutics"

Table S1 - Genomes of bacteriophages infecting Staphylococcus available from Genbank, used in this work.

Accession	Organism_Name	Country	Length (bp)	Host	Organization	Release_Date	Species	Genus	Family or Class
OP297179.1	Staphylococcus phage vB_ScaM-V1SC05	France	18001	*Staphylococcus capitis*	Hospices Civils de Lyon	08/10/2022	Staphylococcus phage vB_ScaM-V1SC05	Andhravirus	Rountreeviridae
MZ152915.1	Staphylococcus phage SeAlphi	Israel	18292	*Staphylococcus epidermidis*	Hebrew University, Institute of Dental Sciences and School of Dental Medicine	20/07/2021	Staphylococcus phage SeAlphi	Andhravirus	Rountreeviridae
KY471386.1	Staphylococcus phage St 134	Russia	18275	*Staphylococcus epidermidis*	Institute of Chemical Biology and Fundamental Medicine SB RAS, Laboratory of Molecular Microbiology	01/03/2017	Andhravirus St134	Andhravirus	Rountreeviridae
OK574338.1	Staphylococcus phage vB_SurP-PSU3	South Korea	18146	*Staphylococcus ureilyticus*	Korea Research Institute of Bioscience, Institution Disease Research Center	24/06/2022	Staphylococcus phage vB_SurP-PSU3	Andhravirus	Rountreeviridae
MT596500.1	Staphylococcus phage BESEP3	Switzerland	18271	*Staphylococcus epidermidis*	Inselspital, Intensive Care Medicine / DBMR	28/06/2021	Andhravirus besep3	Andhravirus	Rountreeviridae
KY442063.1	Staphylococcus phage Andhra	USA	18546	*Staphylococcus epidermidis*	University of Alabama, Biological Sciences	06/03/2017	Andhravirus andhra	Andhravirus	Rountreeviridae
MH972263.1	Staphylococcus phage JBug18	USA	18547	*Staphylococcus epidermidis*	University of Alabama, Biological Sciences	04/12/2018	Andhravirus andhra	Andhravirus	Rountreeviridae
MH972261.1	Staphylococcus phage Pike	USA	18376	*Staphylococcus epidermidis*	University of Alabama, Biological Sciences	04/12/2018	Andhravirus andhra	Andhravirus	Rountreeviridae
MH972262.1	Staphylococcus phage Pontiff	USA	18364	*Staphylococcus epidermidis*	University of Alabama, Biological Sciences	04/12/2018	Andhravirus andhra	Andhravirus	Rountreeviridae
MH028956.1	Staphylococcus phage phiSA_BS2	China	149229	*Staphylococcus aureus*	Fudan University, Department of Microbiology, School of Life Science	03/04/2018	Baoshanvirus BS2	Baoshanvirus	Herelleviridae
MH078572.1	Staphylococcus phage phiSA_BS1	China	142978	*Staphylococcus sp.*	Fudan University, Department of Microbiology,	26/03/2018	Baoshanvirus BS1	Baoshanvirus	Herelleviridae

Accession	Organism_Name	Country	Length (bp)	Host	Organization	Release_Date	Species	Genus	Family or Class
					School of Life Science				
LC557520.1	Staphylococcus phage vB_SsapH-Golestan101-M	NA	144081	*Staphylococcus saprophyticus*	Laboratory Sciences Research Center; Shast colah, Gorgan, Golestan 4934174515	26/06/2020	Staphylococcus phage vB_SsapH-Golestan101-M	Baoshanvirus	Herelleviridae
HE614281.1	Staphylococcus phage SpaA1	Antarctica	42784	*Staphylococcus pasteuri*	Information and Computational Sciences, James Hutton Institute, Invergowrie, Dundee, DD2 5DA	23/07/2012	Beceayunavirus BceA1	Beceayunavirus	Caudoviricetes
MG029510.1	Staphylococcus phage phiSa2wa_st22	Australia	38576	*Staphylococcus aureus*	Curtin University, School of Biomedical Sciences	10/01/2018	Biseptimavirus st22	Biseptimavirus	Caudoviricetes
MG029512.1	Staphylococcus phage phiSa2wa_st59	Australia	42133	*Staphylococcus aureus*	Curtin University, School of Biomedical Sciences	10/01/2018	Staphylococcus phage phiSa2wa_st59	Biseptimavirus	Caudoviricetes
OP038320.1	Staphylococcus phage phiSa2wa-st5.6	Australia	44139	*Staphylococcus aureus*	Curtin University, School of Biomedical Sciences	02/10/2022	Staphylococcus phage phiSa2wa-st5.6	Biseptimavirus	Caudoviricetes
OP136135.1	Staphylococcus phage Sa2wa-st573	Australia	42403	*Staphylococcus aureus*	Curtin University, School of Biomedical Sciences	26/10/2022	Staphylococcus phage Sa2wa-st573	Biseptimavirus	Caudoviricetes
KT878766.1	Staphylococcus phage P1105	Germany	42282	*Staphylococcus aureus*	Federal Institute for Risk Assessment, Biological Safety	24/05/2017	Biseptimavirus P1105	Biseptimavirus	Caudoviricetes
KT809368.1	Staphylococcus phage P282	Germany	41960	*Staphylococcus aureus*	Federal Institute for Risk Assessment, Biological Safety	24/05/2017	Biseptimavirus P282	Biseptimavirus	Caudoviricetes
KT809369.1	Staphylococcus phage P630	Germany	40448	*Staphylococcus aureus*	Federal Institute for Risk Assessment, Biological Safety	24/05/2017	Biseptimavirus P630	Biseptimavirus	Caudoviricetes
GQ398772.1	Staphylococcus phage P954	India	40761	*Staphylococcus aureus*	Gangagen Biotechnologies Pvt Ltd, Research & Development	01/09/2009	Biseptimavirus P954	Biseptimavirus	Caudoviricetes
CP062447.1	Staphylococcus phage ECel-2020d	NA	43956	*Staphylococcus aureus*	University of central Florida, Burnett School of	07/03/2022	Biseptimavirus NM3	Biseptimavirus	Caudoviricetes

Accession	Organism_Name	Country	Length (bp)	Host	Organization	Release_Date	Species	Genus	Family or Class
					Biomedical Sciences				
CP062430.1	Staphylococcus phage ECel-2020i	NA	44872	*Staphylococcus aureus*	University of central Florida, Burnett School of Biomedical Sciences	07/03/2022	Staphylococcus phage ECel-2020i	Biseptimavirus	Caudoviricetes
KY653123.1	Staphylococcus phage IME1361_01	NA	43516	*Staphylococcus aureus*	Beijing Institute of Microbiology and Epidemiology, State Key Laboratory of Pathogen	06/05/2017	Biseptimavirus IME136101	Biseptimavirus	Caudoviricetes
KF831354.1	Staphylococcus phage phiBU01	NA	43748	*Staphylococcus aureus*	Rockefeller University, Laboratory of Bacterial Pathogenesis and Immunology	19/07/2014	Biseptimavirus BU01	Biseptimavirus	Caudoviricetes
KJ596420.1	Staphylococcus phage phiSa119	NA	42600	*Staphylococcus aureus*	Istituto Superiore di Sanita', Dept. of Infectious, Parasitic and Immune-mediated Diseases	23/06/2014	Biseptimavirus P1105	Biseptimavirus	Caudoviricetes
OP493558.1	Staphylococcus phage phiSa2-NARSA676	NA	41838	*Staphylococcus aureus*	Curtin University, School of Biomedical Sciences	19/10/2022	Staphylococcus phage phiSa2-NARSA676	Biseptimavirus	Caudoviricetes
KC595278.1	Staphylococcus phage StauST398-4	NA	42906	*Staphylococcus aureus*	Geneva University Hospitals, Genomic Research Laboratory	11/02/2014	Biseptimavirus StauST3984	Biseptimavirus	Caudoviricetes
AY508486.1	Staphylococcus phage 77	NA	41708	*Staphylococcus sp.*	PhageTech Inc.	11/01/2004	Biseptimavirus bv77	Biseptimavirus	Caudoviricetes
AP011955.1	Staphylococcus phage phi5967PVL	NA	42461	*Staphylococcus sp.*	Department of Bacteriology; Hongo 2-1-1, Bunkyo-ku, Tokyo 113-8421	06/10/2011	Biseptimavirus phi7247PVL	Biseptimavirus	Caudoviricetes
AP011956.1	Staphylococcus phage phi7247PVL	NA	42481	*Staphylococcus sp.*	Department of Bacteriology; Hongo 2-1-1, Bunkyo-ku, Tokyo 113-8421	06/10/2011	Biseptimavirus phi7247PVL	Biseptimavirus	Caudoviricetes

Accession	Organism_Name	Country	Length (bp)	Host	Organization	Release_Date	Species	Genus	Family or Class
DQ530361.1	Staphylococcus phage phiNM3	NA	44061	*Staphylococcus sp.*	University of Chicago, Microbiology	31/10/2006	Biseptimavirus NM3	Biseptimavirus	Caudoviricetes
MH384259.1	Staphylococcus phage SA1014ruMSSAST7	Russia	43504	*Staphylococcus aureus*	N.F. Gamaleya National Research Center for Epidemiology and Microbiology Ministry of Health of Russia, Laboratory of Molecular Bases of Pathogenicity	25/07/2018	Biseptimavirus SA1014ruMSSAST7	Biseptimavirus	Caudoviricetes
MH401416.1	Staphylococcus phage SA345ruMSSAST8	Russia	42735	*Staphylococcus aureus*	N.F. Gamaleya National Research Center for Epidemiology and Microbiology Ministry of Health of Russia, Laboratory of Molecular Bases of Pathogenicity	25/07/2018	Biseptimavirus SA345ruMSSAST8	Biseptimavirus	Caudoviricetes
MW924889.1	Staphylococcus phage vB_StaphS-IVBph354	Switzerland	41970	*Staphylococcus aureus*	University of Bern, Institute of Veterinary Bacteriology	04/05/2021	Biseptimavirus P282	Biseptimavirus	Caudoviricetes
KJ452292.1	Staphylococcus phage 23MRA	USA	43098	*Staphylococcus aureus*	UCSD, Pathology	13/07/2015	Biseptimavirus bv23MRA	Biseptimavirus	Caudoviricetes
KX827368.1	Staphylococcus phage SpT5	Denmark	39804	*Staphylococcus pseudintermedius*	University of Warwick, School of Life Sciences	23/11/2016	Coventryvirus SN8	Coventryvirus	Caudoviricetes
MK075006.1	Staphylococcus phage phiSP119-3	NA	44311	*Staphylococcus pseudintermedius*	The Rockefeller University, Laboratoy of Bacterial Pathogenesis and Immunology	12/04/2019	Staphylococcus phage phiSP119-3	Coventryvirus	Caudoviricetes
MF428480.1	Staphylococcus phage SN10	NA	39779	*Staphylococcus pseudintermedius*	University of Copenhagen, Department of Veterinary and Animal Sciences	19/08/2017	Coventryvirus SN8	Coventryvirus	Caudoviricetes
MF428479.1	Staphylococcus phage SN11	NA	40108	*Staphylococcus pseudintermedius*	University of Copenhagen, Department of	19/08/2017	Coventryvirus WIS42	Coventryvirus	Caudoviricetes

Accession	Organism_Name	Country	Length (bp)	Host	Organization	Release_Date	Species	Genus	Family or Class
					Veterinary and Animal Sciences				
MF428478.1	Staphylococcus phage SN13	NA	40462	*Staphylococcus pseudintermedius*	University of Copenhagen, Department of Veterinary and Animal Sciences	19/08/2017	Coventryvirus SN8	Coventryvirus	Caudoviricetes
MF428481.1	Staphylococcus phage SN8	NA	39803	*Staphylococcus pseudintermedius*	University of Copenhagen, Department of Veterinary and Animal Sciences	19/08/2017	Coventryvirus SN8	Coventryvirus	Caudoviricetes
AP019560.1	Staphylococcus phage SP120	NA	40530	*Staphylococcus pseudintermedius*	School of Life Science and Technology; Midori-ku 4259, Yokohama, Kanagawa 226-8501	05/04/2019	Coventryvirus SP120	Coventryvirus	Caudoviricetes
AP019561.1	Staphylococcus phage SP197	NA	41149	*Staphylococcus pseudintermedius*	School of Life Science and Technology; Midori-ku 4259, Yokohama, Kanagawa 226-8501	05/04/2019	Coventryvirus SP197	Coventryvirus	Caudoviricetes
AP019562.1	Staphylococcus phage SP276	NA	40147	*Staphylococcus pseudintermedius*	School of Life Science and Technology; Midori-ku 4259, Yokohama, Kanagawa 226-8501	05/04/2019	Coventryvirus SP276	Coventryvirus	Caudoviricetes
MK075003.1	Staphylococcus phage phiSP44-1	NA	42565	*Staphylococcus sp.*	The Rockefeller University, Laboratoy of Bacterial Pathogenesis and Immunology	12/04/2019	Coventryvirus SP441	Coventryvirus	Caudoviricetes
MN028536.1	Staphylococcus phage vB_SpsM_WIS42	Poland	40093	*Staphylococcus pseudintermedius*	Institute of Microbiology, Faculty of Biology, University of Warsaw, Department of Applied Microbiology	14/08/2019	Coventryvirus WIS42	Coventryvirus	Caudoviricetes

Accession	Organism_Name	Country	Length (bp)	Host	Organization	Release_Date	Species	Genus	Family or Class
MT423823.1	Staphylococcus virus pSp_SNUABM-J	South Korea	40224	*Staphylococcus pseudintermedius*	Seoul National University, Laboratory of Aquatic Biomedicine, College of Veterinary Medicine and Research Institute for Veterinary Science	15/06/2020	Staphylococcus virus pSp_SNUABM-J	Coventryvirus	Caudoviricetes
MT423824.1	Staphylococcus virus pSp_SNUABM-S	South Korea	40159	*Staphylococcus pseudintermedius*	Seoul National University, Laboratory of Aquatic Biomedicine, College of Veterinary Medicine and Research Institute for Veterinary Science	15/06/2020	Staphylococcus virus pSp_SNUABM-S	Coventryvirus	Caudoviricetes
OP168678.1	Staphylococcus phage vB_SpsS_VL4	Thailand	39788	*Staphylococcus pseudintermedius*	Mahidol University, Veterinary Diagnostic Center, Faculty of Veterinary science	03/09/2022	Staphylococcus phage vB_SpsS_VL4	Coventryvirus	Caudoviricetes
KX827369.1	Staphylococcus phage SpT152	United Kingdom	41087	*Staphylococcus pseudintermedius*	University of Warwick, School of Life Sciences	23/11/2016	Coventryvirus SpT152	Coventryvirus	Caudoviricetes
KX827370.1	Staphylococcus phage SpT252	United Kingdom	40093	*Staphylococcus pseudintermedius*	University of Warwick, School of Life Sciences	23/11/2016	Coventryvirus WIS42	Coventryvirus	Caudoviricetes
OM373551.1	Staphylococcus phage vB_SpsX-DS3	USA	38198	*Staphylococcus pseudintermedius*	Baylor College of Medicine, Molecular Virology and Microbiology	30/03/2022	Staphylococcus phage vB_SpsX-DS3	Coventryvirus	Caudoviricetes
OM373552.1	Staphylococcus phage vB_SpsX-DS4	USA	38285	*Staphylococcus pseudintermedius*	Baylor College of Medicine, Molecular Virology and Microbiology	30/03/2022	Staphylococcus phage vB_SpsX-DS4	Coventryvirus	Caudoviricetes
OM373553.1	Staphylococcus phage vB_SpsX-DS5	USA	26100	*Staphylococcus pseudintermedius*	Baylor College of Medicine, Molecular Virology and Microbiology	30/03/2022	Staphylococcus phage vB_SpsX-DS5	Coventryvirus	Caudoviricetes
OM439662.1	Staphylococcus phage vB_SauS_277g	Argentina	44016	*Staphylococcus aureus*	Facultad de Ciencias Medicas,	12/02/2022	Staphylococcus phage vB_SauS_277g	Dubowvirus	Caudoviricetes

Accession	Organism_Name	Country	Length (bp)	Host	Organization	Release_Date	Species	Genus	Family or Class
					Universidad Nacional de Rosario, Laboratorio de Microbiologia Molecular				
OM439666.1	Staphylococcus phage vB_SauS_321c	Argentina	42397	*Staphylococcus aureus*	Facultad de Ciencias Medicas, Universidad Nacional de Rosario, Laboratorio de Microbiologia Molecular	12/02/2022	Staphylococcus phage vB_SauS_321c	Dubowvirus	Caudoviricetes
OM439668.1	Staphylococcus phage vB_SauS_713	Argentina	42961	*Staphylococcus aureus*	Facultad de Ciencias Medicas, Universidad Nacional de Rosario, Laboratorio de Microbiologia Molecular	12/02/2022	Staphylococcus phage vB_SauS_713	Dubowvirus	Caudoviricetes
OM439670.1	Staphylococcus phage vB_SauS_775	Argentina	43321	*Staphylococcus aureus*	Facultad de Ciencias Medicas, Universidad Nacional de Rosario, Laboratorio de Microbiologia Molecular	12/02/2022	Staphylococcus phage vB_SauS_775	Dubowvirus	Caudoviricetes
OM439671.1	Staphylococcus phage vB_SauS_832	Argentina	43499	*Staphylococcus aureus*	Facultad de Ciencias Medicas, Universidad Nacional de Rosario, Laboratorio de Microbiologia Molecular	12/02/2022	Staphylococcus phage vB_SauS_832	Dubowvirus	Caudoviricetes
OM439675.1	Staphylococcus phage vB_SauS_Mh4	Argentina	44199	*Staphylococcus aureus*	Facultad de Ciencias Medicas, Universidad Nacional de Rosario, Laboratorio de Microbiologia Molecular	12/02/2022	Staphylococcus phage vB_SauS_Mh4	Dubowvirus	Caudoviricetes
MW554488.1	Staphylococcus phage BUCT556	China	43207	*Staphylococcus cohnii*	Beijing University of Chemical	10/03/2021	Staphylococcus phage BUCT556	Dubowvirus	Caudoviricetes

Accession	Organism_Name	Country	Length (bp)	Host	Organization	Release_Date	Species	Genus	Family or Class
					Technology, College of Life Science and Technology				
NC_054973.1	Staphylococcus phage Baq_Sau1	NA	44384	*Staphylococcus aureus*	National Center for Biotechnology Information, NIH	27/05/2021	Dubowvirus baqsau1	Dubowvirus	Caudoviricetes
CP062454.1	Staphylococcus phage ECel-2020a	NA	42534	*Staphylococcus aureus*	University of central Florida, Burnett School of Biomedical Sciences	07/03/2022	Staphylococcus phage ECel-2020a	Dubowvirus	Caudoviricetes
CP062451.1	Staphylococcus phage ECel-2020b	NA	43194	*Staphylococcus aureus*	University of central Florida, Burnett School of Biomedical Sciences	07/03/2022	Staphylococcus phage ECel-2020b	Dubowvirus	Caudoviricetes
CP062427.1	Staphylococcus phage ECel-2020j	NA	43078	*Staphylococcus aureus*	University of central Florida, Burnett School of Biomedical Sciences	07/03/2022	Staphylococcus phage ECel-2020j	Dubowvirus	Caudoviricetes
MG557619.1	Staphylococcus phage HSA84	NA	42650	*Staphylococcus aureus*	Seoul National University, Department of Food Science and Biotechnology	30/11/2018	Dubowvirus HSA84	Dubowvirus	Caudoviricetes
AF424781.1	Staphylococcus phage phi 11	NA	43604	*Staphylococcus aureus*	Univ. of Oklahoma Health Sciences Center, Microbiology and Immunology	26/02/2002	Dubowvirus dv11	Dubowvirus	Caudoviricetes
AP008953.1	Staphylococcus phage phiETA2	NA	43265	*Staphylococcus aureus*	Graduate School of Biomedical Sciences Dept. Bacteriology; Kasumi 1-2-3, Hiroshima, Hiroshima 734-8553	06/01/2007	Dubowvirus ETA2	Dubowvirus	Caudoviricetes
AB370205.1	Staphylococcus phage phiMR25	NA	44342	*Staphylococcus aureus*	Medical School; Oko-cho, Kohasu, Nankoku, Kochi 783-8505	04/06/2008	Dubowvirus MR25	Dubowvirus	Caudoviricetes
KC677663.1	Staphylococcus phage SA12	NA	42902	*Staphylococcus aureus*	Seoul National University, Department of	30/06/2013	Staphylococcus phage SA12	Dubowvirus	Caudoviricetes

Accession	Organism_Name	Country	Length (bp)	Host	Organization	Release_Date	Species	Genus	Family or Class
					Food and Animal Biotechnology				
KJ716334.1	Staphylococcus phage SA97	NA	40592	*Staphylococcus aureus*	Seoul National University, Food Science & Biotechnology	30/04/2015	Dubowvirus SA97	Dubowvirus	Caudoviricetes
GU477322.1	Staphylococcus phage SAP-26	NA	41207	*Staphylococcus aureus*	Kyungpook National University, Department of Microbiology	18/08/2010	Dubowvirus SAP26	Dubowvirus	Caudoviricetes
MK801682.1	Staphylococcus phage SAP33	NA	42414	*Staphylococcus aureus*	Gachon University, Food Science and Biotechnology	07/07/2019	Dubowvirus SAP33	Dubowvirus	Caudoviricetes
MK801683.1	Staphylococcus phage SAP40	NA	42911	*Staphylococcus aureus*	Gachon University, Food Science and Biotechnology	07/07/2019	Dubowvirus SAP33	Dubowvirus	Caudoviricetes
JX274646.1	Staphylococcus phage SP5	NA	43305	*Staphylococcus aureus*	Seoul National University, Department of Food and Animal Biotechnology	31/07/2013	Dubowvirus SP5	Dubowvirus	Caudoviricetes
JX274647.1	Staphylococcus phage SP6	NA	42902	*Staphylococcus aureus*	Seoul National University, Department of Food and Animal Biotechnology	31/07/2013	Staphylococcus phage SP6	Dubowvirus	Caudoviricetes
EU861004.1	Staphylococcus phage vB_SauS-phiIPLA88	NA	42526	*Staphylococcus aureus*	CSIC, Instituto de Productos Lacteos de Asturias	01/12/2008	Dubowvirus IPLA88	Dubowvirus	Caudoviricetes
JN700519.1	Staphylococcus phage StB27	NA	40071	*Staphylococcus hominis*	ULB, Universite Libre de Bruxelles, Belgium, Genetique et Physiologie Bacterienne, IBMM, DBM	18/02/2012	Staphylococcus phage StB27	Dubowvirus	Caudoviricetes
AY954952.1	Staphylococcus phage 53	NA	43883	*Staphylococcus sp.*	Targanta Therapeutics	05/04/2005	Dubowvirus dv53	Dubowvirus	Caudoviricetes
AY954951.1	Staphylococcus phage 69	NA	42732	*Staphylococcus sp.*	Targanta Therapeutics	05/04/2005	Dubowvirus dv69	Dubowvirus	Caudoviricetes
AY954953.1	Staphylococcus phage 85	NA	44283	*Staphylococcus sp.*	Targanta Therapeutics	05/04/2005	Dubowvirus dv85	Dubowvirus	Caudoviricetes
DQ530359.1	Staphylococcus phage phinm1	NA	43128	*Staphylococcus sp.*	University of Chicago, Microbiology	31/10/2006	Dubowvirus NM1	Dubowvirus	Caudoviricetes

Accession	Organism_Name	Country	Length (bp)	Host	Organization	Release_Date	Species	Genus	Family or Class
DQ530360.1	Staphylococcus phage phinm2	NA	43145	*Staphylococcus sp.*	University of Chicago, Microbiology	31/10/2006	Dubowvirus NM2	Dubowvirus	Caudoviricetes
MT013111.1	Staphylococcus phage SA75	South Korea	43134	*Staphylococcus aureus*	J. Craig Venter Institute, Genomic Medicine	13/02/2020	Dubowvirus SA75	Dubowvirus	Caudoviricetes
JQ779024.1	Staphylococcus phage TEM123	South Korea	43786	*Staphylococcus aureus*	Food Science and Biotechnology, Gachon University	02/05/2012	Staphylococcus phage TEM123	Dubowvirus	Caudoviricetes
MH321492.1	Staphylococcus phage Lorac	USA	43147	*Staphylococcus aureus*	Texas A&M University, Center for Phage Technology, Department of Biochemistry and Biophysics	21/07/2018	Staphylococcus phage Lorac	Dubowvirus	Caudoviricetes
ON911714.1	Staphylococcus phage SAP1_TA-2022	USA	43962	*Staphylococcus aureus*	Rutgers, The State University of New Jersey, Ecology, Evolution and Natural Resources	16/08/2022	Staphylococcus phage SAP1_TA-2022	Dubowvirus	Caudoviricetes
ON911718.1	Staphylococcus phage SAP13_TA-2022	USA	43478	*Staphylococcus aureus*	Rutgers, The State University of New Jersey, Ecology, Evolution and Natural Resources	16/08/2022	Staphylococcus phage SAP13_TA-2022	Dubowvirus	Caudoviricetes
ON911715.1	Staphylococcus phage SAP2_TA-2022	USA	43863	*Staphylococcus aureus*	Rutgers, The State University of New Jersey, Ecology, Evolution and Natural Resources	16/08/2022	Staphylococcus phage SAP2_TA-2022	Dubowvirus	Caudoviricetes
ON911716.1	Staphylococcus phage SAP3_TA-2022	USA	43586	*Staphylococcus aureus*	Rutgers, The State University of New Jersey, Ecology, Evolution and Natural Resources	16/08/2022	Staphylococcus phage SAP3_TA-2022	Dubowvirus	Caudoviricetes
ON911717.1	Staphylococcus phage SAP8_Andrews	USA	42981	*Staphylococcus aureus*	Rutgers, The State University of New Jersey, Ecology, Evolution and Natural Resources	16/08/2022	Staphylococcus phage SAP8_Andrews	Dubowvirus	Caudoviricetes
MK450538.1	Staphylococcus phage vB_SpsS_QT1	Czech Republic	43029	*Staphylococcus pseudintermedius*	Masaryk University, Department of Experimental Biology	29/05/2019	Fibralongavirus QT1	Fibralongavirus	Caudoviricetes

Accession	Organism_Name	Country	Length (bp)	Host	Organization	Release_Date	Species	Genus	Family or Class
MK075004.1	Staphylococcus phage phiSP119-1	NA	49795	*Staphylococcus pseudintermedius*	The Rockefeller University, Laboratoy of Bacterial Pathogenesis and Immunology	12/04/2019	Staphylococcus phage phiSP119-1	Fibralongavirus	Caudoviricetes
MK075001.1	Staphylococcus phage phiSP15-1	NA	45164	*Staphylococcus pseudintermedius*	The Rockefeller University, Laboratoy of Bacterial Pathogenesis and Immunology	12/04/2019	Staphylococcus phage phiSP15-1	Fibralongavirus	Caudoviricetes
AY954954.1	Staphylococcus phage 2638A	NA	41318	*Staphylococcus sp.*	Targanta Therapeutics	05/04/2005	Fibralongavirus fv2638A	Fibralongavirus	Caudoviricetes
OM373550.1	Staphylococcus phage vB_SpsX-DS1	USA	17344	*Staphylococcus pseudintermedius*	Baylor College of Medicine, Molecular Virology and Microbiology	30/03/2022	Staphylococcus phage vB_SpsX-DS1	Fibralongavirus	Caudoviricetes
OM373558.1	Staphylococcus phage vB_SpsX-DS11	USA	38381	*Staphylococcus pseudintermedius*	Baylor College of Medicine, Molecular Virology and Microbiology	30/03/2022	Staphylococcus phage vB_SpsX-DS11	Fibralongavirus	Caudoviricetes
OM373554.1	Staphylococcus phage vB_SpsX-DS6	USA	42714	*Staphylococcus pseudintermedius*	Baylor College of Medicine, Molecular Virology and Microbiology	30/03/2022	Staphylococcus phage vB_SpsX-DS6	Fibralongavirus	Caudoviricetes
OM373555.1	Staphylococcus phage vB_SpsX-DS7	USA	84555	*Staphylococcus pseudintermedius*	Baylor College of Medicine, Molecular Virology and Microbiology	30/03/2022	Staphylococcus phage vB_SpsX-DS7	Fibralongavirus	Caudoviricetes
OM373556.1	Staphylococcus phage vB_SpsX-DS8	USA	35555	*Staphylococcus pseudintermedius*	Baylor College of Medicine, Molecular Virology and Microbiology	30/03/2022	Staphylococcus phage vB_SpsX-DS8	Fibralongavirus	Caudoviricetes
OM373561.1	Staphylococcus phage vB_SpsX-DSP2	USA	41107	*Staphylococcus pseudintermedius*	Baylor College of Medicine, Molecular Virology and Microbiology	30/03/2022	Staphylococcus phage vB_SpsX-DSP2	Fibralongavirus	Caudoviricetes
OL960569.1	Staphylococcus phage APTC_SA_12	Australia	141041	*Staphylococcus aureus*	University of Adelaide, ENT Surgery	19/09/2022	Staphylococcus phage APTC_SA_12	Kayvirus	Herelleviridae
OL960567.1	Staphylococcus phage APTC_SA_2	Australia	137622	*Staphylococcus aureus*	University of Adelaide, ENT Surgery	19/09/2022	Staphylococcus phage APTC_SA_2	Kayvirus	Herelleviridae
OL960568.1	Staphylococcus phage APTC_SA_4	Australia	137551	*Staphylococcus aureus*	University of Adelaide, ENT Surgery	19/09/2022	Staphylococcus phage APTC_SA_4	Kayvirus	Herelleviridae

Accession	Organism_Name	Country	Length (bp)	Host	Organization	Release_Date	Species	Genus	Family or Class
OP263967.1	Staphylococcus phage Biyabeda-mokiny_1	Australia	141091	*Staphylococcus aureus*	Telethon Kids Institute, Epithelial	26/09/2022	Staphylococcus phage Biyabeda-mokiny_1	Kayvirus	Herelleviridae
OP263968.1	Staphylococcus phage Biyabeda-mokiny_2	Australia	140800	*Staphylococcus aureus*	Telethon Kids Institute, Epithelial	26/09/2022	Staphylococcus phage Biyabeda-mokiny_2	Kayvirus	Herelleviridae
MH136584.1	Staphylococcus phage HYZ21	China	139675	*Staphylococcus aureus*	College of Basic Medical Sciences, Jilin University, Department of Pathogen Biology	21/04/2018	Staphylococcus phage HYZ21	Kayvirus	Herelleviridae
KR902361.1	Staphylococcus phage IME-SA118	China	139750	*Staphylococcus aureus*	Beijing Institute of Microbiology and Epidemiology, State Key Laboratory of Pathogen and Biosecurity	15/07/2015	Kayvirus G1	Kayvirus	Herelleviridae
KR908644.1	Staphylococcus phage IME-SA119	China	141028	*Staphylococcus aureus*	Beijing Institute of Microbiology and Epidemiology, State Key Laboratory of Pathogen and Biosecurity	15/07/2015	Kayvirus G1	Kayvirus	Herelleviridae
JX878671.1	Staphylococcus phage JD007	China	141836	*Staphylococcus aureus*	Shanghai Jiao Tong University School of Medicine, Department of Medical Microbiology and Parasitology	21/11/2012	Kayvirus JD7	Kayvirus	Herelleviridae
KY779849.1	Staphylococcus phage qdsa002	China	142499	*Staphylococcus aureus*	Ocean University of China, Food Science and Engineering	20/05/2017	Kayvirus G15	Kayvirus	Herelleviridae
OP235318.1	Staphylococcus phage SYL	China	151066	*Staphylococcus aureus*	College of Basic Medical Sciences, Army Medical University, Department of Microbiology	24/08/2022	Staphylococcus phage SYL	Kayvirus	Herelleviridae
MN304941.1	Staphylococcus phage vB_SauH_IME522	China	140246	*Staphylococcus aureus*	Department of Graduate Hebei North University	25/09/2019	Kayvirus P108	Kayvirus	Herelleviridae
MT786458.1	Staphylococcus phage vB_SauH_SAP1	China	143375	*Staphylococcus aureus*	Yunnan Institute for Endemic Disease	24/08/2020	Kayvirus P108	Kayvirus	Herelleviridae

Accession	Organism_Name	Country	Length (bp)	Host	Organization	Release_Date	Species	Genus	Family or Class
					Prevention and Control, Yunnan Key Laboratory for Zoonosis Control and Prevention				
MH159197.1	Staphylococcus phage VB_SavM_JYL01	China	141384	*Staphylococcus aureus*	Animal Medical College, Jilin University	29/10/2018	Kayvirus P108	Kayvirus	Herelleviridae
MK250904.1	Staphylococcus phage VB-SavM-JYL02	China	141384	*Staphylococcus aureus*	Jinlin University, College of Veterinary Medicine	13/01/2019	Kayvirus P108	Kayvirus	Herelleviridae
MK584893.1	Staphylococcus phage vBSM-A1	China	140654	*Staphylococcus aureus*	Dalian University of Technology, College of Life Sciences	17/04/2019	Kayvirus G1	Kayvirus	Herelleviridae
MK656892.1	Staphylococcus phage vBSP-A2	China	136528	*Staphylococcus aureus*	Dalian University of Technology, College of Life Sciences	10/04/2019	Staphylococcus phage vBSP-A2	Kayvirus	Herelleviridae
MZ427930.2	Staphylococcus phage SAP23	China	151618	*Staphylococcus sp.*	Xinqiao Hospital, Army Medical University, Plastic and Cosmetic Surgery	10/09/2021	Staphylococcus phage SAP23	Kayvirus	Herelleviridae
KJ206563.2	Staphylococcus phage 812	Czech Republic	146666	*Staphylococcus sp.*	Masaryk University, Faculty of Science, Department of Experimental Biology	06/01/2016	Staphylococcus phage 812	Kayvirus	Herelleviridae
MH844529.1	Staphylococcus phage 812h1	Czech Republic	150582	*Staphylococcus sp.*	Masaryk University, Faculty of Science, Department of Experimental Biology	02/12/2018	Staphylococcus phage 812h1	Kayvirus	Herelleviridae
OP323059.1	Staphylococcus phage vB_ScaM-V1SC01	France	139533	*Staphylococcus capitis*	Hospices Civils de Lyon	19/10/2022	Staphylococcus phage vB_ScaM-V1SC01	Kayvirus	Herelleviridae
FR852584.1	Staphylococcus phage ISP	Georgia	138339	*Staphylococcus aureus*	to the INSDC. Vandersteegen K., Biosystems, K.U.Leuven, Kasteelpark Arenberg 21, 3001	31/08/2011	Kayvirus G1	Kayvirus	Herelleviridae
KC012913.1	Staphylococcus phage Team1	Georgia	140903	*Staphylococcus xylosus*	Universite Laval, Biochimie,	29/07/2014	Kayvirus G1	Kayvirus	Herelleviridae

Accession	Organism_Name	Country	Length (bp)	Host	Organization	Release_Date	Species	Genus	Family or Class
					Microbiologie et Bio-Informatique				
MG656408.1	Staphylococcus phage B1	Ireland	148884	*Staphylococcus sp.*	Cork Institute of Technology, Department of Biological Sciences	27/04/2018	Staphylococcus phage B1	Kayvirus	Herelleviridae
MF405094.1	Staphylococcus phage JA1	Ireland	147135	*Staphylococcus sp.*	Cork Institute of Technology, Department of Biological Sciences	27/04/2018	Staphylococcus phage JA1	Kayvirus	Herelleviridae
MW460250.1	Staphylococcus phage SAOMS1	Israel	140135	*Staphylococcus aureus*	Hebrew University, Institute of Dental Sciences and School of Dental Medicine	08/02/2021	Kayvirus G1	Kayvirus	Herelleviridae
LC574321.1	Staphylococcus phage SaGU1	Japan	140909	*Staphylococcus aureus*	Laboratory of Phage Biologics,Graduate School of Medicine; Yanagito, Gifu, Gifu 501-1194	13/05/2021	Kayvirus P108	Kayvirus	Herelleviridae
AB853330.1	Staphylococcus phage S25-3	Japan	139738	*Staphylococcus sp.*	School of Veterinary Medicine, Azabu University; Fuchinobe 1-7-71, Chuo-ku Sagamihara-shi, Kanagawa 252-0206	21/11/2013	Kayvirus S253	Kayvirus	Herelleviridae
AB853331.1	Staphylococcus phage S25-4	Japan	132123	*Staphylococcus sp.*	School of Veterinary Medicine, Azabu University; Fuchinobe 1-7-71, Chuo-ku Sagamihara-shi, Kanagawa 252-0206	21/11/2013	Kayvirus S254	Kayvirus	Herelleviridae
JX080302.2	Staphylococcus phage 676Z	NA	148564	*Staphylococcus aureus*	Institute of Biochemistry and Biophysics, Polish Academy of Sciences, Microbial Biochemistry	14/07/2012	Kayvirus G1	Kayvirus	Herelleviridae

Accession	Organism_Name	Country	Length (bp)	Host	Organization	Release_Date	Species	Genus	Family or Class
JX080301.2	Staphylococcus phage A3R	NA	141018	*Staphylococcus aureus*	Institute of Biochemistry and Biophysics, Polish Academy of Sciences, Microbial Biochemistry	14/07/2012	Kayvirus G1	Kayvirus	Herelleviridae
EU418428.2	Staphylococcus phage A5W	NA	145542	*Staphylococcus aureus*	Institute of Biochemistry and Biophysics, Polish Academy of Sciences., Department of Microbial Biochemistry	28/01/2010	Kayvirus G1	Kayvirus	Herelleviridae
MK331930.1	Staphylococcus phage CH1	NA	138057	*Staphylococcus aureus*	G. N. Gabrichevsky Research Institute for Epidemiology and Microbiology, Laboratory of Clinical Microbiology and Biotechnology of Bacteriophages	06/02/2019	Kayvirus G1	Kayvirus	Herelleviridae
JX080303.2	Staphylococcus phage Fi200W	NA	148481	*Staphylococcus aureus*	Institute of Biochemistry and Biophysics, Polish Academy of Sciences, Microbial Biochemistry	14/07/2012	Kayvirus G1	Kayvirus	Herelleviridae
JQ686190.1	Staphylococcus phage G15	NA	139806	*Staphylococcus aureus*	Jilin University, College of Animal Science and Veterinary Medicine	08/08/2012	Kayvirus G15	Kayvirus	Herelleviridae
MG557618.1	Staphylococcus phage HSA30	NA	140358	*Staphylococcus aureus*	Seoul National University, Department of Food Science and Biotechnology	30/11/2018	Kayvirus P108	Kayvirus	Herelleviridae
KP687431.1	Staphylococcus phage IME-SA1	NA	140218	*Staphylococcus aureus*	Beijing Institute of Microbiology and Epidemiology, State Key Laboratory of Pathogen and Biosecurity	15/04/2015	Kayvirus G1	Kayvirus	Herelleviridae

Accession	Organism_Name	Country	Length (bp)	Host	Organization	Release_Date	Species	Genus	Family or Class
KP687432.1	Staphylococcus phage IME-SA2	NA	140906	*Staphylococcus aureus*	Beijing Institute of Microbiology and Epidemiology, State Key Laboratory of Pathogen and Biosecurity	15/04/2015	Kayvirus G1	Kayvirus	Herelleviridae
MK417516.1	Staphylococcus phage J-Sa36	NA	148312	*Staphylococcus aureus*	Ampliphi Biosciences	30/01/2019	Kayvirus kay	Kayvirus	Herelleviridae
KJ888149.1	Staphylococcus phage MCE-2014	NA	141907	*Staphylococcus aureus*	University of Bath, Dept of Chemistry	02/09/2014	Kayvirus MCE2014	Kayvirus	Herelleviridae
JX080304.2	Staphylococcus phage MSA6	NA	148243	*Staphylococcus aureus*	Institute of Biochemistry and Biophysics, Polish Academy of Sciences, Microbial Biochemistry	14/07/2012	Kayvirus G1	Kayvirus	Herelleviridae
KM216423.1	Staphylococcus phage P108	NA	140807	*Staphylococcus aureus*	Third Military Medical University, Department of Microbiology	26/08/2014	Kayvirus P108	Kayvirus	Herelleviridae
JX080305.2	Staphylococcus phage P4W	NA	147590	*Staphylococcus aureus*	Institute of Biochemistry and Biophysics, Polish Academy of Sciences, Microbial Biochemistry	14/07/2012	Kayvirus G1	Kayvirus	Herelleviridae
AB903967.1	Staphylococcus phage phiSA12	NA	142094	*Staphylococcus aureus*	Department of Bioengineering; 4259 J2-15 Nagatsuta-cho, Yokohama, Kanagawa 226-8501	21/01/2014	Kayvirus SA12	Kayvirus	Herelleviridae
OL362280.1	Staphylococcus phage SA4_Green-2022a	NA	148614	*Staphylococcus aureus*	Baylor College of Medicine, Molecular Virology and Microbiology	27/05/2022	Staphylococcus phage SA4_Green-2022a	Kayvirus	Herelleviridae
JX875065.1	Staphylococcus phage SA5	NA	137031	*Staphylococcus aureus*	Emory University, Biology Department	10/11/2012	Kayvirus G1	Kayvirus	Herelleviridae
MT338525.1	Staphylococcus phage SAM1	NA	147588	*Staphylococcus aureus*	Cork Institute of Technology, Biological Sciences	14/05/2021	Staphylococcus phage SAM1	Kayvirus	Herelleviridae
MT226657.1	Staphylococcus phage SAM2	NA	137617	*Staphylococcus aureus*	Cork Institute of Technology, Biological Sciences	14/05/2021	Kayvirus G1	Kayvirus	Herelleviridae

Accession	Organism_Name	Country	Length (bp)	Host	Organization	Release_Date	Species	Genus	Family or Class
HQ163896.1	Staphylococcus phage Sb1	NA	127188	*Staphylococcus aureus*	G. Eliava Institute of Bacteriophages, Microbiology and Virology	11/07/2011	Kayvirus Sb1	Kayvirus	Herelleviridae
MN336261.1	Staphylococcus phage Sb1_8383	NA	139606	*Staphylococcus aureus*	Walter Reed Army Institute of Research	03/03/2020	Kayvirus G1	Kayvirus	Herelleviridae
MN336262.1	Staphylococcus phage Sb1M_6168	NA	138231	*Staphylococcus aureus*	Walter Reed Army Institute of Research	03/03/2020	Kayvirus G1	Kayvirus	Herelleviridae
MN336263.1	Staphylococcus phage Sb1M_9832	NA	138231	*Staphylococcus aureus*	Walter Reed Army Institute of Research	03/03/2020	Kayvirus G1	Kayvirus	Herelleviridae
JX080300.2	Staphylococcus phage Staph1N	NA	145647	*Staphylococcus aureus*	Institute of Biochemistry and Biophysics, Polish Academy of Sciences, Microbial Biochemistry	14/07/2012	Kayvirus G1	Kayvirus	Herelleviridae
OP651044.1	Staphylococcus phage vb_SaH_Safa	NA	148740	*Staphylococcus aureus*	University of Minho, Centre of Biological Engineering	05/12/2022	Staphylococcus phage vb_SaH_Safa	Kayvirus	Herelleviridae
KY794641.1	Staphylococcus phage vB_Sau_CG	NA	142934	*Staphylococcus aureus*	Facultad de Ciencias Medicas, UNR, Laboratorio de Microbiologia Molecular	03/08/2017	Staphylococcus phage vB_Sau_CG	Kayvirus	Herelleviridae
MW528836.1	Staphylococcus phage vB_SauH_2002	NA	145076	*Staphylococcus aureus*	University of Lausanne, Fundamental Microbiolgy	14/04/2021	Staphylococcus phage vB_SauH_2002	Kayvirus	Herelleviridae
MH107769.1	Staphylococcus phage vB_SauM_0414_108	NA	151627	*Staphylococcus aureus*	Naval Medical Research Center, Biological Defense Research Directorate, Genomics and Bioinformatics	16/04/2018	Staphylococcus phage vB_SauM_0414_108	Kayvirus	Herelleviridae
MF398190.1	Staphylococcus phage vB_SauM-fRuSau02	NA	148464	*Staphylococcus aureus*	University of Helsinki, Research Programs Unit, Immunobiology Research Group	15/08/2017	Staphylococcus phage vB_SauM-fRuSau02	Kayvirus	Herelleviridae
MK417514.1	Staphylococcus virus Sa83	NA	146004	*Staphylococcus aureus*	Ampliphi Biosciences	30/01/2019	Staphylococcus virus Sa83	Kayvirus	Herelleviridae

Accession	Organism_Name	Country	Length (bp)	Host	Organization	Release_Date	Species	Genus	Family or Class
MK417515.1	Staphylococcus virus Sa87	NA	149108	*Staphylococcus aureus*	Ampliphi Biosciences	30/01/2019	Staphylococcus virus Sa87	Kayvirus	Herelleviridae
MW546073.1	Staphylococcus phage BT3	NA	146878	*Staphylococcus sp.*	PhagoMed BioPharma GmbH, Bacteriophage Dept.	16/05/2021	Staphylococcus phage BT3	Kayvirus	Herelleviridae
AY954969.1	Staphylococcus phage G1	NA	138715	*Staphylococcus sp.*	Targanta Therapeutics	05/04/2005	Kayvirus G1	Kayvirus	Herelleviridae
AY176327.1	Staphylococcus phage K	NA	127395	*Staphylococcus sp.*	Dairy Products Research Center	16/04/2004	Kayvirus kay	Kayvirus	Herelleviridae
KP027446.1	Staphylococcus phage phiIPLA-RODI	NA	142348	*Staphylococcus sp.*	IPLA-CSIC, Technology and Biotechnology of Dairy Products	23/03/2015	Kayvirus rodi	Kayvirus	Herelleviridae
AP018375.1	Staphylococcus phage phiSA039	NA	141038	*Staphylococcus sp.*	Department of Bioengineering, School of Life Science and Technology; 4259 J2-15 Nagatsuta-cho, Midori-ku, Yokohama 226-8501	28/02/2018	Staphylococcus phage phiSA039	Kayvirus	Herelleviridae
MW546065.1	Staphylococcus phage PM22	NA	140588	*Staphylococcus sp.*	PhagoMed BioPharma GmbH, Bacteriophage Dept.	16/05/2021	Kayvirus G1	Kayvirus	Herelleviridae
MW546066.1	Staphylococcus phage PM25	NA	142467	*Staphylococcus sp.*	PhagoMed BioPharma GmbH, Bacteriophage Dept.	16/05/2021	Staphylococcus phage PM25	Kayvirus	Herelleviridae
MW546067.1	Staphylococcus phage PM28	NA	142406	*Staphylococcus sp.*	PhagoMed BioPharma GmbH, Bacteriophage Dept.	16/05/2021	Staphylococcus phage PM28	Kayvirus	Herelleviridae
MW546070.1	Staphylococcus phage PM32	NA	148303	*Staphylococcus sp.*	PhagoMed BioPharma GmbH, Bacteriophage Dept.	16/05/2021	Staphylococcus phage PM32	Kayvirus	Herelleviridae
MW546068.1	Staphylococcus phage PM34	NA	143062	*Staphylococcus sp.*	PhagoMed BioPharma GmbH, Bacteriophage Dept.	16/05/2021	Staphylococcus phage PM34	Kayvirus	Herelleviridae
MW546069.1	Staphylococcus phage PM36	NA	143035	*Staphylococcus sp.*	PhagoMed BioPharma GmbH, Bacteriophage Dept.	16/05/2021	Staphylococcus phage PM36	Kayvirus	Herelleviridae

Accession	Organism_Name	Country	Length (bp)	Host	Organization	Release_Date	Species	Genus	Family or Class
MW546074.1	Staphylococcus phage PM4	NA	148627	*Staphylococcus sp.*	PhagoMed BioPharma GmbH, Bacteriophage Dept.	16/05/2021	Staphylococcus phage PM4	Kayvirus	Herelleviridae
MW546064.1	Staphylococcus phage PM9	NA	148495	*Staphylococcus sp.*	PhagoMed BioPharma GmbH, Bacteriophage Dept.	16/05/2021	Staphylococcus phage PM9	Kayvirus	Herelleviridae
MK331931.1	Staphylococcus phage Sa30	NA	146737	*Staphylococcus sp.*	G. N. Gabrichevsky Research Institute for Epidemiology and Microbiology, Laboratory of Clinical Microbiology and Biotechnology of Bacteriophages	06/02/2019	Staphylococcus phage Sa30	Kayvirus	Herelleviridae
OM030343.1	Staphylococcus phage vB_SaS_GE1	NA	138106	*Staphylococcus sp.*	G. Eliava Institute of Bacteriophages, Microbiology and Virology, General Microbiology	23/02/2022	Kayvirus G1	Kayvirus	Herelleviridae
MG721208.1	Staphylococcus phage vB_SauM_LM12	NA	143652	*Staphylococcus sp.*	University of Minho, CEB - Centre of Biological Engineering	03/02/2018	Staphylococcus phage vB_SauM_LM12	Kayvirus	Herelleviridae
MN539736.1	Staphylococcus phage vB_SauM-A	Poland	139088	*Staphylococcus aureus*	Medical University of Gdansk, Department of Medical Microbiology	29/09/2020	Staphylococcus phage vB_SauM-A	Kayvirus	Herelleviridae
MN539737.1	Staphylococcus phage vB_SauM-C	Poland	140086	*Staphylococcus aureus*	Medical University of Gdansk, Department of Medical Microbiology	29/09/2020	Staphylococcus phage vB_SauM-C	Kayvirus	Herelleviridae
MN539738.1	Staphylococcus phage vB_SauM-D	Poland	139031	*Staphylococcus aureus*	Medical University of Gdansk, Department of Medical Microbiology	29/09/2020	Staphylococcus phage vB_SauM-D	Kayvirus	Herelleviridae
KY581279.1	Staphylococcus phage pSa-3	South Korea	137836	*Staphylococcus aureus*	Seoul National University, Laboratory of Aquatic	01/03/2017	Kayvirus G1	Kayvirus	Herelleviridae

Accession	Organism_Name	Country	Length (bp)	Host	Organization	Release_Date	Species	Genus	Family or Class
					Biomedicine, College of Veterinary Medicine and Research Institute for Veterinary Science				
MZ573923.1	Staphylococcus phage vB_ScoM-PSC1	South Korea	140342	*Staphylococcus ureilyticus*	Korea Research Institute of Bioscience, Institution Disease Research Center	06/09/2021	Kayvirus P108	Kayvirus	Herelleviridae
MT080585.1	Staphylococcus phage vB_SauM_EW18	United Kingdom	143247	*Staphylococcus carnosus*	Manchester Metropolitan University, Department of Life Sciences	07/03/2020	Staphylococcus phage vB_SauM_EW18	Kayvirus	Herelleviridae
MT080589.1	Staphylococcus phage vB_SauM_EW26	United Kingdom	143240	*Staphylococcus carnosus*	Manchester Metropolitan University, Department of Life Sciences	07/03/2020	Staphylococcus phage vB_SauM_EW26	Kayvirus	Herelleviridae
MT080591.1	Staphylococcus phage vB_SauM_EW29	United Kingdom	143240	*Staphylococcus carnosus*	Manchester Metropolitan University, Department of Life Sciences	07/03/2020	Staphylococcus phage vB_SauM_EW29	Kayvirus	Herelleviridae
MT080600.1	Staphylococcus phage vB_SauM_EW71	United Kingdom	139939	*Staphylococcus carnosus*	Manchester Metropolitan University, Department of Life Sciences	07/03/2020	Staphylococcus phage vB_SauM_EW71	Kayvirus	Herelleviridae
MT080601.1	Staphylococcus phage vB_SauM_EW72	United Kingdom	143240	*Staphylococcus carnosus*	Manchester Metropolitan University, Department of Life Sciences	07/03/2020	Staphylococcus phage vB_SauM_EW72	Kayvirus	Herelleviridae
MT080602.1	Staphylococcus phage vB_SauM_EW74	United Kingdom	139896	*Staphylococcus carnosus*	Manchester Metropolitan University, Department of Life Sciences	07/03/2020	Staphylococcus phage vB_SauM_EW74	Kayvirus	Herelleviridae
MT554104.1	Staphylococcus phage ESa1	USA	153106	*Staphylococcus aureus*	J. Craig Venter Institute, Genomic Medicine	18/06/2020	Staphylococcus phage ESa1	Kayvirus	Herelleviridae
MN045228.1	Staphylococcus phage Maine	USA	141712	*Staphylococcus aureus*	Texas A&M University, Center	11/09/2019	Staphylococcus phage Maine	Kayvirus	Herelleviridae

Accession	Organism_Name	Country	Length (bp)	Host	Organization	Release_Date	Species	Genus	Family or Class
					for Phage Technology				
OM373557.1	Staphylococcus phage vB_SpsM-DS10	USA	148313	*Staphylococcus pseudintermedius*	Baylor College of Medicine, Molecular Virology and Microbiology	30/03/2022	Staphylococcus phage vB_SpsM-DS10	Kayvirus	Herelleviridae
MT411892.1	Staphylococcus phage Metroid	USA	150935	*Staphylococcus sp.*	University of Pittsburgh, Department of Biological Sciences	03/06/2020	Staphylococcus phage Metroid	Kayvirus	Herelleviridae
NC_008689.1	Staphylococcus phage phiPVL108	NA	44857	*Staphylococcus aureus*	not available	not available	not available	not defined	not defined
NC_004740.1	Staphylococcus prophage phiN315	NA	44082	*Staphylococcus aureus*	not available	not available	not available	not defined	not defined
NC_004616.1	Staphylococcus prophage phi 12	NA	44970	*Staphylococcus aureus*	not available	not available	not available	not defined	not defined
NC_002486.1	Staphylococcus prophage phiPV83	NA	45636	*Staphylococcus aureus*	not available	not available	not available	not defined	not defined
NC_002321.1	Staphylococcus phage PVL	NA	41401	*Staphylococcus aureus*	not available	not available	not available	not defined	not defined
PQ281812.1	Staphylococcus phage phiSabovST1	NA	50001	*Staphylococcus aureus*	not available	not available	not available	not defined	not defined
PP554657.1	Staphylococcus phage mosaic phi11	NA	42020	*Staphylococcus aureus*	not available	not available	not available	not defined	not defined
PP544178.1	Staphylococcus phage vB_SauS_26	NA	42061	*Staphylococcus aureus*	not available	not available	not available	not defined	not defined
PP197657.1	Staphylococcus phage Rosita	NA	44221	*Staphylococcus aureus*	not available	not available	not available	not defined	not defined
PP098621.1	Staphylococcus phage 2339	NA	43150	*Staphylococcus aureus*	not available	not available	not available	not defined	not defined
PP098622.1	Staphylococcus phage 6199	NA	43070	*Staphylococcus aureus*	not available	not available	not available	not defined	not defined
PP098624.1	Staphylococcus phage 7206	NA	44049	*Staphylococcus aureus*	not available	not available	not available	not defined	not defined
OQ851348.1	Staphylococcus phage MSP1	NA	43615	*Staphylococcus aureus*	not available	not available	not available	not defined	not defined
OQ578993.1	Staphylococcus phage phiST9-A	NA	48241	*Staphylococcus aureus*	not available	not available	not available	not defined	not defined
OQ578994.1	Staphylococcus phage phiST9-B	NA	46871	*Staphylococcus aureus*	not available	not available	not available	not defined	not defined
OQ578995.1	Staphylococcus phage phiST9-C	NA	48173	*Staphylococcus aureus*	not available	not available	not available	not defined	not defined
MZ041098.1	Staphylococcus phage DSP07	NA	42483	*Staphylococcus aureus*	not available	not available	not available	not defined	not defined
OR453397.1	Staphylococcus phage J1	NA	43965	*Staphylococcus sp.*	not available	not available	not available	not defined	not defined

Accession	Organism_Name	Country	Length (bp)	Host	Organization	Release_Date	Species	Genus	Family or Class
OR453398.1	Staphylococcus phage J2	NA	43965	*Staphylococcus sp.*	not available	not available	not available	not defined	not defined
OR453399.1	Staphylococcus phage J4	NA	42921	*Staphylococcus sp.*	not available	not available	not available	not defined	not defined
ON513432.1	Staphylococcus phage ASZ22RN	Poland	43579	*Staphylococcus aureus*	not available	not available	not available	not defined	not defined
MW846279.1	Staphylococcus phage SA3821	South Korea	43417	*Staphylococcus aureus*	not available	not available	not available	not defined	not defined
OP661216.1	Staphylococcus phage phiSa2wa-st80.2	Australia	45164	*Staphylococcus aureus*	not available	not available	not defined	not defined	not defined
OP661217.1	Staphylococcus phage phiSa2wa-st80.4	Australia	45164	*Staphylococcus aureus*	not available	not available	not defined	not defined	not defined
OP594307.1	Staphylococcus phage Henu2 isolate PhiV_005	Brazil	43513	*Staphylococcus aureus*	not available		not defined	not defined	not defined
OQ828659.1	Staphylococcus phage SapYZUs7	China	43682	*Staphylococcus aureus*	not available	not available	not defined	not defined	not defined
PQ826494.1	Staphylococcus phage vB_SauS_Q5	China	46027	*Staphylococcus aureus*	not available	not available	not defined	not defined	not defined
OR479082.1	Staphylococcus phage FJ04	China	38529	*Staphylococcus aureus*	not available	not available	not defined	not defined	not defined
MZ570582.1	Staphylococcus phage SAP_1432	China	40357	*Staphylococcus aureus*	not available	not available	not defined	not defined	not defined
OQ687052.1	Staphylococcus phage L-m44	China	42496	*Staphylococcus aureus*	not available	not available	not defined	not defined	not defined
OM687507.1	Staphylococcus phage PHB22a	China	44558	*Staphylococcus aureus*	not available	not available	not defined	not defined	not defined
OM687508.1	Staphylococcus phage PHB25a	China	33683	*Staphylococcus aureus*	not available	not available	not defined	not defined	not defined
OM687509.1	Staphylococcus phage PHB38a	China	46448	*Staphylococcus aureus*	not available	not available	not defined	not defined	not defined
OM687510.1	Staphylococcus phage PHB40a	China	47749	*Staphylococcus aureus*	not available	not available	not defined	not defined	not defined
PV036961.1	Staphylococcus phage vB_SepS_E72m5	Czech Republic	44155	*Staphylococcus epidermidis*	not available	not available	not defined	not defined	not defined
PV069679.1	Staphylococcus phage vB_Sau_FK1	Germany	44955	*Staphylococcus aureus*	not available	not available	not defined	not defined	not defined
PV069681.1	Staphylococcus phage vB_Sau_IS1	Germany	42537	*Staphylococcus aureus*	not available	not available	not defined	not defined	not defined
LC680885.1	Staphylococcus phage S6	Japan	267055	*Staphylococcus aureus*	Department of Bacteriology, Graduate School of Medicine Dentistry and Pharmaceutical Sciences; Shikata-cho 2-5-1, Kita-ku, Okayama City,	01/02/2022	Staphylococcus phage S6	not defined	Caudoviricetes

Accession	Organism_Name	Country	Length (bp)	Host	Organization	Release_Date	Species	Genus	Family or Class
					Okayama 700-8558				
MN091625.1	Staphylococcus phage PALS_1	NA	138776	*Staphylococcus aureus*	Seoul National University, Food Biotechnology, College of Agriculture and Life Sciences	30/06/2021	Staphylococcus phage PALS_1	not defined	Herelleviridae
MN091626.1	Staphylococcus phage PALS_2	NA	268748	*Staphylococcus aureus*	Seoul National University, Food Biotechnology, College of Agriculture and Life Sciences	11/03/2021	Staphylococcus phage PALS_2	not defined	Caudoviricetes
AY954948.1	Staphylococcus phage PT1028	NA	15603	*Staphylococcus aureus*	Targanta Therapeutics	05/04/2005	Staphylococcus phage PT1028	not defined	Caudoviricetes
KY794642.1	Staphylococcus phage vB_Sau_Clo6	NA	143734	*Staphylococcus aureus*	Facultad de Ciencias Medicas, UNR, Laboratorio de Microbiologia Molecular	03/08/2017	Staphylococcus phage vB_Sau_Clo6	not defined	Herelleviridae
KY794643.1	Staphylococcus phage vB_Sau_S24	NA	139997	*Staphylococcus aureus*	Facultad de Ciencias Medicas, UNR, Laboratorio de Microbiologia Molecular	03/08/2017	Staphylococcus phage vB_Sau_S24	not defined	Herelleviridae
MT787017.1	Staphylococcus phage WV	NA	141342	*Staphylococcus aureus*	Ningbo University, School of Marine Sciences	29/09/2020	Staphylococcus phage WV	not defined	Herelleviridae
OP328423.1	Staphylococcus phage phiSa2wa-st1930	Australia	44629	*Staphylococcus aureus*	Curtin University, School of Biomedical Sciences	08/10/2022	Staphylococcus phage phiSa2wa-st1930	not defined	Caudoviricetes
MZ779063.1	Staphylococcus phage vB_SauM-UFV_DC4	Brazil	263185	*Staphylococcus aureus*	Universita Degli Studi di Padova, DAFNAE	14/09/2021	Staphylococcus phage vB_SauM-UFV_DC4	not defined	Caudoviricetes
MZ488273.1	Staphylococcus phage vB_SarS_BM31	China	42271	*Staphylococcus arlettae*	Sichuan Agricultural University, College of Veterinary Medicine	28/07/2021	Staphylococcus phage vB_SarS_BM31	not defined	Caudoviricetes
OL741057.1	Staphylococcus phage vB_Sau_P68	China	139409	*Staphylococcus aureus*	Academy of Military Medical Sciences, Institute of Microbiology and Epidemiology	31/01/2022	Staphylococcus phage vB_Sau_P68	not defined	Herelleviridae

Accession	Organism_Name	Country	Length (bp)	Host	Organization	Release_Date	Species	Genus	Family or Class
KX156762.1	Staphylococcus phage vB_SauS_IMEP5	China	44677	*Staphylococcus aureus*	Beijing Institute of Microbiology and Epidemiology, State Key Laboratory of Pathogen and Biosecurity	30/10/2016	Staphylococcus phage vB_SauS_IMEP5	not defined	Caudoviricetes
KP735928.1	Staphylococcus phage IME-SA4	China	41843	*Staphylococcus haemolyticus*	Beijing Institute of Microbiology and Epidemiology, State Key Laboratory of Pathogen and Biosecurity	17/03/2015	Staphylococcus phage IME-SA4	not defined	Caudoviricetes
CP018841.1	Staphylococcus phage HOB 14.1.R1	Denmark	18659	*Staphylococcus epidermidis*	Aarhus University, Biomedicine	05/01/2017	Staphylococcus phage HOB 14.1.R1	not defined	Caudoviricetes
OP297178.1	Staphylococcus phage vB_ScaM-V1SC04	France	138478	*Staphylococcus capitis*	Hospices Civils de Lyon	08/10/2022	Staphylococcus phage vB_ScaM-V1SC04	not defined	Herelleviridae
ON325435.2	Staphylococcus phage CUB-EPI_14	Germany	46098	*Staphylococcus epidermidis*	KU Leuven, Biosystems	31/05/2022	Staphylococcus phage CUB-EPI_14	not defined	Caudoviricetes
MT880870.1	Staphylococcus phage PhiSepi-HH1	Germany	34053	*Staphylococcus epidermidis*	Institute of Medical Microbiology, Virology and Hygiene, Center for Diagnostics	09/02/2021	Staphylococcus phage PhiSepi-HH1	not defined	Caudoviricetes
MT880872.1	Staphylococcus phage PhiSepi-HH3	Germany	147057	*Staphylococcus epidermidis*	Institute of Medical Microbiology, Virology and Hygiene, Center for Diagnostics	09/02/2021	Staphylococcus phage PhiSepi-HH3	not defined	Caudoviricetes
MT880871.1	Staphylococcus phage PI-Sepi-HH2	Germany	36164	*Staphylococcus epidermidis*	Institute of Medical Microbiology, Virology and Hygiene, Center for Diagnostics	09/02/2021	Staphylococcus phage PI-Sepi-HH2	not defined	Caudoviricetes
KF589919.1	Staphylococcus phage phiRS7	Israel	43394	*Staphylococcus saprophyticus*	San Diego State University, Department of Biology and Center for Microbial Sciences	29/09/2013	Staphylococcus phage phiRS7	not defined	Caudoviricetes
CP062450.1	Staphylococcus phage ECel-2020c	NA	32456	*Staphylococcus aureus*	University of central Florida, Burnett School of Biomedical Sciences	07/03/2022	Staphylococcus phage ECel-2020c	not defined	Caudoviricetes

Accession	Organism_Name	Country	Length (bp)	Host	Organization	Release_Date	Species	Genus	Family or Class
JN700521.1	Staphylococcus phage StB20	NA	40917	*Staphylococcus capitis*	ULB, Universite Libre de Bruxelles, Belgium, Genetique et Physiologie Bacterienne, IBMM, DBM	18/02/2012	Staphylococcus phage StB20	not defined	Caudoviricetes
KY653117.1	Staphylococcus phage IME1323_01	NA	44282	*Staphylococcus caprae*	Beijing Institute of Microbiology and Epidemiology, State Key Laboratory of Pathogen	06/05/2017	Staphylococcus phage IME1323_01	not defined	Caudoviricetes
KT429160.1	Staphylococcus phage SPbeta-like	NA	127726	*Staphylococcus epidermidis*	RIPCM, Bioinformatics	11/10/2015	Staphylococcus phage SPbeta-like	not defined	Caudoviricetes
KT429161.1	Staphylococcus phage StB20-like	NA	40670	*Staphylococcus epidermidis*	RIPCM, Bioinformatics	11/10/2015	Staphylococcus phage StB20-like	not defined	Caudoviricetes
KY653116.1	Staphylococcus phage IME1318_01	NA	45236	*Staphylococcus haemolyticus*	Beijing Institute of Microbiology and Epidemiology, State Key Laboratory of Pathogen	06/05/2017	Staphylococcus phage IME1318_01	not defined	Caudoviricetes
JN700520.2	Staphylococcus phage StB12	NA	44714	*Staphylococcus hominis*	ULB, Universite Libre de Bruxelles, Belgium, Genetique et Physiologie Bacterienne, IBMM, DBM	18/02/2012	Staphylococcus phage StB12	not defined	Caudoviricetes
LC647030.1	Staphylococcus phage vB_SsapH-Golestan-100	NA	136433	*Staphylococcus saprophyticus*	Laboratory Sciences Research Center; Shast Kola, Gorgan, Golestan 4934174515	25/08/2021	Staphylococcus phage vB_SsapH-Golestan-100	not defined	Herelleviridae
LC648442.1	Staphylococcus phage vB_SsapH-Golestan-105-M	NA	142192	*Staphylococcus saprophyticus*	Laboratory Sciences Research Center; Shast Kola, Gorgan, Golestan 4934174515	26/08/2021	Staphylococcus phage vB_SsapH-Golestan-105-M	not defined	Herelleviridae
KY653124.1	Staphylococcus phage IME1367_01	NA	43135	*Staphylococcus warneri*	Beijing Institute of Microbiology and Epidemiology, State Key	06/05/2017	Staphylococcus phage IME1367_01	not defined	Caudoviricetes

Accession	Organism_Name	Country	Length (bp)	Host	Organization	Release_Date	Species	Genus	Family or Class
					Laboratory of Pathogen				
GU169904.1	Staphylococcus phage SA1	South Korea	147303	*Staphylococcus aureus*	Korea University, College of Life Science and Biotechnology	05/12/2009	Staphylococcus phage SA1	not defined	Caudoviricetes
KX011028.1	Staphylococcus phage pSco-10	South Korea	101986	*Staphylococcus cohnii*	Seoul National University, College of Veterinary Medicine	02/06/2016	Staphylococcus phage pSco-10	not defined	Herelleviridae
MZ417326.1	Staphylococcus phage PG-2021_27	Switzerland	128279	*Staphylococcus aureus*	Institue of Marine Sciences (ICM) CSIC, Department of Marine Biology and Oceanography	24/09/2021	Staphylococcus phage PG-2021_27	not defined	Herelleviridae
MZ417327.1	Staphylococcus phage PG-2021_29	Switzerland	131570	*Staphylococcus epidermidis*	Institue of Marine Sciences (ICM) CSIC, Department of Marine Biology and Oceanography	24/09/2021	Staphylococcus phage PG-2021_29	not defined	Herelleviridae
MZ417332.1	Staphylococcus phage PG-2021_38	Switzerland	140647	*Staphylococcus epidermidis*	Institue of Marine Sciences (ICM) CSIC, Department of Marine Biology and Oceanography	24/09/2021	Staphylococcus phage PG-2021_38	not defined	Herelleviridae
MZ417350.1	Staphylococcus phage PG-2021_8	Switzerland	139709	*Staphylococcus equorum*	Institue of Marine Sciences (ICM) CSIC, Department of Marine Biology and Oceanography	24/09/2021	Staphylococcus phage PG-2021_8	not defined	Herelleviridae
MZ417340.1	Staphylococcus phage PG-2021_64	Switzerland	142287	*Staphylococcus succinus*	Institue of Marine Sciences (ICM) CSIC, Department of Marine Biology and Oceanography	24/09/2021	Staphylococcus phage PG-2021_64	not defined	Herelleviridae
MZ417315.1	Staphylococcus phage PG-2021_10	Switzerland	141528	*Staphylococcus xylosus*	Institue of Marine Sciences (ICM) CSIC, Department of Marine Biology and Oceanography	24/09/2021	Staphylococcus phage PG-2021_10	not defined	Herelleviridae
MZ417318.1	Staphylococcus phage PG-2021_15	Switzerland	145090	*Staphylococcus xylosus*	Institue of Marine Sciences (ICM) CSIC, Department of Marine Biology and Oceanography	24/09/2021	Staphylococcus phage PG-2021_15	not defined	Herelleviridae
MZ417319.1	Staphylococcus phage PG-2021_16	Switzerland	141321	*Staphylococcus xylosus*	Institue of Marine Sciences (ICM)	24/09/2021	Staphylococcus phage PG-2021_16	not defined	Herelleviridae

Accession	Organism_Name	Country	Length (bp)	Host	Organization	Release_Date	Species	Genus	Family or Class
					CSIC, Department of Marine Biology and Oceanography				
MZ417320.1	Staphylococcus phage PG-2021_17	Switzerland	144971	*Staphylococcus xylosus*	Institue of Marine Sciences (ICM) CSIC, Department of Marine Biology and Oceanography	24/09/2021	Staphylococcus phage PG-2021_17	not defined	Herelleviridae
MZ417321.1	Staphylococcus phage PG-2021_18	Switzerland	138844	*Staphylococcus xylosus*	Institue of Marine Sciences (ICM) CSIC, Department of Marine Biology and Oceanography	24/09/2021	Staphylococcus phage PG-2021_18	not defined	Herelleviridae
MZ417331.1	Staphylococcus phage PG-2021_35	Switzerland	138653	*Staphylococcus xylosus*	Institue of Marine Sciences (ICM) CSIC, Department of Marine Biology and Oceanography	24/09/2021	Staphylococcus phage PG-2021_35	not defined	Herelleviridae
MZ417337.1	Staphylococcus phage PG-2021_47	Switzerland	142885	*Staphylococcus xylosus*	Institue of Marine Sciences (ICM) CSIC, Department of Marine Biology and Oceanography	24/09/2021	Staphylococcus phage PG-2021_47	not defined	Herelleviridae
MZ417345.1	Staphylococcus phage PG-2021_84	Switzerland	141291	*Staphylococcus xylosus*	Institue of Marine Sciences (ICM) CSIC, Department of Marine Biology and Oceanography	24/09/2021	Staphylococcus phage PG-2021_84	not defined	Herelleviridae
MZ417346.1	Staphylococcus phage PG-2021_86	Switzerland	141965	*Staphylococcus xylosus*	Institue of Marine Sciences (ICM) CSIC, Department of Marine Biology and Oceanography	24/09/2021	Staphylococcus phage PG-2021_86	not defined	Herelleviridae
MZ417347.1	Staphylococcus phage PG-2021_87	Switzerland	141212	*Staphylococcus xylosus*	Institue of Marine Sciences (ICM) CSIC, Department of Marine Biology and Oceanography	24/09/2021	Staphylococcus phage PG-2021_87	not defined	Herelleviridae
MZ417354.1	Staphylococcus phage PG-2021_9	Switzerland	141528	*Staphylococcus xylosus*	Institue of Marine Sciences (ICM) CSIC, Department of Marine Biology and Oceanography	24/09/2021	Staphylococcus phage PG-2021_9	not defined	Herelleviridae
OM850392.1	Staphylococcus phage B2-102	Turkey	142419	*Staphylococcus aureus*	Institute of Health Science, Food Hygine and Technology	13/04/2022	Staphylococcus phage B2-102	not defined	Herelleviridae

Accession	Organism_Name	Country	Length (bp)	Host	Organization	Release_Date	Species	Genus	Family or Class
OM850391.1	Staphylococcus phage O1-102	Turkey	142419	*Staphylococcus aureus*	Institute of Health Science, Food Hygine and Technology	13/04/2022	Staphylococcus phage O1-102	not defined	Herelleviridae
ON113345.1	Staphylococcus phage O1-69	Turkey	41622	*Staphylococcus aureus*	Institute of Health Science, Veterinary Public Health	22/05/2022	Staphylococcus phage O1-69	not defined	Caudoviricetes
ON113344.1	Staphylococcus phage T2-102	Turkey	142419	*Staphylococcus aureus*	Institute of Health Science, Veterinary Public Health	22/05/2022	Staphylococcus phage T2-102	not defined	Herelleviridae
MT080582.1	Staphylococcus phage vB_SauM_EW1	United Kingdom	140906	*Staphylococcus carnosus*	Manchester Metropolitan University, Department of Life Sciences	07/03/2020	Staphylococcus phage vB_SauM_EW1	not defined	Herelleviridae
MT080583.1	Staphylococcus phage vB_SauM_EW13	United Kingdom	145736	*Staphylococcus carnosus*	Manchester Metropolitan University, Department of Life Sciences	07/03/2020	Staphylococcus phage vB_SauM_EW13	not defined	Herelleviridae
MT080586.1	Staphylococcus phage vB_SauM_EW2	United Kingdom	140906	*Staphylococcus carnosus*	Manchester Metropolitan University, Department of Life Sciences	07/03/2020	Staphylococcus phage vB_SauM_EW2	not defined	Herelleviridae
MT080592.1	Staphylococcus phage vB_SauM_EW3	United Kingdom	143283	*Staphylococcus carnosus*	Manchester Metropolitan University, Department of Life Sciences	07/03/2020	Staphylococcus phage vB_SauM_EW3	not defined	Herelleviridae
MT080593.1	Staphylococcus phage vB_SauM_EW36	United Kingdom	139881	*Staphylococcus carnosus*	Manchester Metropolitan University, Department of Life Sciences	07/03/2020	Staphylococcus phage vB_SauM_EW36	not defined	Herelleviridae
MT080594.1	Staphylococcus phage vB_SauM_EW4	United Kingdom	140906	*Staphylococcus carnosus*	Manchester Metropolitan University, Department of Life Sciences	07/03/2020	Staphylococcus phage vB_SauM_EW4	not defined	Herelleviridae
MT080596.1	Staphylococcus phage vB_SauM_EW42	United Kingdom	139874	*Staphylococcus carnosus*	Manchester Metropolitan University, Department of Life Sciences	07/03/2020	Staphylococcus phage vB_SauM_EW42	not defined	Herelleviridae
MT080597.1	Staphylococcus phage vB_SauM_EW5	United Kingdom	143283	*Staphylococcus carnosus*	Manchester Metropolitan	07/03/2020	Staphylococcus phage vB_SauM_EW5	not defined	Herelleviridae

Accession	Organism_Name	Country	Length (bp)	Host	Organization	Release_Date	Species	Genus	Family or Class
					University, Department of Life Sciences				
MT080598.1	Staphylococcus phage vB_SauM_EW6	United Kingdom	143283	*Staphylococcus carnosus*	Manchester Metropolitan University, Department of Life Sciences	07/03/2020	Staphylococcus phage vB_SauM_EW6	not defined	Herelleviridae
MT080599.1	Staphylococcus phage vB_SauM_EW7	United Kingdom	143288	*Staphylococcus carnosus*	Manchester Metropolitan University, Department of Life Sciences	07/03/2020	Staphylococcus phage vB_SauM_EW7	not defined	Herelleviridae
MT080603.1	Staphylococcus phage vB_SauM_EW9	United Kingdom	141953	*Staphylococcus carnosus*	Manchester Metropolitan University, Department of Life Sciences	07/03/2020	Staphylococcus phage vB_SauM_EW9	not defined	Herelleviridae
MW349128.1	Staphylococcus phage Machias	USA	274478	*Staphylococcus aureus*	TAMU, Department of Biochemistry and Biophysics, Center for Phage Technology	18/01/2021	Staphylococcus phage Machias	not defined	Caudoviricetes
MW349129.1	Staphylococcus phage Madawaska	USA	265446	*Staphylococcus aureus*	TAMU, Department of Biochemistry and Biophysics, Center for Phage Technology	18/01/2021	Staphylococcus phage Madawaska	not defined	Caudoviricetes
MW248466.1	Staphylococcus phage MarsHill	USA	266637	*Staphylococcus aureus*	TAMU, Department of Biochemistry and Biophysics, Center for Phage Technology	13/01/2021	Staphylococcus phage MarsHill	not defined	Caudoviricetes
OP142323.1	Staphylococcus phage Lacachita	USA	46473	*Staphylococcus epidermidis*	Rutgers, The State University of New Jersey, Ecology, Evolution and Natural Resources	24/08/2022	Staphylococcus phage Lacachita	not defined	Caudoviricetes
ON550478.1	Staphylococcus phage Sazerac	USA	46428	*Staphylococcus epidermidis*	University of Illinois at Urbana-Champaign, Microbiology	27/06/2022	Staphylococcus phage Sazerac	not defined	Caudoviricetes
OM373548.1	Staphylococcus phage vB_SpsM-DH2	USA	153381	*Staphylococcus pseudintermedius*	Baylor College of Medicine, Molecular Virology and Microbiology	30/03/2022	Staphylococcus phage vB_SpsM-DH2	not defined	Herelleviridae

Accession	Organism_Name	Country	Length (bp)	Host	Organization	Release_Date	Species	Genus	Family or Class
OM373560.1	Staphylococcus phage vB_SpsX-DSP1	USA	16895	*Staphylococcus pseudintermedius*	Baylor College of Medicine, Molecular Virology and Microbiology	30/03/2022	Staphylococcus phage vB_SpsX-DSP1	not defined	Rountreeviridae
OM439664.1	Staphylococcus phage vB_SauS_308	Argentina	39968	*Staphylococcus aureus*	Facultad de Ciencias Medicas, Universidad Nacional de Rosario, Laboratorio de Microbiologia Molecular	12/02/2022	Staphylococcus phage vB_SauS_308	Peeveelvirus	Caudoviricetes
OP136133.1	Staphylococcus phage phiSa2wa-st30.2	Australia	41346	*Staphylococcus aureus*	Curtin University, School of Biomedical Sciences	26/10/2022	Staphylococcus phage phiSa2wa-st30.2	Peeveelvirus	Caudoviricetes
MW650841.1	Staphylococcus phage B_UFSM1	Brazil	41971	*Staphylococcus aureus*	Universidade Federal de Santa Maria, Medicina Veterinaria Preventiva	24/09/2021	Staphylococcus phage B_UFSM1	Peeveelvirus	Caudoviricetes
MW627293.1	Staphylococcus phage B_UFSM3	Brazil	42835	*Staphylococcus aureus*	Universidade Federal de Santa Maria, Medicina Veterinaria Preventiva	24/09/2021	Staphylococcus phage B_UFSM3	Peeveelvirus	Caudoviricetes
MW147366.1	Staphylococcus phage B_UFSM4	Brazil	41290	*Staphylococcus aureus*	Universidade Federal de Santa Maria, Medicina Veterinaria Preventiva	21/06/2021	Staphylococcus phage B_UFSM4	Peeveelvirus	Caudoviricetes
MW192778.1	Staphylococcus phage B_UFSM5	Brazil	41732	*Staphylococcus aureus*	Universidade Federal de Santa Maria, Medicina Veterinaria Preventiva	21/06/2021	Staphylococcus phage B_UFSM5	Peeveelvirus	Caudoviricetes
KX232515.1	Staphylococcus phage 3 AJ-2017	Colombia	43922	*Staphylococcus aureus*	Universidad el Bosque, Bacterial Molecular Genetics Laboratory	24/01/2017	Peeveelvirus pv3AJ2017	Peeveelvirus	Caudoviricetes
CP062426.1	Staphylococcus phage ECel-2020k	NA	41529	*Staphylococcus aureus*	University of central Florida, Burnett School of Biomedical Sciences	07/03/2022	Staphylococcus phage ECel-2020k	Peeveelvirus	Caudoviricetes
CP062457.1	Staphylococcus phage ECel-2020n	NA	40081	*Staphylococcus aureus*	University of central Florida,	07/03/2022	Staphylococcus phage ECel-2020n	Peeveelvirus	Caudoviricetes

Accession	Organism_Name	Country	Length (bp)	Host	Organization	Release_Date	Species	Genus	Family or Class
					Burnett School of Biomedical Sciences				
CP062445.1	Staphylococcus phage ECel-2020o	NA	39181	*Staphylococcus aureus*	University of central Florida, Burnett School of Biomedical Sciences	07/03/2022	Staphylococcus phage ECel-2020o	Peeveelvirus	Caudoviricetes
CP062435.1	Staphylococcus phage ECel-2020p	NA	39181	*Staphylococcus aureus*	University of central Florida, Burnett School of Biomedical Sciences	07/03/2022	Staphylococcus phage ECel-2020p	Peeveelvirus	Caudoviricetes
KY653125.1	Staphylococcus phage IME1346_01	NA	42689	*Staphylococcus aureus*	Beijing Institute of Microbiology and Epidemiology, State Key Laboratory of Pathogen	06/05/2017	Peeveelvirus pv13	Peeveelvirus	Caudoviricetes
KC342645.2	Staphylococcus phage JS01	NA	43458	*Staphylococcus aureus*	Nanjing Agricultural University, College of Veterinary Medicine	24/06/2013	Peeveelvirus JS01	Peeveelvirus	Caudoviricetes
AF424783.1	Staphylococcus phage phi 13	NA	42722	*Staphylococcus aureus*	Univ. of Oklahoma Health Sciences Center, Microbiology and Immunology	26/02/2002	Peeveelvirus pv13	Peeveelvirus	Caudoviricetes
FJ713816.1	Staphylococcus phage phiPVL-CN125	NA	44492	*Staphylococcus aureus*	China Medical School, Medical Microbiology and Parasitology	06/06/2009	Peeveelvirus CN125	Peeveelvirus	Caudoviricetes
OP493557.1	Staphylococcus phage phiSa2-NARSA483	NA	42927	*Staphylococcus aureus*	Curtin University, School of Biomedical Sciences	19/10/2022	Staphylococcus phage phiSa2-NARSA483	Peeveelvirus	Caudoviricetes
KY695153.1	Staphylococcus phage SA7	NA	34730	*Staphylococcus aureus*	Kyung Hee University, Department of Genetic Engineering	29/03/2017	Peeveelvirus SA7	Peeveelvirus	Caudoviricetes
MK075002.1	Staphylococcus phage phiSP38-1	NA	43851	*Staphylococcus pseudintermedius*	The Rockefeller University, Laboratoy of Bacterial Pathogenesis and Immunology	12/04/2019	Staphylococcus phage phiSP38-1	Peeveelvirus	Caudoviricetes

Accession	Organism_Name	Country	Length (bp)	Host	Organization	Release_Date	Species	Genus	Family or Class
EF462197.1	Staphylococcus phage tp310-1	NA	42232	*Staphylococcus sp.*	University of Regensburg, Institute of Medical Microbiology and Hygiene	13/08/2007	Peeveelvirus tp3101	Peeveelvirus	Caudoviricetes
EF462199.1	Staphylococcus phage tp310-3	NA	42973	*Staphylococcus sp.*	University of Regensburg, Institute of Medical Microbiology and Hygiene	13/08/2007	Staphylococcus phage tp310-3	Peeveelvirus	Caudoviricetes
MH401414.1	Staphylococcus phage SA45ruMSSAST97	Russia	43454	*Staphylococcus aureus*	N.F. Gamaleya National Research Center for Epidemiology and Microbiology Ministry of Health of Russia, Laboratory of Molecular Bases of Pathogenicity	25/07/2018	Staphylococcus phage SA45ruMSSAST97	Peeveelvirus	Caudoviricetes
MH401415.1	Staphylococcus phage SA537ruMSSAST97	Russia	43458	*Staphylococcus aureus*	N.F. Gamaleya National Research Center for Epidemiology and Microbiology Ministry of Health of Russia, Laboratory of Molecular Bases of Pathogenicity	25/07/2018	Staphylococcus phage SA537ruMSSAST97	Peeveelvirus	Caudoviricetes
MH384260.1	Staphylococcus phage SA780ruMSSAST101	Russia	43184	*Staphylococcus aureus*	N.F. Gamaleya National Research Center for Epidemiology and Microbiology Ministry of Health of Russia, Laboratory of Molecular Bases of Pathogenicity	18/09/2018	Peeveelvirus SA780ruMSSAST101	Peeveelvirus	Caudoviricetes
KX827371.1	Staphylococcus phage SpT99F3	United Kingdom	40751	*Staphylococcus pseudintermedius*	University of Warwick, School of Life Sciences	23/11/2016	Staphylococcus phage SpT99F3	Peeveelvirus	Caudoviricetes
OM439663.1	Staphylococcus phage vB_SauS_287	Argentina	43134	*Staphylococcus aureus*	Facultad de Ciencias Medicas, Universidad Nacional de Rosario,	12/02/2022	Staphylococcus phage vB_SauS_287	Phietavirus	Caudoviricetes

Accession	Organism_Name	Country	Length (bp)	Host	Organization	Release_Date	Species	Genus	Family or Class
					Laboratorio de Microbiologia Molecular				
OM439669.1	Staphylococcus phage vB_SauS_760	Argentina	42788	*Staphylococcus aureus*	Facultad de Ciencias Medicas, Universidad Nacional de Rosario, Laboratorio de Microbiologia Molecular	12/02/2022	Staphylococcus phage vB_SauS_760	Phietavirus	Caudoviricetes
OM439672.1	Staphylococcus phage vB_SauS_I73	Argentina	43575	*Staphylococcus aureus*	Facultad de Ciencias Medicas, Universidad Nacional de Rosario, Laboratorio de Microbiologia Molecular	12/02/2022	Staphylococcus phage vB_SauS_I73	Phietavirus	Caudoviricetes
OM439673.1	Staphylococcus phage vB_SauS_Mh1	Argentina	43800	*Staphylococcus aureus*	Facultad de Ciencias Medicas, Universidad Nacional de Rosario, Laboratorio de Microbiologia Molecular	12/02/2022	Staphylococcus phage vB_SauS_Mh1	Phietavirus	Caudoviricetes
OM439674.1	Staphylococcus phage vB_SauS_Mh15	Argentina	43090	*Staphylococcus aureus*	Facultad de Ciencias Medicas, Universidad Nacional de Rosario, Laboratorio de Microbiologia Molecular	12/02/2022	Staphylococcus phage vB_SauS_Mh15	Phietavirus	Caudoviricetes
CP071049.1	Staphylococcus phage WBG8381	Australia	42493	*Staphylococcus aureus*	Curtin University, School of Pharmacy & Biomedical Sciences	03/03/2021	Staphylococcus phage WBG8381	Phietavirus	Caudoviricetes
MK211557.1	Staphylococcus phage Henu2	China	43513	*Staphylococcus aureus*	Medical College of Henan University, Institute of Medical Biological Information	30/01/2019	Phietavirus Henu2	Phietavirus	Caudoviricetes
MW238767.1	Staphylococcus phage IME1364_02	China	42338	*Staphylococcus aureus*	Beijing University of Chemical	13/12/2020	Staphylococcus phage IME1364_02	Phietavirus	Caudoviricetes

Accession	Organism_Name	Country	Length (bp)	Host	Organization	Release_Date	Species	Genus	Family or Class
					Technology, College of Life Science and Technology				
MK290764.1	Staphylococcus phage B122	Czech Republic	43108	*Staphylococcus aureus*	Masaryk University, Faculty of Science, Department of Experimental Biology	13/01/2019	Phietavirus B122	Phietavirus	Caudoviricetes
KP893289.1	Staphylococcus phage B166	Czech Republic	42881	*Staphylococcus aureus*	Masaryk University, Faculty of Science, Department of Experimental Biology	17/04/2015	Phietavirus B166	Phietavirus	Caudoviricetes
KP893290.1	Staphylococcus phage B236	Czech Republic	43228	*Staphylococcus aureus*	Masaryk University, Faculty of Science, Department of Experimental Biology	17/04/2015	Phietavirus B236	Phietavirus	Caudoviricetes
KT344895.1	Staphylococcus phage phiJB	Czech Republic	43012	*Staphylococcus aureus*	Masaryk University, Department of Experimental Biology	29/10/2015	Phietavirus JB	Phietavirus	Caudoviricetes
JX013863.1	Staphylococcus phage StauST398-1	France	45242	*Staphylococcus aureus*	University of Geneva Hospitals, Genomic Research Laboratory	30/04/2013	Phietavirus StauST3981	Phietavirus	Caudoviricetes
JQ973847.1	Staphylococcus phage StauST398-3	France	41392	*Staphylococcus aureus*	University of Geneva Hospitals, Genomic Research Laboratory	30/04/2013	Phietavirus StauST3983	Phietavirus	Caudoviricetes
KJ140076.1	Staphylococcus phage DW2	Ireland	41941	*Staphylococcus aureus*	Cork Institute of Technology, Biological Sciences	03/06/2014	Staphylococcus phage DW2	Phietavirus	Caudoviricetes
MG564297.1	Staphylococcus phage UPMK_2	Malaysia	40955	*Staphylococcus sp.*	Universiti Putra Malaysia, Microbiology, Faculty of Biotechnology and Biomelcular Sciences	10/01/2018	Phietavirus UPMK2	Phietavirus	Caudoviricetes
KR709302.1	Staphylococcus phage 55-2	NA	41898	*Staphylococcus aureus*	College of Medical, Veterinary and Life	05/07/2015	Phietavirus pv55	Phietavirus	Caudoviricetes

Accession	Organism_Name	Country	Length (bp)	Host	Organization	Release_Date	Species	Genus	Family or Class
					Sciences, Institute of Infection, Immunity and Inflammation				
KR709303.1	Staphylococcus phage 55-3	NA	42309	*Staphylococcus aureus*	College of Medical, Veterinary and Life Sciences, Institute of Infection, Immunity and Inflammation	05/07/2015	Phietavirus pv55	Phietavirus	Caudoviricetes
CP062422.1	Staphylococcus phage ECel-2020l	NA	43478	*Staphylococcus aureus*	University of Central Florida, Burnett School of Biomedical Sciences	07/03/2022	Staphylococcus phage ECel-2020l	Phietavirus	Caudoviricetes
CP062462.1	Staphylococcus phage ECel-2020q	NA	42844	*Staphylococcus aureus*	University of central Florida, Burnett School of Biomedical Sciences	07/03/2022	Staphylococcus phage ECel-2020q	Phietavirus	Caudoviricetes
AP001553.1	Staphylococcus phage phiETA	NA	43081	*Staphylococcus aureus*	Microbiology; Kasumi 1-2-3, Hiroshima, Hiroshima 734-8553	04/07/2000	Phietavirus ETA	Phietavirus	Caudoviricetes
AP008954.1	Staphylococcus phage phiETA3	NA	43282	*Staphylococcus aureus*	Graduate School of Biomedical Sciences Dept. Bacteriology; Kasumi 1-2-3, Hiroshima, Hiroshima 734-8553	06/01/2007	Phietavirus ETA3	Phietavirus	Caudoviricetes
AB370268.1	Staphylococcus phage phiMR11	NA	43011	*Staphylococcus aureus*	Medical School; Kohasu, Oko-cho, Nankoku, Kochi 783-8505	07/12/2007	Phietavirus MR11	Phietavirus	Caudoviricetes
KP209285.1	Staphylococcus phage phiNM4-gamma4	NA	40365	*Staphylococcus aureus*	The Rockefeller University, Bacteriology	18/01/2015	Phietavirus NM4	Phietavirus	Caudoviricetes
JX094501.1	Staphylococcus phage SA13	NA	42652	*Staphylococcus aureus*	Seoul National University, Department of Food and Animal Biotechnology	31/05/2013	Phietavirus SA13	Phietavirus	Caudoviricetes
KC595279.1	Staphylococcus phage StauST398-5	NA	43301	*Staphylococcus aureus*	Geneva University Hospitals,	11/02/2014	Phietavirus StauST3985	Phietavirus	Caudoviricetes

Accession	Organism_Name	Country	Length (bp)	Host	Organization	Release_Date	Species	Genus	Family or Class
					Genomic Research Laboratory				
ON571632.1	Staphylococcus phage Sushi	NA	43755	*Staphylococcus aureus*	Imperial College London, MRC CMBI	27/06/2022	Staphylococcus phage Sushi	Phietavirus	Caudoviricetes
MN904510.1	Staphylococcus phage vB_SauS-SAP27	NA	43444	*Staphylococcus aureus*	Gachon University, Department of Foodbiotechnology	16/02/2020	Phietavirus SAP27	Phietavirus	Caudoviricetes
AY954950.1	Staphylococcus phage 187	NA	39620	*Staphylococcus sp.*	Targanta Therapeutics	05/04/2005	Phietavirus pv187	Phietavirus	Caudoviricetes
AY954964.1	Staphylococcus phage 29	NA	42802	*Staphylococcus sp.*	Targanta Therapeutics	05/04/2005	Phietavirus pv29	Phietavirus	Caudoviricetes
AY954958.1	Staphylococcus phage 37	NA	43681	*Staphylococcus sp.*	Targanta Therapeutics	05/04/2005	Phietavirus pv37	Phietavirus	Caudoviricetes
AY954965.1	Staphylococcus phage 52A	NA	41690	*Staphylococcus sp.*	Targanta Therapeutics	05/04/2005	Phietavirus pv52a	Phietavirus	Caudoviricetes
AY954963.1	Staphylococcus phage 55	NA	41902	*Staphylococcus sp.*	Targanta Therapeutics	05/04/2005	Phietavirus pv55	Phietavirus	Caudoviricetes
AY954962.1	Staphylococcus phage 71	NA	43114	*Staphylococcus sp.*	Targanta Therapeutics	05/04/2005	Phietavirus pv71	Phietavirus	Caudoviricetes
DQ908929.1	Staphylococcus phage 80	NA	42140	*Staphylococcus sp.*	Virginia Commonwealth University, Microbiology and Immunology	31/08/2007	Phietavirus pv80	Phietavirus	Caudoviricetes
AY954966.1	Staphylococcus phage 88	NA	43231	*Staphylococcus sp.*	Targanta Therapeutics	05/04/2005	Phietavirus pv88	Phietavirus	Caudoviricetes
AY954967.1	Staphylococcus phage 92	NA	42431	*Staphylococcus sp.*	Targanta Therapeutics	05/04/2005	Phietavirus pv92	Phietavirus	Caudoviricetes
AY954960.1	Staphylococcus phage 96	NA	43576	*Staphylococcus sp.*	Targanta Therapeutics	05/04/2005	Phietavirus pv96	Phietavirus	Caudoviricetes
AY954959.1	Staphylococcus phage EW	NA	45286	*Staphylococcus sp.*	Targanta Therapeutics	05/04/2005	Phietavirus EW	Phietavirus	Caudoviricetes
DQ530362.1	Staphylococcus phage phinm4	NA	43189	*Staphylococcus sp.*	University of Chicago, Microbiology	31/10/2006	Phietavirus NM4	Phietavirus	Caudoviricetes
AY954961.1	Staphylococcus phage ROSA	NA	43155	*Staphylococcus sp.*	Targanta Therapeutics	05/04/2005	Phietavirus ROSA	Phietavirus	Caudoviricetes
AY954968.1	Staphylococcus phage X2	NA	43440	*Staphylococcus sp.*	Targanta Therapeutics	05/04/2005	Phietavirus X2	Phietavirus	Caudoviricetes
KJ452291.1	Staphylococcus phage 3MRA	USA	41931	*Staphylococcus aureus*	UCSD, Pathology	13/07/2015	Phietavirus pv3MRA	Phietavirus	Caudoviricetes
MK618716.1	Staphylococcus phage Sebago	USA	43878	*Staphylococcus aureus*	Texas A&M University, Center for Phage Technology	02/04/2019	Phietavirus sebago	Phietavirus	Caudoviricetes

Accession	Organism_Name	Country	Length (bp)	Host	Organization	Release_Date	Species	Genus	Family or Class
MW364975.1	Staphylococcus virus vB_SepS_E72	Germany	44592	*Staphylococcus epidermidis*	Faculty of Science, Masaryk University, Department of Experimental Biology	27/01/2021	Staphylococcus virus vB_SepS_E72	Rockefellervirus	Caudoviricetes
KU598975.1	Staphylococcus phage CNPx	NA	43293	*Staphylococcus epidermidis*	Institut Pasteur, Synthetic Biology Group, Microbiology Department	31/03/2016	Rockefellervirus CNPx	Rockefellervirus	Caudoviricetes
KY653120.1	Staphylococcus phage IME1348_01	NA	42371	*Staphylococcus epidermidis*	Beijing Institute of Microbiology and Epidemiology, State Key Laboratory of Pathogen	06/05/2017	Rockefellervirus IME134801	Rockefellervirus	Caudoviricetes
JN192400.1	Staphylococcus phage Ipla5	NA	43581	*Staphylococcus epidermidis*	IPLA-CSIC, Technology and Biotechnology of Dairy Products	24/07/2012	Rockefellervirus IPLA5	Rockefellervirus	Caudoviricetes
JN192401.1	Staphylococcus phage Ipla7	NA	42123	*Staphylococcus epidermidis*	IPLA-CSIC, Technology and Biotechnology of Dairy Products	24/07/2012	Rockefellervirus IPLA7	Rockefellervirus	Caudoviricetes
KY653129.1	Staphylococcus phage IME1365_01	NA	44875	*Staphylococcus haemolyticus*	Beijing Institute of Microbiology and Epidemiology, State Key Laboratory of Pathogen	06/05/2017	Staphylococcus phage IME1365_01	Rockefellervirus	Caudoviricetes
DQ831957.1	Staphylococcus phage CNPH82	NA	43420	*Staphylococcus sp.*	The Rockefeller University, Laboratory of Bacterial Pathogenesis and Immunology	04/12/2006	Rockefellervirus CNPH82	Rockefellervirus	Caudoviricetes
DQ834250.1	Staphylococcus phage PH15	NA	44041	*Staphylococcus sp.*	The Rockefeller University, Laboratory of Bacterial Pathogenesis and Immunology	04/12/2006	Rockefellervirus PH15	Rockefellervirus	Caudoviricetes
MW221967.1	Staphylococcus phage PMBT9	NA	41623	*Staphylococcus sp.*	Max Rubner-Instiut, Microbiology and Biotechnology	27/01/2021	Staphylococcus phage PMBT9	Rockefellervirus	Caudoviricetes

Accession	Organism_Name	Country	Length (bp)	Host	Organization	Release_Date	Species	Genus	Family or Class
MW364973.1	Staphylococcus phage vB_SepS_456	Netherlands	43266	*Staphylococcus epidermidis*	Faculty of Science, Masaryk University, Department of Experimental Biology	27/01/2021	Rockefellervirus CNPH82	Rockefellervirus	Caudoviricetes
MW364974.1	Staphylococcus virus vB_SepS_459	Netherlands	42498	*Staphylococcus epidermidis*	Faculty of Science, Masaryk University, Department of Experimental Biology	27/01/2021	Staphylococcus virus vB_SepS_459	Rockefellervirus	Caudoviricetes
MW364972.1	Staphylococcus virus vB_SepS_48	Netherlands	42460	*Staphylococcus epidermidis*	Faculty of Science, Masaryk University, Department of Experimental Biology	27/01/2021	Staphylococcus virus vB_SepS_48	Rockefellervirus	Caudoviricetes
MZ417349.1	Staphylococcus phage PG-2021_89	Switzerland	43039	*Staphylococcus epidermidis*	Institue of Marine Sciences (ICM) CSIC, Department of Marine Biology and Oceanography	24/09/2021	Staphylococcus phage PG-2021_89	Rockefellervirus	Caudoviricetes
MZ417351.1	Staphylococcus phage PG-2021_90	Switzerland	44493	*Staphylococcus epidermidis*	Institue of Marine Sciences (ICM) CSIC, Department of Marine Biology and Oceanography	24/09/2021	Staphylococcus phage PG-2021_90	Rockefellervirus	Caudoviricetes
MZ417352.1	Staphylococcus phage PG-2021_91	Switzerland	42188	*Staphylococcus epidermidis*	Institue of Marine Sciences (ICM) CSIC, Department of Marine Biology and Oceanography	24/09/2021	Staphylococcus phage PG-2021_91	Rockefellervirus	Caudoviricetes
MZ417353.1	Staphylococcus phage PG-2021_93	Switzerland	43459	*Staphylococcus epidermidis*	Institue of Marine Sciences (ICM) CSIC, Department of Marine Biology and Oceanography	24/09/2021	Staphylococcus phage PG-2021_93	Rockefellervirus	Caudoviricetes
MT596498.1	Staphylococcus phage vB_SepS_BE01	Switzerland	42718	*Staphylococcus epidermidis*	Inselspital, Intensive Care Medicine / DBMR	28/06/2021	Staphylococcus phage vB_SepS_BE01	Rockefellervirus	Caudoviricetes
MT596497.1	Staphylococcus phage 456	United Kingdom	43393	*Staphylococcus epidermidis*	Inselspital, Intensive Care Medicine / DBMR	28/06/2021	Rockefellervirus CNPH82	Rockefellervirus	Caudoviricetes
MW364971.1	Staphylococcus virus vB_SepS_27	United Kingdom	42935	*Staphylococcus epidermidis*	Faculty of Science, Masaryk University,	27/01/2021	Staphylococcus virus vB_SepS_27	Rockefellervirus	Caudoviricetes

Accession	Organism_Name	Country	Length (bp)	Host	Organization	Release_Date	Species	Genus	Family or Class
					Department of Experimental Biology				
KM366100.1	Staphylococcus phage BP39	Canada	17641	*Staphylococcus aureus*	Biophage Pharma Inc.	03/07/2016	Rosenblumvirus BP39	Rosenblumvirus	Rountreeviridae
MZ359091.1	Staphylococcus phage JPL-50	China	16927	*Staphylococcus aureus*	Qingdao Agricultural University, Veterinary Medicine	29/06/2021	Staphylococcus phage JPL-50	Rosenblumvirus	Rountreeviridae
KU992911.1	Staphylococcus phage SLPW	China	17861	*Staphylococcus aureus*	Shanghai Jiao Tong University, School of Agriculture and Biology	02/06/2016	Rosenblumvirus SLPW	Rosenblumvirus	Rountreeviridae
LC709172.1	Staphylococcus phage vB_SauP_JS26	China	17226	*Staphylococcus aureus*	Institute of Food Safety and Nutrition; No.50 Zhongling Street, Nanjing, Jiangsu 210014	12/05/2022	Staphylococcus phage vB_SauP_JS26	Rosenblumvirus	Rountreeviridae
NC_055906.1	Staphylococcus phage vB_SauP_EBHT	Germany	17471	*Staphylococcus aureus*	National Center for Biotechnology Information, NIH	02/06/2021	Rosenblumvirus PSa3	Rosenblumvirus	Rountreeviridae
MK936475.1	Staphylococcus phage SA03-CTH2	Japan	17511	*Staphylococcus aureus*	Kyushu University, Graduate School of Agriculture, Department of Bioscience and Biotechnology	31/05/2020	Staphylococcus phage SA03-CTH2	Rosenblumvirus	Rountreeviridae
MK922546.1	Staphylococcus phage SA1-CTA1	Japan	17527	*Staphylococcus aureus*	Kyushu University, Graduate School of Agriculture, Department of Bioscience and Biotechnology	31/05/2020	Staphylococcus phage SA1-CTA1	Rosenblumvirus	Rountreeviridae
MK922547.1	Staphylococcus phage SA30-CTH2	Japan	17545	*Staphylococcus aureus*	Kyushu University, Graduate School of Agriculture, Department of Bioscience and Biotechnology	31/05/2020	Staphylococcus phage SA30-CTH2	Rosenblumvirus	Rountreeviridae
MK903033.1	Staphylococcus phage SA44-CTH7	Japan	17522	*Staphylococcus aureus*	Kyushu University, Graduate School of Agriculture, Department of	10/05/2020	Staphylococcus phage SA44-CTH7	Rosenblumvirus	Rountreeviridae

Accession	Organism_Name	Country	Length (bp)	Host	Organization	Release_Date	Species	Genus	Family or Class
					Bioscience and Biotechnology				
MK936476.1	Staphylococcus phage SA46-CL1	Japan	17508	*Staphylococcus aureus*	Kyushu University, Graduate School of Agriculture, Department of Bioscience and Biotechnology	31/05/2020	Rosenblumvirus SA46CL1	Rosenblumvirus	Rountreeviridae
MK764384.1	Staphylococcus phage SA46-CTH2	Japan	17505	*Staphylococcus aureus*	Kyushu University, Bioscience and Biotechnology, Graduate School of Agriculture	07/01/2020	Staphylococcus phage SA46-CTH2	Rosenblumvirus	Rountreeviridae
MK922548.1	Staphylococcus phage SA46-CTH4	Japan	17473	*Staphylococcus aureus*	Kyushu University, Graduate School of Agriculture, Department of Bioscience and Biotechnology	31/05/2020	Staphylococcus phage SA46-CTH4	Rosenblumvirus	Rountreeviridae
KJ210330.1	Staphylococcus phage GRCS	NA	17869	*Staphylococcus aureus*	Institute for Bioscience and Biotechnology Research	19/02/2014	Rosenblumvirus GRCS	Rosenblumvirus	Rountreeviridae
MW363799.1	Staphylococcus phage LSA2366	NA	17056	*Staphylococcus aureus*	School of Food, Sichuan Agricultural University	17/01/2021	Rosenblumvirus LSA2366	Rosenblumvirus	Rountreeviridae
AB626963.1	Staphylococcus phage S13'	NA	18186	*Staphylococcus aureus*	Department of Microbiology and Infection; Okou-cho kohasu, Nankoku-City, Kochi 783-8505	22/12/2011	Rosenblumvirus S241	Rosenblumvirus	Rountreeviridae
AB626962.1	Staphylococcus phage S24-1	NA	18168	*Staphylococcus aureus*	Department of Microbiology and Infection; Okou-cho kohasu, Nankoku-City, Kochi 783-8505	22/12/2011	Rosenblumvirus S241	Rosenblumvirus	Rountreeviridae
KY000084.1	Staphylococcus phage SCH1	NA	18023	*Staphylococcus aureus*	BPhage LLC	23/11/2016	Rosenblumvirus SCH1	Rosenblumvirus	Rountreeviridae
KY000085.1	Staphylococcus phage SCH111	NA	18018	*Staphylococcus aureus*	BPhage LLC	23/11/2016	Rosenblumvirus SCH1	Rosenblumvirus	Rountreeviridae
MG766218.1	Staphylococcus phage vB_SauP_phiAGO1.3	NA	17603	*Staphylococcus aureus*	Institute of Biochemistry and Biophysics of the Polish Academy of	24/01/2018	Rosenblumvirus AGO13	Rosenblumvirus	Rountreeviridae

Accession	Organism_Name	Country	Length (bp)	Host	Organization	Release_Date	Species	Genus	Family or Class
					Sciences, Department of Microbial Biochemistry				
MG766219.2	Staphylococcus phage vB_SauP_phiAGO1.9	NA	17637	*Staphylococcus aureus*	Institute of Biochemistry and Biophysics of the Polish Academy of Sciences, Department of Microbial Biochemistry	24/01/2018	Rosenblumvirus AGO13	Rosenblumvirus	Rountreeviridae
AY954949.1	Staphylococcus phage 66	NA	18199	*Staphylococcus sp.*	Targanta Therapeutics	05/04/2005	Rosenblumvirus rv66	Rosenblumvirus	Rountreeviridae
AF513033.1	Staphylococcus phage P68	NA	18227	*Staphylococcus sp.*	University of Vienna, Inst. of Microbiology and Genetics	31/03/2003	Staphylococcus phage P68	Rosenblumvirus	Rountreeviridae
AF513032.1	Staphylococcus phage phi44AHJD	NA	16784	*Staphylococcus sp.*	University of Vienna, Inst. of Microbiology and Genetics	31/03/2003	Rosenblumvirus rv44AHJD	Rosenblumvirus	Rountreeviridae
MW286254.1	Staphylococcus phage TSP	Pakistan	17987	*Staphylococcus aureus*	University of the Punjab, Microbiology and Molecular Genetics	31/12/2021	Rosenblumvirus SCH1	Rosenblumvirus	Rountreeviridae
MN150710.1	Staphylococcus phage vB_SauP-436A1	Russia	18028	*Staphylococcus aureus*	Research Institute of Physical-Chemical Medicine, Molecular Genetics of Microorganisms	14/08/2019	Rosenblumvirus SCH1	Rosenblumvirus	Rountreeviridae
MH107118.1	Staphylococcus phage CSA13	South Korea	17034	*Staphylococcus aureus*	Seoul National University, Food Biotechnology, College of Agriculture and Life Sciences	20/02/2019	Rosenblumvirus CSA13	Rosenblumvirus	Rountreeviridae
EU136189.1	Staphylococcus phage SAP-2	South Korea	17938	*Staphylococcus aureus*	Intron Biotechnology, Inc., Institute of Intron	23/09/2007	Rosenblumvirus SAP2	Rosenblumvirus	Rountreeviridae
MH972260.1	Staphylococcus phage Pabna	USA	17700	*Staphylococcus aureus*	University of Alabama, Biological Sciences	04/12/2018	Rosenblumvirus pabna	Rosenblumvirus	Rountreeviridae
MN098325.1	Staphylococcus phage Portland	USA	17711	*Staphylococcus aureus*	TAMU, Department of Biochemistry	09/10/2019	Rosenblumvirus EBHT	Rosenblumvirus	Rountreeviridae

Accession	Organism_Name	Country	Length (bp)	Host	Organization	Release_Date	Species	Genus	Family or Class
					and Biophysics, Center for Phage Technology				
KX171212.1	Staphylococcus phage vB_SscM-1	NA	139681	*Staphylococcus sp.*	Institute of Biochemistry and Biophysics, Polish Academy of Sciences, Laboratory of Molecular Biology (affiliated with the University of Gdansk)	13/05/2017	Sciuriunavirus SscM1	Sciuriunavirus	Herelleviridae
KX171213.1	Staphylococcus phage vB_SscM-2	NA	139682	*Staphylococcus sp.*	Institute of Biochemistry and Biophysics, Polish Academy of Sciences, Laboratory of Molecular Biology (affiliated with the University of Gdansk)	13/05/2017	Sciuriunavirus SscM1	Sciuriunavirus	Herelleviridae
KP027447.1	Staphylococcus phage phiIPLA-C1C	NA	140961	*Staphylococcus sp.*	IPLA-CSIC, Technology and Biotechnology of Dairy Products	23/03/2015	Sepunavirus IPLAC1C	Sepunavirus	Herelleviridae
KF021268.1	Staphylococcus phage phiIBB-SEP1	Portugal	139928	*Staphylococcus epidermidis*	IBB Institute of Biotechnology and Bioengineering, Center of Biological Engineering, University of Minho	10/10/2013	Sepunavirus SEP1	Sepunavirus	Herelleviridae
MZ417323.1	Staphylococcus phage PG-2021_1	Switzerland	143764	*Staphylococcus epidermidis*	Institue of Marine Sciences (ICM) CSIC, Department of Marine Biology and Oceanography	24/09/2021	Staphylococcus phage PG-2021_1	Sepunavirus	Herelleviridae
MT596501.1	Staphylococcus phage vB_SepM_BE04	Switzerland	142331	*Staphylococcus epidermidis*	Inselspital, Intensive Care Medicine / DBMR	28/06/2021	Staphylococcus phage vB_SepM_BE04	Sepunavirus	Herelleviridae
MT596502.1	Staphylococcus phage vB_SepM_BE05	Switzerland	140271	*Staphylococcus epidermidis*	Inselspital, Intensive Care Medicine / DBMR	28/06/2021	Staphylococcus phage vB_SepM_BE05	Sepunavirus	Herelleviridae

Accession	Organism_Name	Country	Length (bp)	Host	Organization	Release_Date	Species	Genus	Family or Class
MT596503.1	Staphylococcus phage vB_SepM_BE06	Switzerland	140659	*Staphylococcus epidermidis*	Inselspital, Intensive Care Medicine / DBMR	28/06/2021	Staphylococcus phage vB_SepM_BE06	Sepunavirus	Herelleviridae
MT596504.1	Staphylococcus phage vB_SepM_BE07	Switzerland	140661	*Staphylococcus epidermidis*	Inselspital, Intensive Care Medicine / DBMR	28/06/2021	Staphylococcus phage vB_SepM_BE07	Sepunavirus	Herelleviridae
MT596505.1	Staphylococcus phage vB_SepM_BE08	Switzerland	140659	*Staphylococcus epidermidis*	Inselspital, Intensive Care Medicine / DBMR	28/06/2021	Staphylococcus phage vB_SepM_BE08	Sepunavirus	Herelleviridae
MT596506.1	Staphylococcus phage vB_SepM_BE09	Switzerland	140668	*Staphylococcus epidermidis*	Inselspital, Intensive Care Medicine / DBMR	28/06/2021	Staphylococcus phage vB_SepM_BE09	Sepunavirus	Herelleviridae
MH321490.1	Staphylococcus phage Quidividi	USA	141446	*Staphylococcus epidermidis*	Texas A&M University, Center for Phage Technology, Department of Biochemistry and Biophysics	25/07/2018	Staphylococcus phage Quidividi	Sepunavirus	Herelleviridae
MH542234.1	Staphylococcus phage Terranova	USA	141288	*Staphylococcus epidermidis*	TAMU, Department of Biochemistry and Biophysics, Center for Phage Technology	18/09/2018	Staphylococcus phage Terranova	Sepunavirus	Herelleviridae
MH321491.1	Staphylococcus phage Twillingate	USA	142592	*Staphylococcus epidermidis*	Texas A&M University, Center for Phage Technology, Department of Biochemistry and Biophysics	25/07/2018	Staphylococcus phage Twillingate	Sepunavirus	Herelleviridae
MK893987.1	Staphylococcus phage PMBT8	Germany	88129	*Staphylococcus hyicus*	Max Rubner-Institut, Department of Microbiology and Biotechnology	29/06/2019	Staphylococcus phage PMBT8	Sextaecvirus	Caudoviricetes
MH356730.1	Staphylococcus phage VB-SauS-SA2	NA	89055	*Staphylococcus aureus*	Qingdao Agricultural University, College of Veterinary Medicine	07/07/2018	Staphylococcus phage VB-SauS-SA2	Sextaecvirus	Caudoviricetes
KF929199.1	Staphylococcus phage vB_SepS_SEP9	Portugal	92417	*Staphylococcus epidermidis*	IBB Institute of Biotechnology and Bioengineering, Center of Biological	21/02/2014	Sextaecvirus SEP9	Sextaecvirus	Caudoviricetes

Accession	Organism_Name	Country	Length (bp)	Host	Organization	Release_Date	Species	Genus	Family or Class
					Engineering, University of Minho				
MZ417336.1	Staphylococcus phage PG-2021_46	Switzerland	86018	*Staphylococcus epidermidis*	Institue of Marine Sciences (ICM) CSIC, Department of Marine Biology and Oceanography	24/09/2021	Staphylococcus phage PG-2021_46	Sextaecvirus	Caudoviricetes
MZ417339.1	Staphylococcus phage PG-2021_5	Switzerland	92222	*Staphylococcus epidermidis*	Institue of Marine Sciences (ICM) CSIC, Department of Marine Biology and Oceanography	24/09/2021	Staphylococcus phage PG-2021_5	Sextaecvirus	Caudoviricetes
MZ417343.1	Staphylococcus phage PG-2021_74	Switzerland	85762	*Staphylococcus epidermidis*	Institue of Marine Sciences (ICM) CSIC, Department of Marine Biology and Oceanography	24/09/2021	Staphylococcus phage PG-2021_74	Sextaecvirus	Caudoviricetes
MZ417348.1	Staphylococcus phage PG-2021_88	Switzerland	92222	*Staphylococcus epidermidis*	Institue of Marine Sciences (ICM) CSIC, Department of Marine Biology and Oceanography	24/09/2021	Staphylococcus phage PG-2021_88	Sextaecvirus	Caudoviricetes
MT596499.1	Staphylococcus phage vB_SepS_BE02	Switzerland	95233	*Staphylococcus epidermidis*	Inselspital, Intensive Care Medicine / DBMR	28/06/2021	Staphylococcus phage vB_SepS_BE02	Sextaecvirus	Caudoviricetes
OM373549.1	Staphylococcus phage vB_SpsS-DH5	USA	92077	*Staphylococcus pseudintermedius*	Baylor College of Medicine, Molecular Virology and Microbiology	30/03/2022	Staphylococcus phage vB_SpsS-DH5	Sextaecvirus	Caudoviricetes
OP263969.1	Staphylococcus phage Koomba-kaat_1	Australia	135469	*Staphylococcus aureus*	Telethon Kids Institute, Epithelial	26/09/2022	Staphylococcus phage Koomba-kaat_1	Silviavirus	Herelleviridae
ON866946.1	Staphylococcus phage LSA2311	China	144592	*Staphylococcus aureus*	Huazhong Agricultural University, College of Food Science and Technology	10/07/2022	Staphylococcus phage LSA2311	Silviavirus	Herelleviridae
KY779848.1	Staphylococcus phage qdsa001	China	135563	*Staphylococcus aureus*	Ocean University of China, Food Science and Engineering	20/05/2017	Staphylococcus phage qdsa001	Silviavirus	Herelleviridae
OQ025230.1	Staphylococcus phage SAP6	China	144705	*Staphylococcus aureus*	Army Medical University	25/12/2022	Staphylococcus phage SAP6	Silviavirus	Herelleviridae
ON814135.1	Staphylococcus phage vB_SauM-V1SA20	France	136866	*Staphylococcus aureus*	Hospices Civils de Lyon	03/09/2022	Staphylococcus phage vB_SauM-V1SA20	Silviavirus	Herelleviridae
AP019522.1	Staphylococcus phage MR003	Japan	132152	*Staphylococcus aureus*	Department of Bioengineering, School of Life	14/03/2019	Staphylococcus phage MR003	Silviavirus	Herelleviridae

Accession	Organism_Name	Country	Length (bp)	Host	Organization	Release_Date	Species	Genus	Family or Class
					Science and Technology; 4259 J2-15 Nagatsuta-cho, Midori-ku, Yokohama 226-8501				
MW363798.1	Staphylococcus phage LSA2308	NA	144592	*Staphylococcus aureus*	School of Food, Sichuan Agricultural University	17/01/2021	Staphylococcus phage LSA2308	Silviavirus	Herelleviridae
OP856857.1	Staphylococcus phage PBSA08	NA	136673	*Staphylococcus aureus*	Kookmin University, Department of Food Bioconvergence Lab	30/11/2022	Staphylococcus phage PBSA08	Silviavirus	Herelleviridae
JX846612.1	Staphylococcus phage vB_SauM_Remus	NA	134643	*Staphylococcus aureus*	University of Leuven, Biosystems	15/08/2013	Silviavirus remus	Silviavirus	Herelleviridae
JX846613.1	Staphylococcus phage vB_SauM_Romulus	NA	131332	*Staphylococcus aureus*	University of Leuven, Biosystems	12/03/2013	Silviavirus remus	Silviavirus	Herelleviridae
MW546071.1	Staphylococcus phage PM56	NA	136656	*Staphylococcus sp.*	PhagoMed BioPharma GmbH, Bacteriophage Dept.	16/05/2021	Silviavirus remus	Silviavirus	Herelleviridae
MW546075.1	Staphylococcus phage PM93	NA	144038	*Staphylococcus sp.*	PhagoMed BioPharma GmbH, Bacteriophage Dept.	16/05/2021	Staphylococcus phage PM93	Silviavirus	Herelleviridae
MW546077.1	Staphylococcus phage Romulus	NA	136651	*Staphylococcus sp.*	PhagoMed BioPharma GmbH, Bacteriophage Dept.	16/05/2021	Silviavirus remus	Silviavirus	Herelleviridae
JX194239.1	Staphylococcus phage SA11	South Korea	136326	*Staphylococcus aureus*	Hankuk University of Foreign Studies, Bioscience and Biotechnology	01/08/2012	Silviavirus SA11	Silviavirus	Herelleviridae
KX532239.1	Staphylococcus phage StAP1	South Korea	135502	*Staphylococcus aureus*	Chonbuk National University, Food Science and Technology	18/01/2017	Staphylococcus phage StAP1	Silviavirus	Herelleviridae
KP881332.1	Staphylococcus phage Stau2	Taiwan	133798	*Staphylococcus aureus*	Central Taiwan University of Science and Technology, Department of	05/01/2016	Silviavirus stau2	Silviavirus	Herelleviridae

Accession	Organism_Name	Country	Length (bp)	Host	Organization	Release_Date	Species	Genus	Family or Class
					Medical Laboratory Science and Biotechnology				
MZ643272.1	Staphylococcus phage vB_Sau-RP15	Thailand	139486	*Staphylococcus aureus*	Silpakorn University, Department of Biotechnology, Faculty of Engineering and Industrial Technology	03/08/2021	Staphylococcus phage vB_Sau-RP15	Silviavirus	Herelleviridae
MT080584.1	Staphylococcus phage vB_SauM_EW15	United Kingdom	135802	*Staphylococcus carnosus*	Manchester Metropolitan University, Department of Life Sciences	07/03/2020	Staphylococcus phage vB_SauM_EW15	Silviavirus	Herelleviridae
MT080587.1	Staphylococcus phage vB_SauM_EW20	United Kingdom	135784	*Staphylococcus carnosus*	Manchester Metropolitan University, Department of Life Sciences	07/03/2020	Staphylococcus phage vB_SauM_EW20	Silviavirus	Herelleviridae
MT080588.1	Staphylococcus phage vB_SauM_EW22	United Kingdom	135820	*Staphylococcus carnosus*	Manchester Metropolitan University, Department of Life Sciences	07/03/2020	Staphylococcus phage vB_SauM_EW22	Silviavirus	Herelleviridae
MT080590.1	Staphylococcus phage vB_SauM_EW27	United Kingdom	135801	*Staphylococcus carnosus*	Manchester Metropolitan University, Department of Life Sciences	07/03/2020	Staphylococcus phage vB_SauM_EW27	Silviavirus	Herelleviridae
MT080595.1	Staphylococcus phage vB_SauM_EW41	United Kingdom	132999	*Staphylococcus carnosus*	Manchester Metropolitan University, Department of Life Sciences	07/03/2020	Staphylococcus phage vB_SauM_EW41	Silviavirus	Herelleviridae
OM439665.1	Staphylococcus phage vB_SauS_320	Argentina	45809	*Staphylococcus aureus*	Facultad de Ciencias Medicas, Universidad Nacional de Rosario, Laboratorio de Microbiologia Molecular	12/02/2022	Staphylococcus phage vB_SauS_320	Triavirus	Caudoviricetes
OM439667.1	Staphylococcus phage vB_SauS_690	Argentina	45771	*Staphylococcus aureus*	Facultad de Ciencias Medicas, Universidad	12/02/2022	Staphylococcus phage vB_SauS_690	Triavirus	Caudoviricetes

Accession	Organism_Name	Country	Length (bp)	Host	Organization	Release_Date	Species	Genus	Family or Class
					Nacional de Rosario, Laboratorio de Microbiologia Molecular				
MF580410.1	Staphylococcus phage phiSa2wa_st1	Australia	45585	*Staphylococcus aureus*	Curtin University, School of Biomedical Sciences	28/10/2017	Triavirus st1	Triavirus	Caudoviricetes
MG029518.1	Staphylococcus phage phiSa2wa_st121mssa	Australia	45621	*Staphylococcus aureus*	Curtin University, School of Biomedical Sciences	10/01/2018	Triavirus st121mssa	Triavirus	Caudoviricetes
MG029511.1	Staphylococcus phage phiSa2wa_st30	Australia	45780	*Staphylococcus aureus*	Curtin University, School of Biomedical Sciences	10/01/2018	Triavirus st30	Triavirus	Caudoviricetes
MG029509.1	Staphylococcus phage phiSa2wa_st5	Australia	44823	*Staphylococcus aureus*	Curtin University, School of Biomedical Sciences	10/01/2018	Triavirus st5	Triavirus	Caudoviricetes
MG029513.1	Staphylococcus phage phiSa2wa_st72	Australia	47213	*Staphylococcus aureus*	Curtin University, School of Biomedical Sciences	10/01/2018	Triavirus P240	Triavirus	Caudoviricetes
MG029514.1	Staphylococcus phage phiSa2wa_st78	Australia	45878	*Staphylococcus aureus*	Curtin University, School of Biomedical Sciences	10/01/2018	Triavirus st78	Triavirus	Caudoviricetes
MG029515.1	Staphylococcus phage phiSa2wa_st80	Australia	45164	*Staphylococcus aureus*	Curtin University, School of Biomedical Sciences	10/01/2018	Staphylococcus phage phiSa2wa_st80	Triavirus	Caudoviricetes
MG029517.1	Staphylococcus phage phiSa2wa_st93	Australia	45913	*Staphylococcus aureus*	Curtin University, School of Biomedical Sciences	10/01/2018	Triavirus P240	Triavirus	Caudoviricetes
MG029516.1	Staphylococcus phage phiSa2wa_st93mssa	Australia	45913	*Staphylococcus aureus*	Curtin University, School of Biomedical Sciences	10/01/2018	Triavirus P240	Triavirus	Caudoviricetes
OP584903.1	Staphylococcus phage phiSa2wa-st1232	Australia	45907	*Staphylococcus aureus*	Curtin University, School of Biomedical Sciences	19/10/2022	Staphylococcus phage phiSa2wa-st1232	Triavirus	Caudoviricetes
OP328422.1	Staphylococcus phage phiSa2wa-st1633	Australia	49642	*Staphylococcus aureus*	Curtin University, School of	09/10/2022	Staphylococcus phage phiSa2wa-st1633	Triavirus	Caudoviricetes

Accession	Organism_Name	Country	Length (bp)	Host	Organization	Release_Date	Species	Genus	Family or Class
					Biomedical Sciences				
OP584904.1	Staphylococcus phage phiSa2wa-st2974	Australia	45452	*Staphylococcus aureus*	Curtin University, School of Biomedical Sciences	19/10/2022	Staphylococcus phage phiSa2wa-st2974	Triavirus	Caudoviricetes
ON989481.1	Staphylococcus phage phiSa2wa-st5.2	Australia	44811	*Staphylococcus aureus*	Curtin University, School of Biomedical Sciences	17/08/2022	Staphylococcus phage phiSa2wa-st5.2	Triavirus	Caudoviricetes
OP038318.1	Staphylococcus phage phiSa2wa-st5.3	Australia	44811	*Staphylococcus aureus*	Curtin University, School of Biomedical Sciences	02/10/2022	Staphylococcus phage phiSa2wa-st5.3	Triavirus	Caudoviricetes
OP038319.1	Staphylococcus phage phiSa2wa-st5.4	Australia	44811	*Staphylococcus aureus*	Curtin University, School of Biomedical Sciences	02/10/2022	Staphylococcus phage phiSa2wa-st5.4	Triavirus	Caudoviricetes
OP136131.1	Staphylococcus phage phiSa2wa-st5.7	Australia	47013	*Staphylococcus aureus*	Curtin University, School of Biomedical Sciences	26/10/2022	Staphylococcus phage phiSa2wa-st5.7	Triavirus	Caudoviricetes
OP136132.1	Staphylococcus phage phiSa2wa-st6	Australia	45270	*Staphylococcus aureus*	Curtin University, School of Biomedical Sciences	26/10/2022	Staphylococcus phage phiSa2wa-st6	Triavirus	Caudoviricetes
OP328420.1	Staphylococcus phage phiSa2wa-st923	Australia	45914	*Staphylococcus aureus*	Curtin University, School of Biomedical Sciences	08/10/2022	Staphylococcus phage phiSa2wa-st923	Triavirus	Caudoviricetes
OP136134.1	Staphylococcus phage Sa2wa-st88	Australia	45346	*Staphylococcus aureus*	Curtin University, School of Biomedical Sciences	26/10/2022	Staphylococcus phage Sa2wa-st88	Triavirus	Caudoviricetes
JX174275.1	Staphylococcus phage LH1	Canada	46048	*Staphylococcus aureus*	Universite Laval, Biochimie, Microbiologie et Bio-Informatique	05/03/2013	Triavirus LH1	Triavirus	Caudoviricetes
KT186243.1	Staphylococcus phage vB_SauS_phi2	Canada	44222	*Staphylococcus aureus*	Universite Laval, Biochemistry, Microbiology, and Bio-informatics	09/08/2015	Triavirus tv2	Triavirus	Caudoviricetes
MT899504.1	Staphylococcus phage JD419	China	45509	*Staphylococcus aureus*	Shanghai General Hospital, Department of Medicine Laboratory	27/11/2020	Staphylococcus phage JD419	Triavirus	Caudoviricetes

Accession	Organism_Name	Country	Length (bp)	Host	Organization	Release_Date	Species	Genus	Family or Class
MG770897.1	Staphylococcus phage SH-St 15644	China	45111	*Staphylococcus aureus*	Shanghai Jiao Tong University School of Medicine, Department of Microbiology and Immunology	03/02/2018	Triavirus tv15644	Triavirus	Caudoviricetes
LC541428.1	Staphylococcus phage vB_SauS_JS02	China	46435	*Staphylococcus aureus*	Jiangsu Academy of Agricultural Sciences., State Key Laboratory Cultivation Base of MOST.; No. 50 Zhongling Street, Nanjing, Jiangsu 210014	17/04/2020	Triavirus JS02	Triavirus	Caudoviricetes
OP797801.1	Staphylococcus phage vB_SauS_SPG4	China	46416	*Staphylococcus aureus*	Shanghai Ocean University, College of Food Science and Technology	30/11/2022	Staphylococcus phage vB_SauS_SPG4	Triavirus	Caudoviricetes
MK348510.1	Staphylococcus phage vB_SauS_fPfSau02	Finland	45108	*Staphylococcus aureus*	University of Helsinki, Research Programs Unit	06/02/2019	Triavirus fPfSau02	Triavirus	Caudoviricetes
JQ957932.1	Staphylococcus phage StauST398-2	France	45572	*Staphylococcus aureus*	University of Geneva Hospitals, Genomic Research Laboratory	30/04/2013	Triavirus StauST3982	Triavirus	Caudoviricetes
KY056620.1	Staphylococcus phage P240	Germany	45985	*Staphylococcus aureus*	Federal Institute for Risk Assessment, Biological Safety	24/05/2017	Triavirus P240	Triavirus	Caudoviricetes
ON856257.1	Staphylococcus phage 1PHSA12	NA	50643	*Staphylococcus aureus*	Huazhong Agricultural University, State Key Laboratory of Agricultural Microbiology	24/08/2022	Staphylococcus phage 1PHSA12	Triavirus	Caudoviricetes
OL690335.1	Staphylococcus phage 2PHSA1	NA	59779	*Staphylococcus aureus*	Huazhong Agricultural University, State Key Laboratory of Agricultural Microbiology	03/01/2022	Staphylococcus phage 2PHSA1	Triavirus	Caudoviricetes
OM304856.1	Staphylococcus phage 4PHCISA25	NA	45086	*Staphylococcus aureus*	Huazhong Agricultural University, State Key Laboratory of	09/03/2022	Staphylococcus phage 4PHCISA25	Triavirus	Caudoviricetes

Accession	Organism_Name	Country	Length (bp)	Host	Organization	Release_Date	Species	Genus	Family or Class
					Agricultural Microbiology				
ON856258.1	Staphylococcus phage A12-4PHSA25	NA	45314	*Staphylococcus aureus*	Huazhong Agricultural University, State Key Laboratory of Agricultural Microbiology	24/08/2022	Staphylococcus phage A12-4PHSA25	Triavirus	Caudoviricetes
CP062444.1	Staphylococcus phage ECel-2020e	NA	44566	*Staphylococcus aureus*	University of central Florida, Burnett School of Biomedical Sciences	07/03/2022	Staphylococcus phage ECel-2020e	Triavirus	Caudoviricetes
CP062442.1	Staphylococcus phage ECel-2020f	NA	45361	*Staphylococcus aureus*	University of central Florida, Burnett School of Biomedical Sciences	07/03/2022	Staphylococcus phage ECel-2020f	Triavirus	Caudoviricetes
CP062434.1	Staphylococcus phage ECel-2020h	NA	44566	*Staphylococcus aureus*	University of central Florida, Burnett School of Biomedical Sciences	07/03/2022	Staphylococcus phage ECel-2020h	Triavirus	Caudoviricetes
CP062461.1	Staphylococcus phage ECel-2020m	NA	45676	*Staphylococcus aureus*	University of central Florida, Burnett School of Biomedical Sciences	07/03/2022	Staphylococcus phage ECel-2020m	Triavirus	Caudoviricetes
MW924497.1	Staphylococcus phage PHB21	NA	45260	*Staphylococcus aureus*	College of Veterinary Medicine, State Key Laboratory of Agricultural Microbiology, Huazhong Agricultural University	31/05/2021	Staphylococcus phage PHB21	Triavirus	Caudoviricetes
AF424782.1	Staphylococcus phage phi 12	NA	44970	*Staphylococcus aureus*	Univ. of Oklahoma Health Sciences Center, Microbiology and Immunology	26/02/2002	Triavirus tv12	Triavirus	Caudoviricetes
NC_011344.1	Staphylococcus phage phi2958PVL	NA	47342	*Staphylococcus aureus*	National Center for Biotechnology Information, NIH	09/10/2008	Triavirus tv2958PVL	Triavirus	Caudoviricetes
OP374154.1	Staphylococcus phage phiSa2wa-st5.5	NA	44811	*Staphylococcus aureus*	Curtin University, School of	26/10/2022	Staphylococcus phage phiSa2wa-st5.5	Triavirus	Caudoviricetes

Accession	Organism_Name	Country	Length (bp)	Host	Organization	Release_Date	Species	Genus	Family or Class
					Biomedical Sciences				
OP374155.1	Staphylococcus phage phiSa2wa-st5.8	NA	44811	*Staphylococcus aureus*	Curtin University, School of Biomedical Sciences	26/10/2022	Staphylococcus phage phiSa2wa-st5.8	Triavirus	Caudoviricetes
OP374156.1	Staphylococcus phage phiSa2wa-st5.9	NA	44811	*Staphylococcus aureus*	Curtin University, School of Biomedical Sciences	26/10/2022	Staphylococcus phage phiSa2wa-st5.9	Triavirus	Caudoviricetes
EU861005.1	Staphylococcus phage phiSauS-IPLA35	NA	45344	*Staphylococcus aureus*	CSIC, Instituto de Productos Lacteos de Asturias	01/12/2008	Triavirus IPLA35	Triavirus	Caudoviricetes
MT366568.1	Staphylococcus phage R4	NA	45168	*Staphylococcus aureus*	Enviroinvest Corp.	19/05/2020	Staphylococcus phage R4	Triavirus	Caudoviricetes
OL840290.1	Staphylococcus phage Rih21	NA	44789	*Staphylococcus aureus*	Al Nahrain University, Biotechnology	31/01/2022	Staphylococcus phage Rih21	Triavirus	Caudoviricetes
MK940809.1	Staphylococcus phage Sa2wa_st8	NA	45914	*Staphylococcus aureus*	Curtin University, School of Biomedical Sciences	15/07/2019	Triavirus P240	Triavirus	Caudoviricetes
MK801681.1	Staphylococcus phage SAP11	NA	45346	*Staphylococcus aureus*	Gachon University, Food Science and Biotechnology	07/07/2019	Triavirus SAP11	Triavirus	Caudoviricetes
MK801680.1	Staphylococcus phage SAP8	NA	45533	*Staphylococcus aureus*	Gachon University, Food Science and Biotechnology	07/07/2019	Staphylococcus phage SAP8	Triavirus	Caudoviricetes
MK075005.1	Staphylococcus phage phiSP119-2	NA	40011	*Staphylococcus pseudintermedius*	The Rockefeller University, Laboratoy of Bacterial Pathogenesis and Immunology	12/04/2019	Staphylococcus phage phiSP119-2	Triavirus	Caudoviricetes
AY954956.1	Staphylococcus phage 3A	NA	43095	*Staphylococcus sp.*	Targanta Therapeutics	05/04/2005	Triavirus tv3a	Triavirus	Caudoviricetes
AY954955.1	Staphylococcus phage 42E	NA	45861	*Staphylococcus sp.*	Targanta Therapeutics	05/04/2005	Triavirus tv42e	Triavirus	Caudoviricetes
AY954957.1	Staphylococcus phage 47	NA	44777	*Staphylococcus sp.*	Targanta Therapeutics	05/04/2005	Triavirus tv47	Triavirus	Caudoviricetes
AP012341.1	Staphylococcus phage phi7401PVL	NA	47252	*Staphylococcus sp.*	Department of Bacteriology; Hongo 2-1-1, Bunkyo-ku, Tokyo 113-8421	25/01/2013	Triavirus JS02	Triavirus	Caudoviricetes
AB045978.2	Staphylococcus phage phiSLT	NA	42942	*Staphylococcus sp.*	Graduate school of Agricultural	07/02/2001	Triavirus SLT	Triavirus	Caudoviricetes

Accession	Organism_Name	Country	Length (bp)	Host	Organization	Release_Date	Species	Genus	Family or Class
					Science; 1-1 Tsutsumidori Amamiyamachi, Sendai, Miyagi 981-8555				
EF462198.1	Staphylococcus phage tp310-2	NA	47785	*Staphylococcus sp.*	University of Regensburg, Institute of Medical Microbiology and Hygiene	13/08/2007	Triavirus tp3102	Triavirus	Caudoviricetes
KF598856.1	Staphylococcus phage YMC/09/04/R1988	NA	44459	*Staphylococcus sp.*	Yonsei University College of Medicine, Department of Laboratory Medicine and Research Institute of Antimicrobial Resistance	05/11/2013	Triavirus R1988	Triavirus	Caudoviricetes
MH384261.1	Staphylococcus phage SA137ruMSSAST121PVL	Russia	45999	*Staphylococcus aureus*	N.F. Gamaleya National Research Center for Epidemiology and Microbiology Ministry of Health of Russia, Laboratory of Molecular Bases of Pathogenicity	25/07/2018	Triavirus SA137ruMSSAST121PVL	Triavirus	Caudoviricetes
JQ779023.1	Staphylococcus phage SMSAP5	South Korea	45552	*Staphylococcus aureus*	Food Science and Biotechnology, Gachon University	02/05/2012	Triavirus SAP11	Triavirus	Caudoviricetes
OM373559.1	Staphylococcus phage vB_SpsX-DS12	USA	43755	*Staphylococcus pseudintermedius*	Baylor College of Medicine, Molecular Virology and Microbiology	30/03/2022	Staphylococcus phage vB_SpsX-DS12	Triavirus	Caudoviricetes
AY954970.1	Staphylococcus phage Twort	NA	130706	*Staphylococcus sp.*	Targanta Therapeutics	05/04/2005	Twortvirus twort	Twortvirus	Herelleviridae
KY653126.1	Staphylococcus virus IME1354_01	NA	42706	*Staphylococcus sp.*	Beijing Institute of Microbiology and Epidemiology, State Key Laboratory of Pathogen	06/05/2017	Zhangqianvirus IME1354	Zhangqianvirus	Caudoviricetes
