# Supplementary material for: Staphylococcal phages as agents of evolution and innovation: From gene flow to next-generation therapeutics
Source: Genet Mol Biol. 2026 Feb 9;49(Suppl 1):e20250131. doi: 10.1590/1678-4685-GMB-2025-0131 (PMC12895238; doi:10.1590/1678-4685-GMB-2025-0131)
Supplement: Chart S1 - [file 1415-4757-GMB-49-s1-e20250131-s2.pdf]

Genetics and Molecular Biology
DOI: https://doi.org/10.1590/1678-4685-GMB-2025-0131

Supplementary Material to “Staphylococcal phages as agents of evolution and innovation: From gene flow to next-generation therapeutics”

Chart S1 - Examples of companies working on the development of commercial phage therapy.

Company	Country	Description	Business Model	Classification Rationale	Exclusion from Other Categories	Main Market(s)	Key Feature	Staphylococcus	Website
Locus Biosciences	USA	Locus Biosciences is a clinical-stage biotechnology company headquartered in North Carolina, USA, that develops CRISPR-enhanced bacteriophage therapies targeting multidrug-resistant bacterial infections. Its proprietary LOCUS platform integrates synthetic biology, high-throughput screening, AI-driven design, and GMP manufacturing to engineer phages with CRISPR-Cas3 payloads. The company’s business model combines in-house therapeutic development with strategic partnerships, such as a collaboration with Janssen Pharmaceuticals worth up to $818 million. Revenue is generated through milestone payments, public funding (e.g., BARDA), and venture capital investments from firms like Arch Venture Partners.	End-to-End Developer	Locus Biosciences fits the “End-to-End Developer” model, as it maintains full vertical integration of its product development pipeline—from phage engineering and preclinical studies to clinical trials and GMP manufacturing. It develops standardized, scalable therapeutic products intended for regulatory approval and global commercialization.	Locus does not align with the “Application-Focused Distributor” category, as it is not active in agriculture, food safety, or veterinary markets, and does not operate a B2B product deployment strategy. Nor does it fit the “Scalable and Regulated Access” or “Magistral and On-Demand Therapy” models, as it does not pursue non-traditional regulatory routes, adaptive deployment schemes, or personalized phage compounding. Instead, it follows a classical pharmaceutical development path.	Locus focuses primarily on human therapeutics, addressing urgent medical needs in infectious diseases, immunology, and microbiome-related conditions. Its clinical programs target pathogens such as Escherichia coli, Klebsiella pneumoniae, Pseudomonas aeruginosa, and Staphylococcus aureus. In addition, it explores microbiome modulation strategies for inflammatory diseases like Crohn’s disease and potential applications in oncology and neurology.	A key differentiator of Locus is its use of CRISPR-Cas3, a nuclease system that induces large-scale DNA degradation in bacteria, offering a higher level of bactericidal efficiency than CRISPR-Cas9 approaches. Its therapies are designed for species-specific precision, preserving beneficial microbiota. The integration of a proprietary tech platform with internal cGMP manufacturing capabilities accelerates development while ensuring regulatory compliance and scalability.	In the domain of Staphylococcus aureus, Locus is actively developing phage therapies targeting antibiotic-resistant strains, including MRSA. These programs leverage the CRISPR-Cas3 system to selectively degrade the pathogen’s genome, aiming for high efficacy and microbiome preservation. While its most advanced clinical candidate targets E. coli, S. aureus remains a key target in its broader infectious disease pipeline, particularly due to its relevance in hospital-acquired infections and its resistance profile.	https://www.locus-bio.com
BiomX	Israel / USA	BiomX is a clinical-stage biotechnology company developing phage-based therapeutics for chronic and acute infections. The company designs phage-based therapies targeting pathogenic bacteria implicated in chronic diseases. It operates a vertically integrated pipeline encompassing proprietary phage discovery, genome engineering (including CRISPR-based payloads), preclinical validation, GMP-compliant production, and clinical development. Its core	End-to-End Developer	BiomX fits the “End-to-End Developers” model due to its comprehensive in-house capabilities spanning the full phage therapy development lifecycle—from isolation and optimization to clinical testing. The company retains proprietary control over both its platform and clinical pipeline, with therapies progressing through conventional regulatory stages, including advanced trials in cystic fibrosis and diabetic foot infections. The 2024 acquisition of	BiomX is not primarily focused on the distribution or sector-specific deployment of phages, which excludes it from the “Application-Focused Distributors” category. Nor does it rely on hospital-based compounding or magistral production, ruling out “Magistral and On-Demand Therapy Models.” Although it collaborates with academic institutions and leverages grants, its regulatory and operational model does not align with the hybridized pathways typical of “Scalable and Regulated	The company targets high-unmet-need therapeutic markets, including pulmonary infections in cystic fibrosis (BX004), diabetic foot osteomyelitis caused by antibiotic-resistant Staphylococcus aureus (BX211), atopic dermatitis, and inflammatory bowel diseases. These indications reflect a strategic focus on areas where antibiotic resistance or dysbiosis plays a central role in disease pathology and	BiomX’s key differentiators include its modular and scalable phage engineering platform, integration of machine learning for rapid host-pathogen mapping, and the capacity to genetically tailor phages for enhanced selectivity and potency. Its ability to internalize both discovery and development allows it to maintain agility and control across regulatory and therapeutic domains.	BiomX is actively developing phage-based therapies targeting Staphylococcus aureus, particularly in the context of chronic wound infections such as diabetic foot osteomyelitis, where multidrug resistance limits the efficacy of conventional antibiotics. The company’s BX211 program applies precision phage selection and formulation techniques to address patient-specific infections, with the goal of minimizing	https://www.biomx.com

Company	Country	Description	Business Model	Classification Rationale	Exclusion from Other Categories	Main Market(s)	Key Feature	Staphylococcus	Website
		platform integrates AI-powered bioinformatics with synthetic biology to rapidly match therapeutic phages to specific bacterial strains. In March 2024, BiomX completed the acquisition of Adaptive Phage Therapeutics (APT), incorporating the PhageBank™ platform and U.S. clinical network into its end-to-end R&D and manufacturing operations. BiomX's revenue model is currently centered on R&D collaborations, milestone-driven partnerships, technology licensing, and public funding to support its therapeutic programs.		APT further strengthens this vertical integration while expanding its capacity for rapid, regulated phage deployment within a centralized development framework.	Access Models," as it maintains centralized, controlled development rather than adaptive or decentralized access.	where phage precision offers a competitive advantage over broad-spectrum antimicrobials.	Collaborations with leading research institutes further reinforce its scientific rigor and innovation pipeline.	inflammation and promoting healing in complex soft-tissue infections. Following the acquisition of APT, BiomX now combines its proprietary BX004 and BX211 programs with APT's PhageBank™, advancing from preclinical optimization to early-stage compassionate-use deployments. Previous programs have also explored the role of S. aureus in dermatological indications, including atopic dermatitis, where phage therapy may help rebalance the skin microbiome and reduce flare severity.	
Armata Pharmaceuticals	USA	Armata Pharmaceuticals is a clinical-stage biotechnology company based in Los Angeles, California, focused on the development of bacteriophage-based therapeutics for antibiotic-resistant infections. The company operates a fully integrated phage platform that spans from discovery and synthetic engineering to formulation, cGMP manufacturing, and clinical development. Its business model centers on the proprietary development of phage therapies, with revenue expected from future product commercialization, licensing agreements, and non-dilutive funding sources. Currently, Armata generates income primarily through government grants and institutional partnerships that support its clinical programs.	End-to-End Developer	Armata is best classified as an End-to-End Developer due to its complete vertical integration across the phage development pipeline. The company retains proprietary control over its therapeutic candidates and conducts all key stages internally, from phage isolation and engineering to regulatory-grade manufacturing and clinical trials. This model reflects a pharmaceutical approach aimed at securing market authorization for standardized, scalable phage-based drugs targeting unmet medical needs.	Armata does not operate as an Application-Focused Distributor, since it does not engage in sector-specific distribution outside human health nor pursue rapid commercialization in lower-regulation markets such as agriculture or food safety. It also diverges from Scalable and Regulated Access Models, which emphasize flexible deployment and public-private consortia—while Armata benefits from government funding, its regulatory strategy adheres strictly to conventional drug approval pathways. Finally, it does not fit within Magistral and On-Demand Therapy Models, as it does not engage in personalized or compounded treatments delivered through hospital networks or local infrastructure.	Armata targets infectious diseases caused by multidrug-resistant bacteria, with a specific focus on serious, hard-to-treat clinical indications. These include chronic pulmonary infections—especially in cystic fibrosis patients—caused by Pseudomonas aeruginosa, and bloodstream infections caused by Staphylococcus aureus. These therapeutic areas represent significant unmet medical needs, particularly in hospital and critical care settings, and align with global health priorities for new anti-infective solutions.	Armata's main strategic advantages lie in its fully integrated infrastructure, allowing for rapid and controlled development of phage therapies from concept to clinical stage. The company employs advanced synthetic biology to engineer bacteriophages with enhanced therapeutic traits, including improved host specificity and biofilm penetration. Its high-purity phage production capabilities meet regulatory standards for clinical use, and its engagement with U.S. government agencies provides both financial support and strategic alignment with national biodefense initiatives.	In the Staphylococcus domain, Armata is developing AP-SA02, a clinical-stage phage cocktail targeting Staphylococcus aureus, including methicillin-resistant strains (MRSA). The candidate is being evaluated for use in bacteremia, a severe bloodstream infection associated with high morbidity and mortality. Supported by a $21.6 million grant from the U.S. Department of Defense, AP-SA02 is undergoing clinical trials designed to assess safety, efficacy, and pharmacokinetics in hospitalized patients. This positions Armata as a key player in advancing phage therapy for one of the most clinically challenging Gram-positive pathogens.	https://www.armatapharma.com
Intralytix	USA	Intralytix is a vertically integrated biotechnology company headquartered in Maryland, USA, that develops, manufactures, and commercializes bacteriophage-based products. Its operations span from phage isolation	End-to-End Developer	Intralytix fits the End-to-End Developer category because it maintains full control over the phage development lifecycle. From early-stage discovery and bioinformatics-driven selection to formulation,	Although Intralytix has a strong commercial presence in food safety—characteristic of Application-Focused Distributors—its core innovation lies in deep R&D and proprietary clinical assets. Unlike	The company addresses several verticals: (1) food safety, with FDA-approved phage cocktails such as ListShield™ and SalmoFresh™ used to reduce pathogens like Listeria, Salmonella, and	Intralytix was the first company to obtain FDA clearance for a phage-based product in food safety and holds the largest portfolio of such approvals globally. Its proprietary AI-assisted	Intralytix's work against Staphylococcus aureus includes the development of EcoActive™, a phage preparation currently in Phase 1/2a trials targeting VRE and methicillin-resistant Staphylococcus aureus	https://www.intralytix.com

Company	Country	Description	Business Model	Classification Rationale	Exclusion from Other Categories	Main Market(s)	Key Feature	Staphylococcus	Website
		and characterization to GMP-grade production and clinical development. The company combines proprietary platforms such as PhageSelector™ and PhageEngine™, which utilize machine learning for phage optimization, with in-house regulatory, formulation, and manufacturing capabilities. Intralytix generates revenue through multiple channels: direct product sales (particularly in food safety), research grants (e.g., NIH SBIR contracts), and strategic partnerships, such as its collaboration with Lesaffre for global commercialization and co-development of phage products.		regulatory approval, and clinical trials, the company consolidates all critical stages of the value chain. Its commitment to developing therapeutic phage applications for human health, including multiple clinical-stage programs, further supports this classification.	Scalable Access Models, Intralytix does not rely primarily on public or institutional deployment schemes, nor does it operate under flexible regulatory frameworks like compassionate use. It also diverges from Magistral and On-Demand Therapy Models, as it focuses on standardized, approved products rather than individualized, extemporaneous therapies.	E. coli in food processing; (2) human therapeutics, with investigational treatments for infections caused by Shigella, Enterococcus (VRE), and Clostridioides difficile; (3) animal health and agriculture, with less-publicized but active development programs; and (4) environmental decontamination, including phage applications for industrial sanitation and pathogen control.	discovery and manufacturing platforms provide a scalable and adaptive R&D pipeline. In addition, the company has secured Halal, Kosher, and OMRI certifications, and maintains a GMP-compliant facility for phage production. Strategic alliances, particularly with multinational players like Lesaffre and Ferring, enhance its global reach and validate its commercial potential.	(MRSA) colonization. While the company's food safety products do not explicitly target S. aureus, its therapeutic pipeline addresses staphylococcal infections as part of broader anti-AMR efforts. The company applies its platform technologies to identify effective phage combinations for staphylococcal strains and is positioned to expand its clinical indications in this area based on the outcomes of its ongoing human trials.	
Proteon Pharmaceuticals	Poland	Proteon Pharmaceuticals is a Polish biotechnology company operating a vertically integrated platform focused on the development and commercialization of bacteriophage-based feed additives for animal health. Its proprietary phage platform leverages artificial intelligence, bioinformatics, genomics, and molecular biology to target specific bacterial pathogens in livestock, aquaculture, and dairy systems. The company generates revenue through global B2B sales of ready-to-use phage products, such as BAFASAL® and BAFADOR®, designed for inclusion in animal feeding or water systems. Proteon's model emphasizes scale, regulatory compliance, and product standardization for industrial use.	Application-Focused Distributor	Proteon Pharmaceuticals fits into the "Application-Focused Distributor" category, as it concentrates on the formulation, regulatory registration, and sector-specific deployment of phage-based products in non-clinical markets. The company develops commercial solutions tailored for animal production systems, aligning with shorter regulatory timelines and a business-to-business distribution strategy. Its focus on industrial-scale delivery of standardized phage cocktails reinforces this classification.	Proteon does not fit the "End-to-End Developer" model because it does not engage in GMP-level manufacturing for human therapeutics or pursue pharmaceutical clinical trials. Nor does it align with the "Scalable and Regulated Access Model," as it does not rely on adaptive regulatory pathways or public-private partnerships in human health contexts. It is also distinct from "Magistral and On-Demand Therapy Models," since it produces off-the-shelf products rather than personalized or compounded phage preparations.	The company primarily targets the poultry, aquaculture, and dairy industries. Its products aim to reduce pathogenic load, improve gut health, and enhance productivity, particularly in industrial farming environments where minimizing antibiotic use is a priority. With approvals in several regions and active international expansion, Proteon addresses global markets facing increasing pressure to reduce antimicrobial resistance in food production chains.	Proteon's key differentiators include its AI-powered phage selection and optimization platform, regulatory leadership in phage-based feed additives, and its dedication to sustainable farming. It was among the first to submit bacteriophage dossiers to the European Food Safety Authority (EFSA), setting a regulatory precedent in the sector. The recent launch of its Bacteriophage Biotechnology Center further consolidates its position as a technological leader in the animal health phage market.	While Proteon Pharmaceuticals primarily focuses on pathogens such as Salmonella and Aeromonas, its technological platform is adaptable to a range of bacterial targets, including Staphylococcus species. Although the company does not currently market a phage product specifically for Staphylococcus, its phage discovery and bioinformatics infrastructure positions it to expand into this area should market demand arise, particularly in bovine mastitis or other livestock-associated Staphylococcus infections.	https://www.proteonpharma.com
Phagelux	China/USA	Phagelux AgriHealth Inc. is a biotechnology company focused on the development, registration, and commercialization of bacteriophage-based solutions for sustainable agriculture, animal health, and food safety. Operating	Application-Focused Distributor	Phagelux fits the Application-Focused Distributors category, as it prioritizes the development and deployment of phage-based biocontrol agents tailored to specific sectors like crop protection,	Phagelux does not align with End-to-End Developers, as it does not primarily focus on human therapeutic pipelines or clinical trials. It also does not operate under Scalable and Regulated Access Models, which rely on	The company's target markets include commercial agriculture (fruit and vegetable growers seeking alternatives to chemical pesticides), animal health (poultry, swine, and cattle producers	Phagelux's competitive edge lies in its proprietary phage-based formulations, its track record of product registration across multiple countries, and its dual R&D-	While Phagelux's primary commercial focus is in agriculture and animal health, its broader R&D portfolio includes phage solutions targeting Staphylococcus spp. in veterinary contexts, particularly for	https://phage.com

Company	Country	Description	Business Model	Classification Rationale	Exclusion from Other Categories	Main Market(s)	Key Feature	Staphylococcus	Website
		from R&D centers in Nanjing (China) and Salt Lake City (USA), and with manufacturing facilities in North America and Asia, the company combines in-house phage discovery with product formulation and global distribution. Its revenue is generated through direct sales of registered products (e.g., AgriPhage™), strategic partnerships, and licensing agreements in multiple international markets.		livestock disease management, and microbial food contamination. The company operates under business-to-business models, emphasizing scalable, field-ready formulations and streamlined regulatory pathways—characteristics typical of this category.	hybrid regulatory schemes and patient access frameworks, nor under Magistral and On-Demand Therapy Models, which are designed for highly personalized or hospital-compounded treatments, usually in clinical or compassionate-use contexts.	looking to reduce antibiotic use), and food processing companies that require safe and effective microbial control technologies. It positions itself as a scalable provider of environmentally friendly biocontrol tools.	manufacturing footprint in both Asia and North America. Its ability to deliver phage products that are organic-compliant, highly specific, and resistant-management friendly allows the company to address global concerns around antimicrobial resistance and sustainable production practices.	skin and wound infections in livestock. Though not a primary clinical developer, the company contributes to Staphylococcus control through preventive and therapeutic phage formulations designed to improve animal welfare and reduce zoonotic bacterial transmission in farming systems. Its candidate against Staphylococcus aureus, however, remains at the pre-clinical research stage.	
Microbiotec	Brazil	Microbiotec is a Brazilian biotechnology company specializing in phage-based biocontrol solutions, with operations centered around applied microbiological research, custom product development, and specialized diagnostic services. Its core platform leverages lytic bacteriophages isolated from natural environments, which are formulated into sector-specific products for microbial control. The company operates under a B2B model, generating revenue through the sale of proprietary phage products—such as Phage Mast, Salmofagos, and Phage Shield—as well as through contract microbiology services and joint development agreements.	Application-Focused Distributor	Microbiotec is best categorized as an Application-Focused Distributor, as it formulates and delivers phage-based products tailored to defined industrial sectors such as animal health, food safety, and petrochemical infrastructure. Its business strategy emphasizes the translation of phage discovery into deployable solutions rather than pursuing clinical drug development or platform licensing, and it targets industries with relatively streamlined regulatory frameworks.	While Microbiotec engages in in-house phage isolation and product design, it does not pursue end-to-end vertical integration that includes GMP manufacturing or human clinical trials, which excludes it from the End-to-End Developer category. Likewise, it does not participate in regulated access programs or hybrid models for clinical deployment, nor does it offer individualized phage therapies or operate under magistral frameworks, precluding classification as either a Scalable Access Model or an On-Demand Therapy provider.	The company's phage solutions are directed toward several key markets: animal health (notably poultry and dairy), agribusiness, food production, and industrial sectors such as oil and gas, where biofilm formation and biocorrosion pose persistent challenges. Microbiotec also provides microbial diagnostics and consulting services to pharmaceutical and veterinary clients.	Microbiotec's strategic edge lies in its ability to deliver phage-based alternatives to antibiotics and chemical biocides, offering environmentally friendly and highly specific solutions. Its capacity for product customization, rapid deployment in industrial settings, and its integration of microbiology services strengthen its value proposition. The company also benefits from close collaboration with academic institutions, enabling agile R&D and access to phage biodiversity.	Within the scope of Staphylococcus control, Microbiotec develops and markets phage-based formulations targeting strains involved in bovine mastitis and skin infections. For example, Phage Mast is designed to combat Staphylococcus aureus in dairy herds, providing a non-antibiotic option for treating mastitis. The company also explores topical and hygiene applications, such as phage-infused ointments and oral rinses aimed at reducing Staphylococcus colonization on skin and mucosal surfaces, aligning with growing demand for precision antimicrobials in both veterinary and human-use contexts.	https://microbiotec.bio
Eligo Bioscience	France	Eligo Bioscience is a clinical-stage biotechnology company headquartered in Paris, focused on precision microbiome editing. Its proprietary platform, Eligobiotics®, employs phage-like particles to deliver therapeutic DNA payloads—such as CRISPR systems—directly to specific bacterial populations within the human microbiome. The company operates through a hybrid model that combines internal drug	Scalable and Regulated Access Models	Eligo is best classified under Scalable and Regulated Access Models, as it integrates cutting-edge biotechnological tools with regulated therapeutic development while leveraging partnerships and modular applications to enable broader access. Rather than pursuing full vertical integration, Eligo strategically focuses on platform scalability and regulatory adaptability, particularly in targeting diseases with unmet	Eligo does not align with the End-to-End Developer model because it does not retain full control over manufacturing, clinical trials, and commercialization pipelines for a broad portfolio of therapeutics. Nor does it function as an Application-Focused Distributor, given its clinical focus and absence from sectors such as agriculture or food safety. Additionally, it does not operate under the Magistral or On-Demand	Eligo targets therapeutic areas where bacterial gene expression contributes to disease pathogenesis. This includes chronic inflammatory conditions like acne vulgaris, infectious diseases involving antibiotic resistance, and cancer immunotherapy modulation. Its programs are designed to offer precise bacterial gene modulation in indications where microbiome-targeted	Eligo's main strategic advantage lies in its ability to edit the microbiome in vivo with unprecedented specificity. Its platform does not merely eliminate pathogenic bacteria, but can selectively modify bacterial functions, offering both antimicrobial and gene-silencing capabilities. This modularity enables Eligo to adapt its core technology to various	Eligo's technology platform is well-suited for precision targeting of pathogenic bacteria such as Staphylococcus aureus. Although S. aureus is not the primary focus of its lead programs, the Eligobiotics® system is designed to be modular and adaptable, capable of delivering CRISPR-based gene editing payloads to selectively eliminate Staphylococcus strains harboring antibiotic resistance or	https://eligo.bio

Company	Country	Description	Business Model	Classification Rationale	Exclusion from Other Categories	Main Market(s)	Key Feature	Staphylococcus	Website
		development with strategic partnerships and licensing agreements. Revenue is generated primarily through R&D collaborations, co-development deals, and platform licensing, with future expectations of product commercialization.		clinical needs driven by microbiome-associated bacterial genes.	Therapy model, as it does not deliver individualized phage formulations or rely on hospital compounding or magistral prescriptions.	interventions can enhance therapeutic outcomes.	therapeutic contexts, positioning it uniquely at the intersection of gene therapy and microbiome science.	virulence factors. This positions the company to develop targeted anti-staphylococcal interventions in future pipelines or collaborative projects.	
Phaxiam Therapeutics	France	PHAXIAM Therapeutics is a French clinical-stage biopharmaceutical company focused on developing and delivering phage-based solutions for severe, antibiotic-resistant bacterial infections. Formed through the 2023 merger of ERYTECH and Pherecydes Pharma, the company operates a dual model: the Phage Therapy Medicinal Products (PTMP) program follows the traditional regulatory route for product registration, while the Individualized Phage Therapies (IPT) program enables hospital-based, GMP-grade phage treatments under magistral and compassionate use frameworks. PHAXIAM generates revenue through these complementary pathways, projecting €28 million in combined sales by 2027 and targeting €200 million by 2030 through a mix of personalized and standardized products.	Scalable and Regulated Access Models	PHAXIAM aligns best with the "Scalable and Regulated Access Models" archetype due to its deliberate integration of flexible access routes and rigorous pharmaceutical development. The company balances real-world deployment via individualized therapies with long-term value creation through regulatory approvals and clinical trials. This hybrid but structured approach enables broader and faster patient access without compromising on scientific and regulatory standards.	Although PHAXIAM possesses end-to-end capabilities in phage discovery, GMP production, and clinical development, it diverges from the "End-to-End Developers" model by heavily leveraging non-traditional access pathways like IPT. Conversely, it does not operate in non-therapeutic sectors, excluding it from the "Application-Focused Distributors" category. Finally, while it offers magistral treatments, the presence of a robust clinical pipeline and regulatory ambition sets it apart from models solely centered on on-demand or hospital-based formulations.	PHAXIAM targets hospital-acquired and resistant infections caused by Staphylococcus aureus, Escherichia coli, and Pseudomonas aeruginosa, all of which are classified by the WHO as critical pathogens. Its commercial strategy focuses on high-income healthcare systems, particularly in Europe and the United States, where both regulatory flexibility for IPT and reimbursement mechanisms for PTMP are more mature.	PHAXIAM's key differentiators include its dual business model (IPT + PTMP), a growing library of GMP-grade phages, and strategic partnerships such as the one with Technophage to expand phage access and susceptibility testing. The company also leverages a proprietary diagnostic platform for phagogram development, enabling personalized and effective phage matching. This integrated offering positions PHAXIAM to lead both in acute clinical responsiveness and scalable, regulated deployment.	PHAXIAM maintains an advanced clinical program focused on Staphylococcus aureus, including the GLORIA Phase II trial for prosthetic joint infections (PJIs), which is the first randomized, placebo-controlled global study of phage therapy in this indication. In parallel, its IPT model supports on-demand treatment of S. aureus infections in hospital settings across Europe, particularly for cases where antibiotic resistance limits therapeutic options. Through its combined clinical and magistral strategies, the company addresses both unmet needs and scalability in staphylococcal infection management.	https://phaxiam.com/en/phagotherapy-en
Micreos	Netherlands/Switzerland	Micreos is a biotechnology company operating through three main divisions: Micreos Pharmaceuticals, Gladskin, and PhageGuard. Its technological platform centers on the use of endolysins—enzymatic alternatives to antibiotics that selectively target bacterial pathogens such as Staphylococcus aureus. The company combines pharmaceutical R&D with consumer-facing dermatological products and industrial phage applications. Revenue is	Scalable and Regulated Access Models	Micreos fits within the "Scalable and Regulated Access Models" category due to its multifaceted strategy that blends traditional clinical development with alternative regulatory routes. Rather than pursuing a fully integrated pharmaceutical pathway alone, the company strategically expands access to its antimicrobial solutions across medical, consumer, and industrial markets—leveraging regulatory flexibility to scale deployment while	Micreos does not conform neatly to the "End-to-End Developers" model, as it does not exclusively pursue proprietary clinical pipelines nor vertically integrate all stages of therapeutic development. Similarly, it exceeds the typical scope of "Application-Focused Distributors" by maintaining internal innovation capacity and engaging in early-stage R&D. It also diverges from "Magistral and On-Demand Therapy Models," as its solutions are not	The company targets three primary market segments: (1) human health—particularly dermatology and oncology indications linked to S. aureus colonization; (2) consumer skincare—offering OTC products for conditions like acne, eczema, and rosacea; and (3) food industry safety—providing bacteriophage-based biocontrol agents to mitigate foodborne pathogens such as Listeria and Salmonella.	Micreos' strategic advantages lie in its modular application of endolysin technology across sectors, its ability to preserve the commensal microbiome while eliminating pathogenic bacteria, and its simultaneous navigation of both consumer and clinical regulatory environments. Collaborations with leading academic institutions, such as ETH Zurich, reinforce	Micreos maintains a strong focus on Staphylococcus aureus, with a diversified portfolio of interventions. Through Gladskin, it markets Staphefekt-based OTC products that specifically target S. aureus without harming beneficial skin microbiota. In its pharmaceutical division, it develops clinical-stage endolysin therapies for chronic S. aureus-associated conditions such as atopic dermatitis and cutaneous T-cell	https://www.micreos.com

Company	Country	Description	Business Model	Classification Rationale	Exclusion from Other Categories	Main Market(s)	Key Feature	Staphylococcus	Website
		generated through a hybrid model: over-the-counter (OTC) product sales via Gladskin, B2B contracts in the food safety sector through PhageGuard, and ongoing investment rounds supporting pharmaceutical development.		maintaining scientific and clinical rigor.	individualized or compounded on demand, but rather standardized and designed for broader market distribution.		its innovation pipeline and credibility. The company's flexible model enables early market entry while preserving the long-term potential of clinical development.	lymphoma (CTCL). These solutions aim to address unmet medical needs in both inflammatory and oncologic dermatology. While not currently marketed for industrial use, the company's technological expertise could also be extended to control S. aureus in broader environmental or manufacturing contexts.	
George Eliava Institute	Georgia	The George Eliava Institute operates as a vertically integrated ecosystem encompassing research, phage production, diagnostics, clinical application, and pharmacy services. Its infrastructure includes Eliava BioPreparations (manufacturing), the Eliava Analytical-Diagnostic Center, and the Eliava Phage Therapy Center, enabling seamless transition from lab to bedside. Revenue streams include personalized phage therapy services, off-the-shelf phage cocktails, diagnostic testing, and partnerships with international institutions. While rooted in scientific excellence, its business model leverages patient-centered services rather than large-scale pharmaceutical commercialization.	Magistral & On-Demand Therapy Models	Eliava Institute exemplifies the Magistral and On-Demand Therapy Model through its patient-specific phage treatments, prepared and administered based on individual diagnostics. This model thrives under the flexible regulatory environment in Georgia, where phage preparations can be compounded and dispensed directly through an authorized in-house pharmacy, allowing for rapid, individualized therapeutic intervention.	Although Eliava maintains end-to-end capabilities, it does not follow the traditional pharmaceutical path involving clinical trials, global regulatory filings, or proprietary commercialization. Likewise, it does not focus on sector-based distribution like Application-Focused Distributors, nor does it build large-scale access frameworks typical of Scalable and Regulated Access Models. Its operations remain centered on customized care, rather than scalable product deployment.	The Institute primarily targets the human health sector, focusing on patients suffering from chronic or antibiotic-resistant infections. Its services attract international clientele seeking alternatives to conventional antibiotics. Additional markets include veterinary medicine and public health, though these remain secondary compared to its clinical therapeutic offerings.	Eliava's key differentiators include its century-long legacy in phage research, one of the world's largest phage collections, and the ability to deliver bespoke treatments within days. Its integrated infrastructure—from research to therapy—provides unmatched agility in treating complex infections. Moreover, Georgia's permissive regulatory framework enables patient access models that are not feasible in most Western countries.	In the field of Staphylococcus infections, Eliava offers tailored diagnostics and customized phage cocktails targeting Staphylococcus aureus, including multidrug-resistant strains such as MRSA. The Phage Therapy Center conducts susceptibility testing to identify effective phage candidates from its extensive library. Treatments are then formulated either from existing preparations or as bespoke solutions, depending on the patient's bacterial isolate, supporting use in chronic wounds, osteomyelitis, post-surgical infections, and other persistent staphylococcal conditions.	https://eliava-institute.org/en
